# Ag-doped non–imperfection-enabled uniform memristive neuromorphic device based on van der Waals indium phosphorus sulfide

**DOI:** 10.1126/sciadv.adk9474

**Published:** 2024-03-13

**Authors:** Yesheng Li, Yao Xiong, Baoxing Zhai, Lei Yin, Yiling Yu, Hao Wang, Jun He

**Affiliations:** ^1^ Key Laboratory of Artificial Micro- and Nano-structures of Ministry of Education, and School of Physical and Technology, Wuhan University, Wuhan 430072, China.; ^2^ Suzhou Institute of Wuhan University, Suzhou 215123, China.; ^3^ School of Science, Wuhan University of Technology, Wuhan 430070, China.; ^4^ Institute of Semiconductors, Henan Academy of Sciences, Zhengzhou 450046, China.

## Abstract

Memristors are considered promising energy-efficient artificial intelligence hardware, which can eliminate the von Neumann bottleneck by parallel in-memory computing. The common imperfection-enabled memristors are plagued with critical variability issues impeding their commercialization. Reported approaches to reduce the variability usually sacrifice other performances, e.g., small on/off ratios and high operation currents. Here, we demonstrate an unconventional Ag-doped nonimperfection diffusion channel–enabled memristor in van der Waals indium phosphorus sulfide, which can combine ultralow variabilities with desirable metrics. We achieve operation voltage, resistance, and on/off ratio variations down to 3.8, 2.3, and 6.9% at their extreme values of 0.2 V, 10^11^ ohms, and 10^8^, respectively. Meanwhile, the operation current can be pushed from 1 nA to 1 pA at the scalability limit of 6 nm after Ag doping. Fourteen Boolean logic functions and convolutional image processing are successfully implemented by the memristors, manifesting the potential for logic-in-memory devices and efficient non–von Neumann accelerators.

## Teaser

An Ag-doped nonimperfection strategy can confine filament to combine ultralow variabilities with desirable switching parameters.

## INTRODUCTION

With the approaching limits of Moore’s law, new efficient computational devices and architectures are required to sustain the growth of machine learning and artificial intelligence (AI) (*1*, *2*). Memristors are highlighted as holding potential for energy-efficient AI hardware since they can colocate computation and memory in a single device, and their crossbar array architectures can provide large parallelism for matrix multiplication operations, which is the key computation in most AI models (*2*, *3*). The memristors that rely on the formation/rupture of conductive filaments (CFs) have been the mainstream and are the most likely to be cosmically commercialized, considering their advantages in the high switching speed, low operation voltage, low power consumption, and high integration density (*4*). However, the uncontrolled ion transport imposes severe intrinsic variability of the resistive switching (RS) process, which has been one of the most critical issues that impede their commercialization (*5*, *6*).

In general, the RS behavior is enabled by the imperfections in most memristors. Lattice vacancies and grain boundaries are the most common imperfections, which can directly form the CFs or provide the ion diffusion channel (*5*, *7*). These imperfections are usually randomly distributed and form disordered ion diffusion channels, which should be the essential reason for the nonuniform and inconsistent RS. The low operation voltage (below 0.2 V), small leakage current (<10^−12^ A), low nonvolatile operation current (picoampere-level), high on/off ratio, ultrathin RS media (below 10 nm), and other characteristics are desirable for high energy efficiency and scalability. However, at these extreme values, the switching variability will be more severely affected by the disordered defects, which makes it difficult to combine the low variability with these excellent performances (*7*–*16*). Approaches involving double-layered structures, heterophase grain boundaries, and dopants have been developed. These approaches aim to confine the formation of the CFs with the goal of improving switching uniformity (*16*–*21*). However, these approaches, which induce residual filaments or produce abundant defects around the dopant, usually lead to an obvious increase in conductance at the expense of the limited on/off ratio and operation current. Constructing homogeneous ion diffusion channels should be the more fundamental solution for uniform RS. However, it has been a challenge to fabricate by naturally random imperfections. Further seeking effective strategies is necessary to overcome the variability bottleneck without sacrificing other performances.

Here, we demonstrate an Ag-doped nonimperfection ion diffusion channel–enabled memristive switching, which can address the variability issues and achieve many desirable metrics. Unlike the common imperfection ion channel, the inherent periodic structural vacancies in van der Waals In_3/4_P_2_S_6_ (IPS) serve as the ordered low-energy ion diffusion channel (*22*–*24*). Further using Ag preoccupied structural vacancies instead of the common lattice substitutions to confine the diffusion path, we can achieve low variabilities of 3.8, 2.3, and 6.9% for the low operation voltage (~0.2 V), high off-state resistance (~10^11^ ohms), and big on/off ratio (~10^8^), respectively. Even for the extremely low operation voltage of 0.05 V, the variability can be down to 7.5%. Apart from the uniformity improvement, Ag doping can remarkably reduce the operation current to 1 pA at the scalability limit of 6 nm, which is two orders lower than that of the undoped IPS memristor with a similar thickness. Our strategy can remarkably improve uniformity without sacrificing other performances. On the basis of the well-rounded performance, 14 basic Boolean logic functions are realized. Furthermore, by leveraging on the crossbar array with high yield, kernels with varied sizes and parallel convolutional operation are implemented for multiple image processing tasks and manifest the potential for the development of non–von Neumann hardware. Such excellent performances are superior to state-of-the-art metal oxide and two-dimensional (2D) material–based memristors.

## RESULTS

### Enhanced RS uniformity in the Ag-IPS memristor

**Fig. 1.**
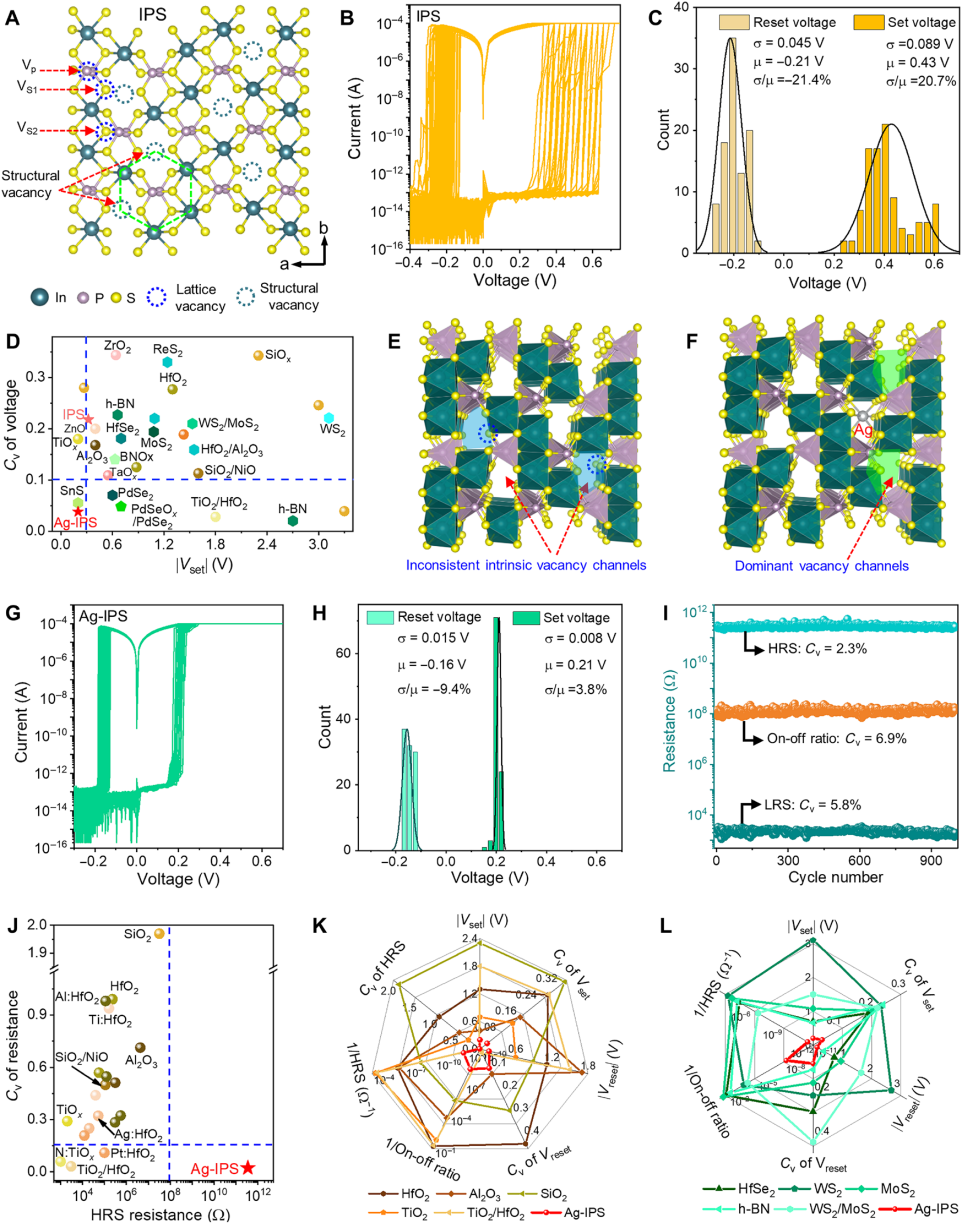
Uniform RS in the Ag-IPS memristor. (**A**) Atomic structure of monolayer IPS showing the intrinsic structural and lattice vacancies. (**B** and **C**) *I*-*V* curves during 100 cycles and the corresponding histogram of the set and reset voltage distributions of IPS (~40 nm) memristor. (**D**) Comparison of the set voltage variation among IPS, Ag-IPS, and reported 2D material– and oxide-based memristors. (**E** and **F**) Perspective view from the *c* axis of multilayer (E) pristine undoped IPS showing intrinsic structural channels with lattice defects and (F) Ag-doped IPS with the intrinsic vacancy occupied by Ag. (**G** and **H**) *I*-*V* curves during 100 cycles and the corresponding histogram of the set and reset voltage distributions of Ag-IPS (~40 nm) memristor. (**I**) Resistance and on/off ratio versus DC endurance cycles of the Ag-IPS device. (**J**) Variation comparison of the HRS resistance among the Ag-IPS and oxide-based memristors. The 2D material–based memristors are not shown here because there are rare articles reporting *C*_v_ of the resistances. Our Ag-IPS memristor has ultrahigh HRS resistance and the lowest variability. (**K** and **L**) Radar graph comparing the device metrics of the Ag-IPS memristor with (K) oxide- and (L) 2D material–based memristors. Our device shows the best overall performance. *C*_V_, coefficient of variation; *C*_V_ = σ/μ, where σ and μ are the SD and mean value of the resistance, respectively. For detailed references, please see tables S1 and S2 [marked in blue for (D), marked in green for (J), and marked in purple for (K) and (L)]. Before the repeated *I*-*V* cycles, electroforming processes are required (fig. S2). The size of the IPS and Ag-IPS memristors is 2 × 2 μm^2^.

In contrast to the common metal phosphorus trichalcogenides with close-packed structures (such as Ni_2_P_2_S_6_ and Mn_2_P_2_S_6_), only four-third metal sites are occupied by indium (In), leaving two-third sites vacant in In_4/3_P_2_S_6_ (IPS), i.e., the structural vacancies as illustrated in Fig. 1A (*22*). Unlike the usual disordered imperfection (such as lattice vacancies and grain boundaries), these structural vacancies (nonimperfection) are periodically arranged. When combined with its AAA stack structure, they form ordered vacancy channels and serve as the shortest ion diffusion pathway. In principle, these structural channels in perfect IPS are identical, which will lead to identical filaments via any channel. Thus, a uniform RS behavior during the repeated cycles can be expected. However, mediocre uniformities are observed in the Ag/IPS (~40 nm)/Au memristor (the structure is illustrated in fig. S1). As shown in Fig. 1 (B and C), the set and reset voltages of the IPS memristor are 0.24 to 0.64 V and −0.12 to −0.3 V, respectively, which results in mean set and reset voltages of 0.43 and −0.21 V and variations of 20.7 and −21.4%, respectively. Such variations show intermediate levels among the reported 2D materials and metal oxide–based memristors, as displayed in Fig. 1D and fig. S3, which indicates obvious variations in RS parameters. The unexpected discrepancies are due to the inevitable random distributed lattice vacancies [especially the sulfur (S) vacancies at the edge of the structural vacancy (i.e., V_S1_)], which makes the ordered vacancy channel inconsistent, as schematically shown in Fig. 1 (A and E).

Impressively, when the structural vacancies are partially presubstituted by Ag (Ag-IPS), as schematically displayed in Fig. 1F, the uniformities of RS parameters are dramatically improved. As shown in Fig. 1 (G and H), the memristor integrated with Ag-IPS (~40 nm) exhibits an extremely uniform bipolar nonvolatile RS behavior with set voltage and reset voltage variations of 3.8 and −9.4%, respectively. Such uniformity and the low operation voltages almost stand alone in the region of the operation voltage <0.3 V and variation <10%, as presented in Fig. 1 (D and H) and fig. S3. The enhanced uniformity can also be found in thinner samples, as shown in fig. S4. Compared with the operation voltages, fewer articles are devoted to improving the uniformity of resistances, as shown in tables S1 and S2. Resistance states with small variations are crucial for high computing accuracy and efficiency, since they represent the weights in computing (*3*). A large resistance in the high-resistance state (HRS) is advantageous for reducing power consumption, eliminating sneak current, lowering operational current, and achieving a high on/off ratio for numerous conductance levels. The resistance in HRS is usually more prone to fluctuate than that in the low-resistance state (LRS), since the current in HRS flows across a random number of defects along the total RS media, whereas the current in LRS flows along the CFs (*6*). The Ag-IPS memristor has a lower mean HRS resistance than the IPS sample, as shown in fig. S5. However, it retains a high value of approximately 10^11^ ohms, contributing to a high on/off ratio up to 10^8^. The resistances demonstrate small variations and good cyclical endurance, where the resistance variations of LRS and HRS over 1000 DC cycles are down to 5.8 and 2.3%, respectively (Fig. 1I). Such low variation at high HRS resistance has occupied the most prominent position among oxide-based memristors, as presented in Fig. 1J. Simultaneously, the small variations in resistance maintain the on/off ratio at 10^8^ with a low dynamic range of 6.9% over 1000 DC switching cycles, as exhibited in Fig. 1I. This value is in contrast to other memristors, where uniformity is achieved but with a small on/off ratio (*19*, *25*–*27*). Good endurance is also achieved using pulses, as the device can normally be SET/RESET with more than 10^4^ switching cycles, as exhibited in fig. S6. Performance bench markings (Fig. 1, K and L) further show that the Ag-IPS memristor can achieve ultralow variabilities of RS parameters at extremely low operation voltages, high HRS resistance, and large on/off ratios, which are notable among the oxide- and 2D material–based nonvolatile memristors. These results indicate that using Ag doping IPS is a superior strategy for excellent uniformity.

**Fig. 2.**
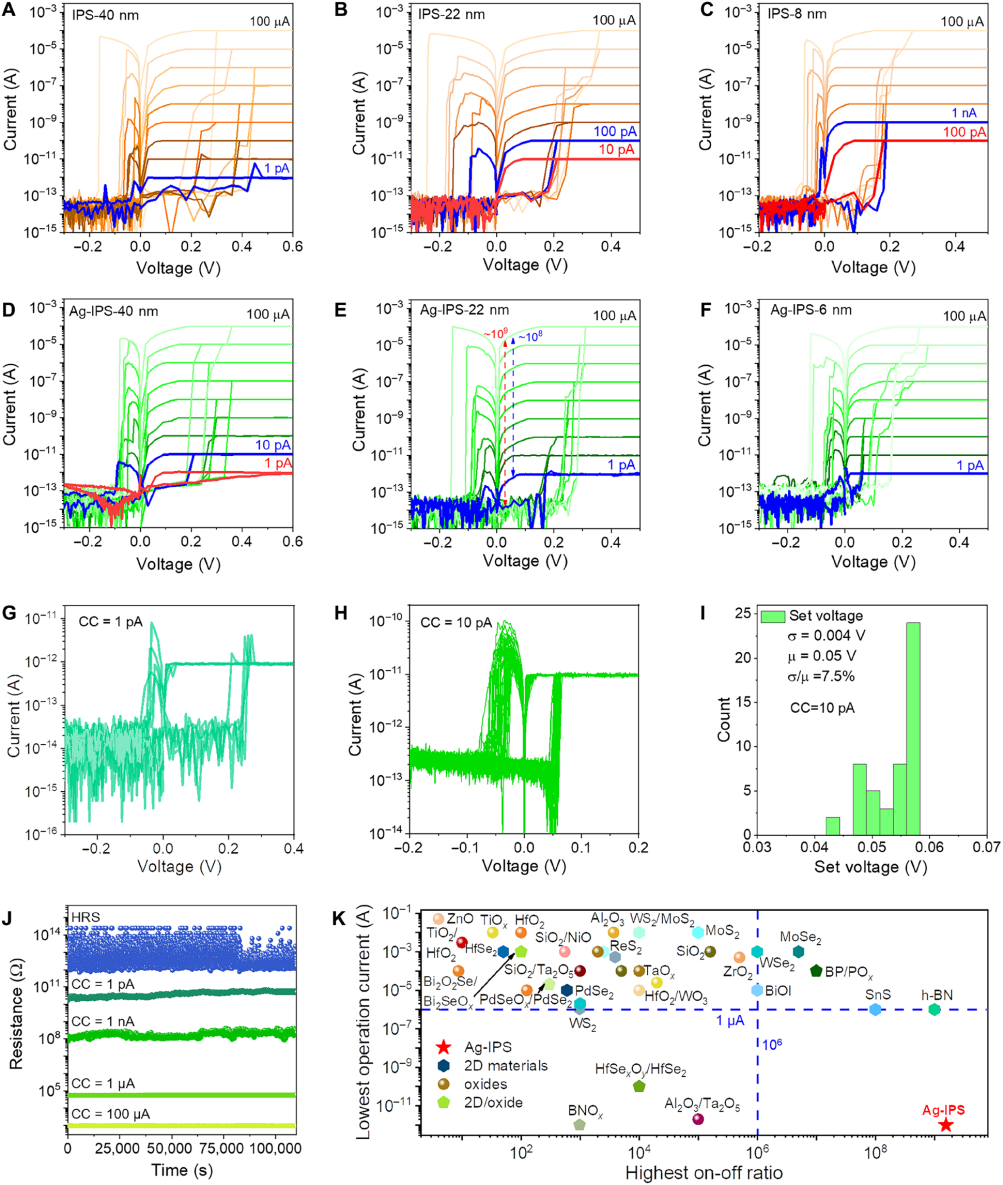
Ultralow operation current and ultrahigh on/off ratio in the Ag-IPS memristors. (**A** to **F**) Nonvolatile RS under different operation currents in (A to C) IPS memristors and (D to F) Ag-IPS memristors with different thicknesses. The red and blue curves mark the volatile RS and nonvolatile RS, respectively. All devices have a size of 2 × 2 μm^2^. (**G** to **I**) Repeated RS in Ag-IPS memristors at low operation currents of (G) 1 pA and (H) 10 pA, along with (I) the corresponding distribution of the operation voltage. (**J**) Retention property of the Ag-IPS memristor under different operation currents (read at 0.05 V). (**K**) Benchmark plot comparing the operation current and on/off ratio among the Ag-IPS, 2D material–based, and oxide-based memristors. Most reported memristors have microampere-level operation currents and on/off ratios below 10^6^. The Ag-IPS memristor stands out with the highest on/off ratio (>10^9^) and lowest operation current of 1 pA. For detailed references, please see tables S1 and S2 (marked in yellow).

The fast development of machine learning techniques and the rapid increase in scale of artificial neural networks place demands on the underlying electronic hardware, particularly their energy consumption (*2*). A memristor with a low operation current is essential to reduce energy consumption. The operation currents for nonvolatile RS of the thick IPS memristor (~40 nm) can reach an ultralow value of 1 pA. However, the low operation current cannot be maintained in its thin samples, and it will increase notably to 1 nA for a thickness of 8 nm, as shown in Fig. 2 (A to C). In contrast, the ultralow operation current can be achieved regardless of the thickness in Ag-IPS devices, where the scalability limit can be pushed to 6 nm with a 1-pA-level operation current (see Fig. 2, D to F). Moreover, the nonvolatile RS under low operation currents of 1 and 10 pA are reproductive and uniform, as shown in Fig. 2 (G and H). Meanwhile, the Ag-IPS memristor exhibits a good retention performance, lasting longer than 10^5^ s without obvious degradation under different operation currents at room temperature (Fig. 2J). With the exception of the operation current of 1 pA at 350 K, the resistance states also show good retention under low (250 K) and high temperatures (350 K), as presented in fig. S7. These results confirm the ability of Ag-IPS memristors to operate in the nonvolatile style under ultralow operation currents. The high HRS resistance and low operation current result in the on/off ratio and nonvolatile on/off ratio of up to 10^9^ and 10^8^, as marked in red and blue dashed lines, respectively, in Fig. 2E. Attributing to this wide resistance range, which is advantageous to accommodate a large number of resistance states, 32 states with small fluctuations are obtained using operation currents from 1 pA to 1 mA, as shown in fig. S8. The achievement of multiple resistance states with good retention is a key performance metric for building artificial neural networks with multi-states/weights for various applications.

Moreover, the thin Ag-IPS memristor (6 nm) provides an exceptionally low operation voltage of 0.05 V with a concentrated distribution of 7.5%, as presented in Fig. 2I. This is in contrast to the reported memristors that have a low operation voltage but a large variability or those with a small variation but a large operation voltage (*8*, *10*, *18*, *28*, *29*). Low operation voltages and operation currents are beneficial to low power consumption. As shown in fig. S9, the Ag-IPS memristor can be switched on either by a pulse train with a width of 200 ns and an amplitude of 1.3 V or by a single pulse with a width of 100 ns and an amplitude of 2.8 V (the pulse width is determined by the full width at half maximum), which is confirmed by the DC sweepings (fig. S9, B and E) and the retention tests (fig. S9, C and F). A low energy consumption of approximately 18.5 fJ per spike is achieved with a short switching time of 100 ns. A performance benchmarking in Fig. 2K shows that our Ag-IPS memristor with an operation current of 1 pA and an on/off ratio of 10^9^ occupies the uninhabited region. In summary, the Ag-IPS memristor has many outstanding metrics, including the nonvolatile operation current of 1 pA, maximal on-off ratio of 10^9^, sub–0.1 V operation voltage, femtojoule-level power consumption, and low variability of 2.3%. These values highlight that the Ag-IPS memristor is a promising candidate for high-efficiency AI hardware.

### RS mechanism of Ag-IPS memristor

**Fig. 3.**
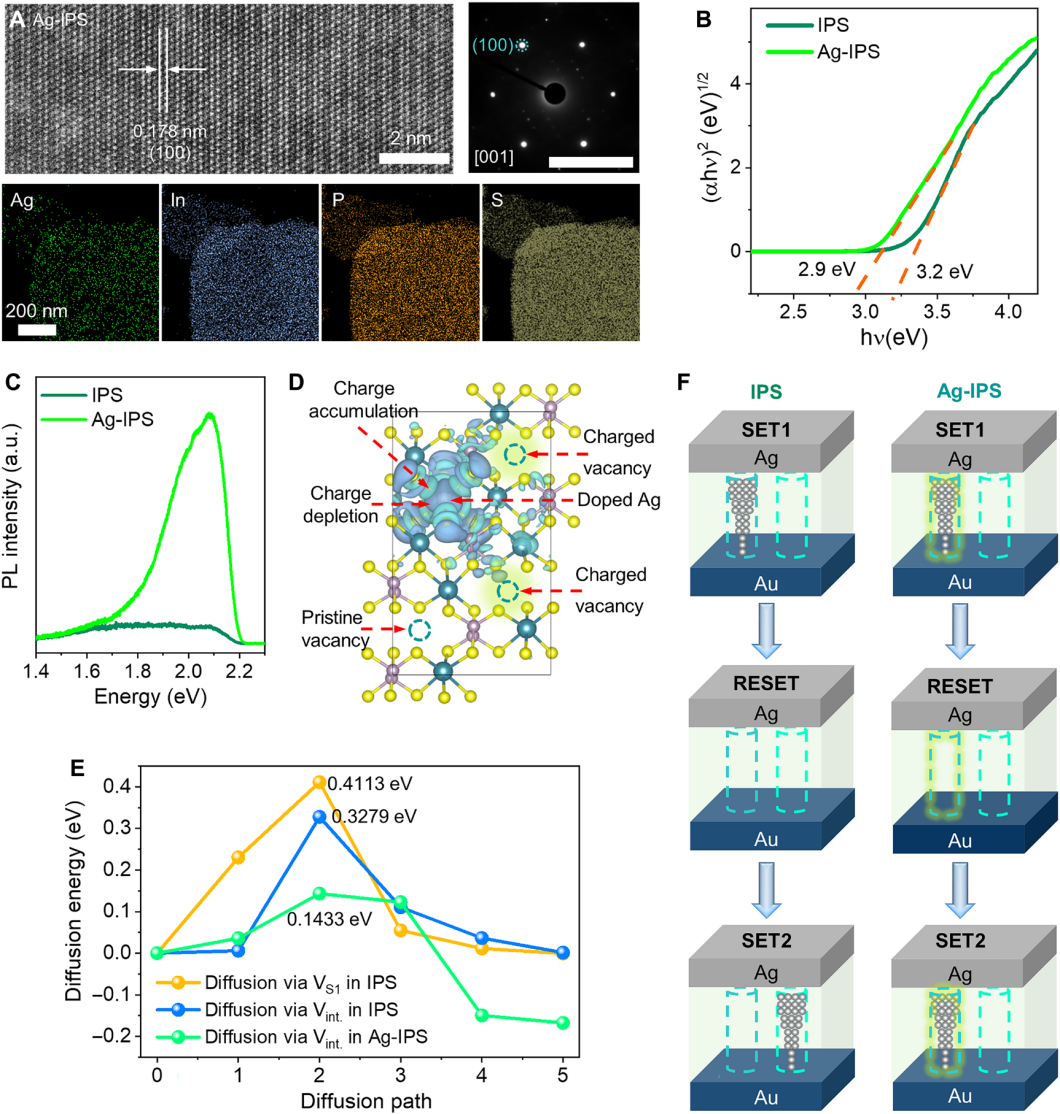
RS mechanism. (**A**) Top-view scanning STEM-ADF (top left) with the corresponding SAED image (top right) and EDS mapping (bottom) of Ag-IPS. The scale bar of the SAED image is 5 1/nm. (**B**) Micro–UV-visible absorption of the pristine IPS and Ag-IPS. The Ag-IPS has a smaller bandgap than the IPS. (**C**) Photoluminescence (PL) spectra of IPS and Ag-IPS under a 532-nm laser. (**D**) Differential charge density distribution of Ag-IPS. The differential charge density is calculated by subtracting the pristine IPS charge density from the Ag-IPS charge density. The lake blue and dusty blue isosurface contours represent the charge accumulation and depletion, respectively. (**E**) Calculated diffusion energy barriers of Ag migrating through adjacent layers via the structural vacancy (*V*_int._) and S vacancy (*V*_S1_) in the IPS and via charged vacancies around the doped Ag in Ag-IPS. (**F**) Schematic illustration of the concentrated CF formation in the Ag-IPS memristor.

The mechanism of the Ag doping–induced RS uniformity and low programming current are explored. The pristine exfoliated IPS, with an In:P:S ratio of 2:2.6:7.6, contains some P and S vacancies, as shown in fig. S10. The structure and composition of Ag-IPS are studied using the scanning transmission electron microscopy–annular dark field (STEM-ADF) and energy-dispersive x-ray spectroscopy (EDS) elemental mapping. The Ag-IPS nanosheet displays fine lattice fringes with the interplanar *d*-spacing of 0.178 nm and hexagonally arranged selected-area electron diffraction (SAED) spots recorded from the [001] zone axis (Fig. 3A, top), which inherits the structure of the single-crystalline IPS. A small amount of element Ag is detected in both EDS mapping and x-ray photoelectron spectroscopy (XPS) spectra in the Ag-IPS sample, as shown in Fig. 3A (bottom) and fig. S11, respectively. The atomic ratios of Ag:In:P:S calculated from the EDS and XPS data are 0.07:2:2.6:7.6 and 0.1:2:2.2:4.6, respectively, which confirms a low Ag doping and the existence of P and S vacancies. The doping is also evidenced by the ultraviolet (UV)–visible absorption spectra in Fig. 3B, where the measured bandgap is narrower than that of the pristine IPS. First-principles theory [density functional theory (DFT)] calculations reveal that Ag prefers to occupy the intrinsic structural vacancies in IPS since they show the lowest formation energy compared to the P and S vacancies (fig. S12). This behavior results in n-type doping, where the Ag doping contributes electron to the IPS and induces near-edge states that make the Fermi level enter the conduction band, as shown in fig. S13. The extra electrons donated by dopants can be verified by the photoluminescence enhancement effect, in which an obvious broad asymmetric peak is observed in the Ag-IPS sample (Fig. 3C) (*30*). Further calculation of the differential charge density distribution reveals the charge transport between the Ag atom and the surrounded atoms, where Ag donates electrons to the surrounded atoms, as shown in Fig. 3D. Thus, the vacancy site around the Ag dopant with nonequilibrium carriers becomes more charged than the vacancies farther from the doped Ag, as illustrated in Fig. 3D. In other words, the Ag doping forms a charged structural vacancy channel around Ag.

We confirm that the electrochemical metallization mechanism (ECM) is the dominant mechanism in the Ag-IPS memristor since obvious Ag migration and the formation of CFs are observed using cross-sectional high-resolution TEM (HR-TEM; fig. S14) and EDS analysis (fig. S15) in the Ag-IPS memristor. When inert Pd instead of Ag is used as the top electrode, no RS behavior is observed (fig. S16), which indicates that the Ag active ions from the electrode contribute to the formation of CFs instead of the dopant Ag. The structural vacancy channel should be the most desirable filament formation path since the diffusion barrier is lower than that via the S vacancy (see Fig. 3E). There are disordered P and especially S vacancies located beside the structural vacancy, which make the theoretical homogeneous vacancy channels inconsistent and result in variabilities of RS parameters in pristine IPS memristors. When Ag preoccupies the structural vacancy, electrons are donated and induce charged structural vacancies in the surroundings. The DFT calculation reveals that the diffusion barrier of Ag migrating through adjacent layers via the charged structural vacancy near the Ag dopant (0.1433 eV) is much lower than that via the pristine vacancies (0.3279 eV), which indicates a more energetic ion diffusion path around the doped Ag, as shown in Fig. 3E. Figure S17 illustrates the corresponding diffusion pathway. As a consequence, concentrated ion transport is achieved, and the filament formation path is confined around the doping Ag, as schematically illustrated in Fig. 3F, which results in an extremely uniform RS behavior in the Ag-IPS device. The post-formed Ag resulting from electrode oxidation differs from the pre-doped Ag, as it cannot induce a uniform RS behavior (note S1 and fig. S18). Meanwhile, the concentrated formation path can enhance the stability of the filament, leading to the achievement of an ultralow operation current in thin Ag-IPS samples, as discussed in note S2 and fig. S19. Moreover, the much lower diffusion barrier explains the lower operation voltages in the Ag-IPS memristor than those in the pristine IPS memristor. In addition, because of the low Ag doping, the HRS resistance of the Ag-IPS memristor shows only a limited decrease compared to its intrinsic sample. Therefore, the Ag-IPS memristor maintains a large resistance and on/off ratio (fig. S5 and note S2). In summary, the Ag-doped nonimperfection diffusion channel can provide an ultralow energy diffusion pathway that can confine the formation pathway and stabilize the filaments, which leads to a remarkably uniform RS switching with low operation current and operation voltage at the scalability limit.

### Experimental demonstration of logic-in-memory computing

**Fig. 4.**
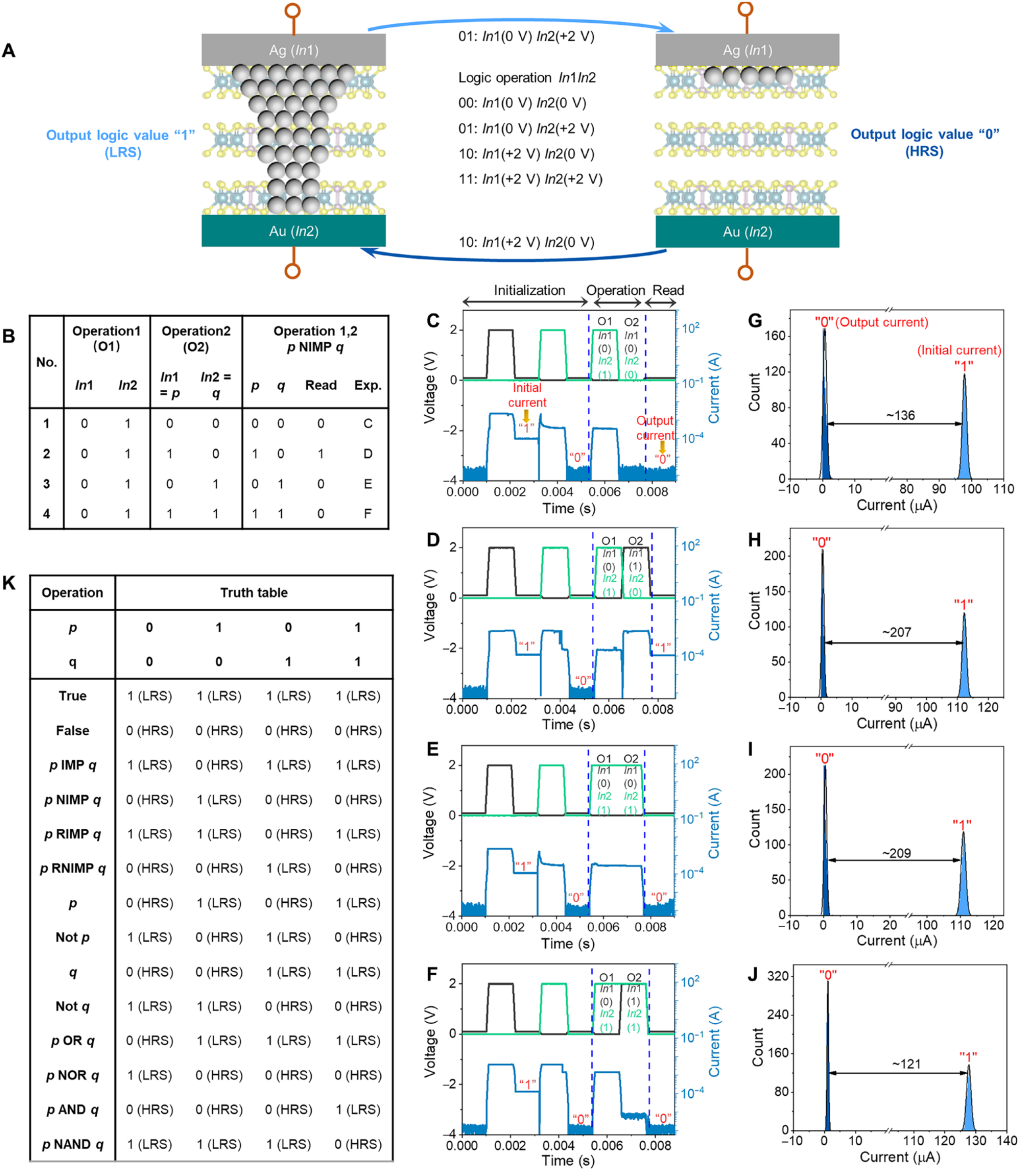
Experimental demonstration of Boolean logic operations in the Ag-IPS memristor. (**A**) Logic operations by a single device. The top (Ag) and bottom (Au) electrodes of the device are marked with *In*1 and *In*2, where logic operations *In*1*In*2 include 00, 01, 10, and 11. Here, 0 represents a low potential (0 V) and 1 represents a high potential (2 V). For example, 01 means applying 0 and 2 V to the terminals *In*1 and *In*2, respectively. (**B**) The truth tables show the operation sequence to the logic function *p* NIMP *q*. (**C** to **J**) Experimental demonstration of the *p* NIMP *q* logic operation and the corresponding distribution of the output current and initial current opposite to the output logic. For example, the current of state 0 in (G) is extracted from the output current after logic operations in (C), while the opposite logic, i.e., state 1, is obtained from the initialization step. The output currents of the four experiments are clearly distinguished from each opposite logic current. (**K**) The truth table of 14 fundamental Boolean logic functions was obtained from our experiments.

Memristors that can directly implement logic functions in a single device will reduce the hardware and materials footprint (*1*, *31*). Here, we demonstrate that the Ag-IPS memristor holds great potential for reliable Boolean logic computation. Through three steps (initialization, operation, and readout), any single device can conduct the required logic function (*31*). Logic terms such as logic variables *p* and *q*, terminals *In*1 and *In*2, and logic values “0” and “1” are introduced into the system. The logic variables *p* and *q* are assumed to be 0 or 1, which correspond to voltage levels applied to the junction terminals *In*1 and *In*2. The terminals *In*1 and *In*2 can be 0 or 1, where 0 represents the low potential and 1 represents the high potential. The top Ag electrode and bottom Au electrode are assigned to *In*1 and *In*2, respectively. The logic operation *In*1*In*2 includes 00, 01, 10, and 11. According to the logic operation, the voltage biases are simultaneously applied to the top and bottom electrodes. For example, when the combination of *In*1*In*2 is 01, the voltage biases of 0 and 2 V are applied to the top and bottom electrodes, respectively. The device in the HRS represents the state of 0, and the LRS represents the state of 1. Figure 4A shows the corresponding operation schematic.

The implementation of material nonimplication (NIMP) was taken as an example to verify the logic-in-memory ability of our device. The corresponding truth table of logic NIMP is shown in Fig. 4B. First, an initial characterization operation was performed before the logic operations to initialize the device to the HRS state and check whether the device could be correctly SET and RESET by the pulse bias voltages, as shown in the “initialization” regime of Fig. 4C. The logic operation can also be performed without initialization, as shown in fig. S22. However, the initialization step that can define states 0 and 1 is beneficial to quickly check the output state (0 or 1) after the logic operations and confirm the logic reliability by comparing the output current and initial current of the opposite logic. Subsequently, pulse bias voltage sequences designed according to the truth table (see Fig. 4B) were applied to the device to conduct the logic operation. Last, a readout operation (0.1 V) was performed to read the output current, i.e., the logic value. As shown in Fig. 4 (C to F), the states 0 and 1 were clearly distinguished and defined in the initialization step, and the output logic values were quickly confirmed according to the defined 0 and 1. Moreover, the ratio of the output logic current/opposite logic current is larger than 100 for all four logic experiments, which indicates the reliable logic computation of NIMP, as shown in Fig. 4 (G to J). Therefore, we have experimentally demonstrated the successful implementation of the NIMP logic function using eight pulse sequences in our device. By executing different pulse schemes, the other 13 fundamental Boolean logic functions in Fig. 4K, including material implication (IMP), Not, NOR, and NAND, can be experimentally realized using our device (see fig. S21). In addition, a simplified method can perform the logic operations, where only the top electrode is used to apply the voltage bias and the bottom electrode is grounded, as schematically shown in fig. S20B. Consequently, when the combinations of *In*1*In*2 are 00, 01, 10, and 11, the voltage biases of 0, −2, 2, and 0 V are applied to the top electrode, respectively. Figure S23 shows the corresponding operation results. The results indicate the potential of our device as logic-in-memory computing hardware.

### Convolutional image processing

**Fig. 5.**
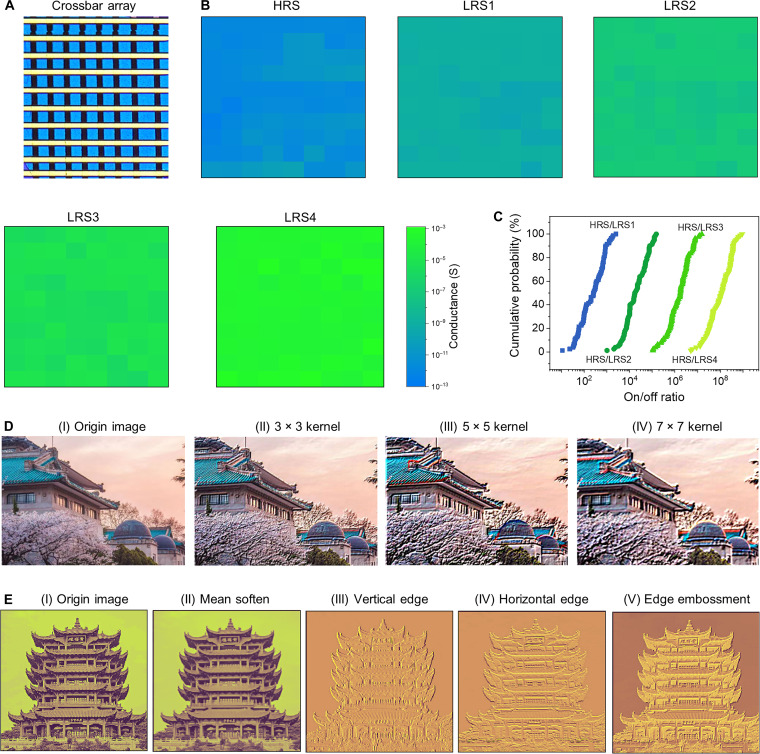
Convolutional image processing using the Ag-IPS memristor crossbar array. (**A**) Optical image of a 10 × 8 Ag-IPS memristor crossbar array; the device size is 2 × 2 μm^2^. (**B**) The conductance distributions correspond to the five states (HRS and LRS1 to LRS4). The four LRS states are obtained using different operation currents of 100 pA, 10 nA, 1 μA, and 100 μA. The conductance data are extracted from the *I-V* curves in fig. S24. (**C**) Cumulative probability plots of the on/off ratio in different LRS states over 80 devices in the Ag-IPS crossbar array. (**D**) Comparison of the convolutional image processing of edge embossment with different kennel sizes. The 10 × 8 Ag-IPS memristor crossbar array can implement 3 × 3, 5 × 5, and 7 × 7 kernels for different image processing applications. (**E**) Parallel convolutional image processing of four feature maps implemented by the Ag-IPS memristor crossbar array.

The Ag-IPS memristor crossbar array with high yield was further fabricated. Figure 5A shows a 10 × 8 memristor crossbar array, where all devices present nonvolatile RS under operation currents from 100 pA to 100 μA (see fig. S24). Five states (HRS, LRS1, LRS2, LRS3, and LRS4) using operation currents of 100 pA, 10 nA, 1 μA, and 100 μA show good spatial uniformity, as shown in Fig. 5B. These states can be reliably distinguished without overlap, and the on/off ratios of HRS/LRS1 to LRS4 are at least 10, 10^3^, 10^5^, and 5 × 10^7^ for each operation current, respectively, as shown in fig. S25 and Fig. 5C.

Convolutional neural networks (CNNs) have been one of the most important models for image recognition, where image processing (or feature extraction) is the basic and most frequently used operation. Convolutional kernels in different sizes can extract different feature information. The small-size kernel can extract the local texture feature information, and the large-size kernel can extract the global contour feature information (*32*). Using or combining different kernels with varied sizes can obtain different image processing effects for various applications. Here, by leveraging the Ag-IPS memristor crossbar array structure and the corresponding measured conductance (*G*) data in Fig. 5B, we constructed convolutional kernels with different sizes. Specifically, two memristors were used as a differential pair to represent both positive and negative weight values. Then, the weights of −1/+1 were mapped by the HRS and LRS, i.e., *G*^HRS^ − *G*^LRS1/2/3/4^ ~ −1, and *G*^LRS1/2/3/4^ − *G*^HRS^ ~ +1. Consequently, two 3 × 3 (or 5 × 5 or 7 × 7) crossbar arrays were used to construct the target 3 × 3 (or 5 × 5 or 7 × 7) *G* convolution kernel (*33*), where the conductance values were obtained from the conductance map (Fig. 5B) according to the position and resistive state (HRS, LRS1, LRS2, LRS3, or LRS4) of the memristor, as illustrated in fig. S26. The building of Wuhan University (WHU building) was used as the input image, and the image intensity was converted into voltages, as illustrated in fig. S26A (*16*). Subsequently, the convolution of the input voltage matrix and *G* convolution kernel was implemented, i.e., *I*_out_ = *V_ij_* * *G_ij_*. Here, *I*_out_ is the sum of differential currents, i.e., $I_{\text{out}}=\sum \left(I_{j}^{+}-I_{j+3}^{-}\right)$ . Last, *I*_out_ was converted into the pixel. The complete image processing was achieved by conducting the convolution operations over the entire image. *G* convolution kernels were mapped from the conductance of the actual devices in the crossbar array, whereas the convolutional operation was simulated using the software code. Figure 5D and fig. S27 show the image processing of embossment and mean softening using varied convolutional kernels, respectively. As displayed in Fig. 5D, the smaller convolution kernel pays more attention to the details of the image (local texture), e.g., both thick and thin branches are highlighted when a 3 × 3 kernel is used. When the size of the convolution kernel increases, the global contour feature of the WHU building is enhanced with some loss in local texture (thin branches become unobvious). Similarly, the softening effect is enhanced when the kernel size increases, as displayed in fig. S27. Therefore, the convolutional kernels with varied sizes were successfully constructed using our Ag-IPS memristor array, and the impact of kernel sizes on image processing is well presented.

In the above image processing scheme, the voltages should be applied column by column. A more efficient parallel convolutional image processing is further demonstrated using the Ag-IPS memristor crossbar array, as shown in Fig. 5E and fig. S28. Here, the 3 × 3 input voltage matrix was unrolled into one column, i.e., 9 × 1. A two-column array of 9 × 2 instead of 3 × 3 × 2 was used to implement the 3 × 3 convolutional kernel, where one column represents the sum of the products of the positive values in the filter (*I*^+^), and the other column represents the sum of the products of the negative values in the filter (*I*^−^) (*33*). Consequently, a differential readout of the two-column currents (*I*^+^ − *I*^−^) is the convolution output. Using the entire 9 × 8 size of the array, four different *G* convolution kernels for vertical and horizontal edge extraction, edge embossment, and mean soften were simultaneously built. Similarly, the *G* convolutional kernels were constructed using the measured conductance data of the memristors, and fig. S28A shows the conductance map that corresponds to the target convolution kernel. The product and summation of *V_i_* and *G_ij_*, i.e., $I_{j}^{+}$ or $I_{j}^{-}$ = Σ*V_i_* × *G_ij_*, the sum of differential currents representing the convolutional output, i.e., $I_{\text{outj}}=I_{j}^{+}-I_{j}^{-}$ , and the final operation of converting *I*_out_ into image pixel were performed using the software code. With this strategy, four different image processing actions can be simultaneously conducted, as illustrated in Fig. 5E and fig. S28A. The yellow crane tower was used as the input image. The image was successfully softened after the convolution operation, where the pixel distribution was similar to the original one but slightly adjusted [Fig. 5E (II) and fig. S28B]. The vertical and horizontal edges of the yellow crane tower were extracted, where the pixel intensity was concentrated in a narrow range, as presented in Fig. 5E (III and IV) and fig. S28 (C and D). Meanwhile, the relief effect of the yellow crane tower was realized by edge embossment processing with a shrunken pixel distribution, as exhibited in Fig. 5E (V) and fig. S28E. These results indicate that the parallel processing of four feature maps was achieved. In summary, the Ag-IPS memristor crossbar array can implement convolution kernels with varied sizes for different image processing actions, which manifests its potential as the building block for CNNs.

## DISCUSSION

In this study, we demonstrated that using Ag-doped inherent vacancy channel of IPS can facilitate a low-energy ion diffusion pathway to confine the filament formation. Attributed to the confinement, the Ag-IPS memristor achieves ultralow variabilities of RS parameters at extreme values. At a low operation voltage of 0.2 V, the variation is as low as 3.8%. Similarly, the high resistance at 10^11^ ohms exhibits a variation down to 2.3%, and a large on/off ratio of 10^8^ shows a small variation of 6.9%. Moreover, the ultralow operation current of 1 pA, femtojoule-level switching energy, and high-yield memristor crossbar array are also obtained. On the basis of the developed memristors, the 14 Boolean logic functions are experimentally implemented. Leveraging its crossbar array with high yield and uniform states, convolution kernels with varied sizes and parallel convolution operations for multiple image processing tasks are successfully performed. We provide an unconventional strategy for superior in-memory computing hardware, which can combine low variabilities with excellent RS parameters.

## MATERIALS AND METHODS

### Device fabrication

The IPS and Ag-doped IPS crystals were synthesized by chemical vapor transport by Six Carbon Technologies Inc. The bottom electrode by depositing a layer of Cr/Au (5/20 nm) onto a 300-nm silicon dioxide substrate is first fabricated through an e-beam-thermal evaporator system. Then, the IPS or Ag-doped IPS nanosheets with different thicknesses were exfoliated and transferred onto the bottom electrode. Last, the devices were finished by depositing an Ag/Au (40/40 nm) as the top electrode. Both the top and bottom electrodes were patterned by UV lithography using AZ5214 as the photoresist. The size of both IPS and Ag-IPS devices is 2 × 2 μm^2^.

### Device measurement

A B1500 semiconductor parameter analyzer equipped with a probe station (Lakeshore, TTP4) is used to measure the *I*-*V* characteristics of the memristors. The Ag electrode was used as the top electrode to apply bias voltage, and the Au electrode was used as the bottom electrode and grounded. The pulse measurement is performed using an SCS-4200 semiconductor parameter analyzer equipped with a probe station (Lakeshore, TTP4).

### Material characterization

The top-view atomic resolution STEM-ADF and EDS elemental mapping were conducted in JEM-ARM200CF (80 kV). The cross-sectional HR-TEM was performed in JEM-NEOARM (200 kV). X-ray photoelectron spectra were measured using a Quantera PHI II system with a monochromated Al anode. The thicknesses of the Ag-doped IPS and IPS nanosheets were measured by atomic force microscope (Bruker). UV-visible absorption spectra were measured by the MStarter ABS deep UV-near infrared micro-absorption spectroscopy test system.

### Calculation method

All first-principles calculations were performed within the Vienna Ab Initio Simulation Package (VASP) based on DFT (*34*). The projector augmented wave (PAW) potentials are used to deal with the electronic exchange-correlation interaction along with the GGA functional in the parameterization of the Perdew, Burke, and Ernzerhof (PBE) pseudopotential (*35*, *36*). A plane wave representation for the wave function with a cutoff energy of 450 eV is applied. Geometry optimizations are performed using a conjugate gradient minimization until all the forces acting on the ions are less than 0.01 eV/Å per atom. In the calculations, the 2 × 2 × 2 *k*-point mesh is adopted (*37*). The climbing image nudged elastic band (CI-NEB) method is adopted to calculate diffusion barriers of Ag in In_4/3_P_2_S_6_ and Ag-doped In_4/3_P_2_S_6_ (*38*, *39*). The DFT-D3 method is used to describe the van der Waals interaction (*40*). The formation energies of Ag occupying different vacancies are calculated by the following formula:

$$E_{\text{formation}}=E_{\text{occupied}}-E_{\text{pure}}-\mu_{\text{Ag}}+\mu_{\text{occupied}}$$

where *E*_occupied_ and *E*_pure_ are the total energies of Ag-doped In_4/3_P_2_S_6_ and pure In_4/3_P_2_S_6_, respectively. μ_Ag_ or μ_occupied_ is the corresponding chemical potential of Ag, S, or P atoms.

## Supplementary Materials

### This PDF file includes:

Notes S1 to S4 Figs. S1 to S28 Tables S1 and S2 References

## References

[1] N. J. Tye, S. Hofmann, P. Stanley-Marbell, Materials and devices as solutions to computational problems in machine learning. Nat. Electron. 6, 479–490 (2023).

[2] AI hardware has an energy problem. Nat. Electron. 6, 463 (2023).

[3] M. Zhao, B. Gao, J. Tang, H. Qian, H. Wu, Reliability of analog resistive switching memory for neuromorphic computing. Appl. Phys. Rev. 7, 011301 (2020).

[4] M. Lanza, A. Sebastian, W. D. Lu, M. Le Gallo, M.-F. Chang, D. Akinwande, F. M. Puglisi, H. N. Alshareef, M. Liu, J. B. Roldan, Memristive technologies for data storage, computation, encryption, and radio-frequency communication. Science 376, eabj9979 (2022). 35653464 10.1126/science.abj9979

[5] M. Li, H. Liu, R. Zhao, F.-S. Yang, M. Chen, Y. Zhuo, C. Zhou, H. Wang, Y.-F. Lin, J. J. Yang, Imperfection-enabled memristive switching in van der Waals materials. Nat. Electron. 6, 491–505 (2023).

[6] M. Lanza, R. Waser, D. Ielmini, J. J. Yang, L. Goux, J. Suñe, A. J. Kenyon, A. Mehonic, S. Spiga, V. Rana, S. Wiefels, S. Menzel, I. Valov, M. A. Villena, E. Miranda, X. Jing, F. Campabadal, M. B. Gonzalez, F. Aguirre, F. Palumbo, K. Zhu, J. B. Roldan, F. M. Puglisi, L. Larcher, T.-H. Hou, T. Prodromakis, Y. Yang, P. Huang, T. Wan, Y. Chai, K. L. Pey, N. Raghavan, S. Dueñas, T. Wang, Q. Xia, S. Pazos, Standards for the characterization of endurance in resistive switching devices. ACS Nano 15, 17214–17231 (2021). 34730935 10.1021/acsnano.1c06980

[7] Y. Shen, W. Zheng, K. Zhu, Y. Xiao, C. Wen, Y. Liu, X. Jing, M. Lanza, Variability and yield in h-BN-based memristive circuits: The role of each type of defect. Adv. Mater. 33, e2103656 (2021). 34480775 10.1002/adma.202103656

[8] X. Huang, K. A. Jiang, Y. Niu, R. Wang, D. Zheng, A. Dong, X. Dong, C. Mei, J. Lu, S. Liu, Z. Gan, N. Zhong, H. Wang, Configurable ultra-low operating voltage resistive switching between bipolar and threshold behaviors for Ag/TaO*_x_*/Pt structures. Appl. Phys. Lett. 113, 112103 (2018).

[9] P. Lei, H. Duan, L. Qin, X. Wei, R. Tao, Z. Wang, F. Guo, M. Song, W. Jie, J. Hao, High-performance memristor based on 2D layered BiOI nanosheet for low-power artificial optoelectronic synapses. Adv. Funct. Mater. 32, 2201276 (2022).

[10] S. Chen, M. R. Mahmoodi, Y. Shi, C. Mahata, B. Yuan, X. Liang, C. Wen, F. Hui, D. Akinwande, D. B. Strukov, M. Lanza, Wafer-scale integration of two-dimensional materials in high-density memristive crossbar arrays for artificial neural networks. Nat. Electron. 3, 638–645 (2020).

[11] H. Zhao, Z. P. Dong, H. Tian, D. DiMarzi, M. G. Han, L. H. Zhang, X. D. Yan, F. X. Liu, L. Shen, S.-J. Han, S. Cronin, W. Wu, J. Tice, J. Guo, H. Wang, Atomically thin femtojoule memristive device. Adv. Mater. 29, 1703232 (2017). 10.1002/adma.201703232 29067743

[12] X. H. Wu, R. J. Ge, P. A. Chen, H. Chou, Z. P. Zhang, Y. F. Zhang, S. Banerjee, M.-H. Chiang, J. C. Lee, D. Akinwande, Thinnest nonvolatile memory based on monolayer h-BN. Adv. Mater. 31, e1806790 (2019). 30773734 10.1002/adma.201806790

[13] S. Roy, G. Niu, Q. Wang, Y. Wang, Y. Zhang, H. Wu, S. Zhai, P. Shi, S. Song, Z. Song, Z.-G. Ye, C. Wenger, T. Schroeder, Y.-H. Xie, X. Meng, W. Luo, W. Ren, Toward a reliable synaptic simulation using Al-doped HfO_2_ RRAM. ACS Appl. Mater. Interfaces 12, 10648–10656 (2020). 32043352 10.1021/acsami.9b21530

[14] Y. Wang, M. Cao, J. Bian, Q. Li, J. Su, Flexible ZnO nanosheet-based artificial synapses prepared by low-temperature process for high recognition accuracy neuromorphic computing. Adv. Funct. Mater. 32, 2209907 (2022).

[15] J. Park, E. Park, S. Kim, H. Y. Yu, Nitrogen-induced enhancement of synaptic weight reliability in titanium oxide-based resistive artificial synapse and demonstration of the reliability effect on the neuromorphic system. ACS Appl. Mater. Interfaces 11, 32178–32185 (2019). 31392881 10.1021/acsami.9b11319

[16] Y. Li, S. Chen, Z. Yu, S. Li, Y. Xiong, M.-E. Pam, Y.-W. Zhang, K.-W. Ang, In-memory computing using memristor arrays with ultrathin 2D PdSeO_x_/PdSe_2_ heterostructure. Adv. Mater. 34, e2201488 (2022). 35393702 10.1002/adma.202201488

[17] T. Wang, S. Brivio, E. Cianci, C. Wiemer, M. Perego, S. Spiga, M. Lanza, Improving HfO_2_-based resistive switching devices by inserting a TaO*_x_* thin film via engineered in situ oxidation. ACS Appl. Mater. Interfaces 14, 24565–24574 (2022). 35585656 10.1021/acsami.2c03364

[18] R. Zhang, H. Huang, Q. Xia, C. Ye, X. Wei, J. Wang, L. Zhang, L. Q. Zhu, Role of oxygen vacancies at the TiO_2_/HfO_2_ interface in flexible oxide-based resistive switching memory. Adv. Electron. Mater. 5, 1800833 (2019).

[19] Y.-L. Zhu, K.-H. Xue, X.-M. Cheng, C. Qiao, J.-H. Yuan, L.-H. Li, X.-S. Miao, Uniform and robust TiN/HfO_2_/Pt memristor through interfacial Al-doping engineering. Appl. Surf. Sci. 550, 149274 (2021).

[20] S. Brivio, J. Frascaroli, S. Spiga, Role of Al doping in the filament disruption in HfO_2_ resistance switches. Nanotechnology 28, 395202 (2017). 28718452 10.1088/1361-6528/aa8013

[21] Z. J. Tan, V. Somjit, C. Toparli, B. Yildiz, N. Fang, Electronegative metal dopants improve switching variability in Al_2_ O_3_ resistive switching devices. Phys. Rev. Mater. 6, 105002 (2022).

[22] M. A. Susner, M. Chyasnavichyus, M. A. McGuire, P. Ganesh, P. Maksymovych, Metal thio- and selenophosphates as multifunctional van der Waals layered materials. Adv. Mater. 29, 1602852 (2017). 10.1002/adma.201602852 28833546

[23] F. M. Wang, T. A. Shifa, P. Yu, P. He, Y. Liu, F. Wang, Z. X. Wang, X. Y. Zhan, X. D. Lou, F. Xia, J. He, New frontiers on van der Waals layered metal phosphorous trichalcogenides. Adv. Funct. Mater. 28, 1802151 (2018).

[24] H. Zhou, J. Zhou, S. Wang, P. Li, Q. Li, J. Xue, Z. Zhou, R. Wang, Y. Yu, Y. Weng, F. Zheng, Z. Li, S. Ju, L. Fang, L. You, Size effect on optical and vibrational properties of van der Waals layered In_4/3_P_2_S_6_. APL Mater. 10, 061111 (2022).

[25] A. Krishnaprasad, D. Dev, S. S. Han, Y. Shen, H.-S. Chung, T.-S. Bae, C. Yoo, Y. Jung, M. Lanza, T. Roy, MoS_2_ synapses with ultra-low variability and their implementation in boolean logic. ACS Nano 16, 2866–2876 (2022). 35143159 10.1021/acsnano.1c09904

[26] M. Ismail, C. Mahata, O. Kwon, S. Kim, Neuromorphic synapses with high switching uniformity and multilevel memory storage enabled through a Hf-Al-O alloy for artificial intelligence. ACS Appl. Electron. Mater. 4, 1288–1300 (2022).

[27] F. Wan, Q. Wang, T. Harumoto, T. Gao, K. Ando, Y. Nakamura, J. Shi, Truly electroforming-free memristor based on TiO_2_-CoO phase-separated oxides with extremely high uniformity and low power consumption. Adv. Funct. Mater. 30, 2007101 (2020).

[28] K. Wang, L. Li, R. Zhao, J. Zhao, Z. Zhou, J. Wang, H. Wang, B. Tang, C. Lu, J. Lou, J. Chen, X. Yan, A pure 2H-MoS_2_ nanosheet-based memristor with low power consumption and linear multilevel storage for artificial synapse emulator. Adv. Electron. Mater. 6, 1901342 (2020).

[29] Z. Ma, J. Ge, W. Chen, X. Cao, S. Diao, Z. Liu, S. Pan, Reliable memristor based on ultrathin native silicon oxide. ACS Appl. Mater. Interfaces 14, 21207–21216 (2022). 35476399 10.1021/acsami.2c03266

[30] J.-S. Yao, J. Ge, B.-N. Han, K.-H. Wang, H.-B. Yao, H.-L. Yu, J.-H. Li, B.-S. Zhu, J.-Z. Song, C. Chen, Q. Zhang, H.-B. Zeng, Y. Luo, S.-H. Yu, Ce^3+^-doping to modulate photoluminescence kinetics for efficient CsPbBr_3_ nanocrystals based light-emitting diodes. J. Am. Chem. Soc. 140, 3626–3634 (2018). 29341604 10.1021/jacs.7b11955

[31] J. Li, S. Hou, Y.-R. Yao, C. Zhang, Q. Wu, H.-C. Wang, H. Zhang, X. Liu, C. Tang, M. Wei, W. Xu, Y. Wang, J. Zheng, Z. Pan, L. Kang, J. Liu, J. Shi, Y. Yang, C. J. Lambert, S.-Y. Xie, W. Hong, Room-temperature logic-in-memory operations in single-metallofullerene devices. Nat. Mater. 21, 917–923 (2022). 35835820 10.1038/s41563-022-01309-y

[32] J. Q. Ai, Y. X. Mao, Q. W. Luo, L. Jia, M. D. Xing, SAR target classification using the multikernel-size feature fusion-based convolutional neural network. IEEE Trans. Geosci. Remote Sens. 60, 5214313 (2022).

[33] C. Li, M. Hu, Y. Li, H. Jiang, N. Ge, E. Montgomery, J. Zhang, W. Song, N. Dávila, C. E. Graves, Z. Li, J. P. Strachan, P. Lin, Z. Wang, M. Barnell, Q. Wu, R. S. Williams, J. J. Yang, Q. Xia, Analogue signal and image processing with large memristor crossbars. Nat. Electron. 1, 52–59 (2018).

[34] G. Kresse, J. Hafner, Ab initio molecular-dynamics simulation of the liquid-metal-amorphous-semiconductor transition in germanium. Phys. Rev. B Condens. Matter 49, 14251–14269 (1994). 10010505 10.1103/physrevb.49.14251

[35] P. E. Blöchl, Projector augmented-wave method. Phys. Rev. B Condens. Matter 50, 17953–17979 (1994). 9976227 10.1103/physrevb.50.17953

[36] B. Hammer, L. B. Hansen, J. K. Norskov, Improved adsorption energetics within density-functional theory using revised Perdew-Burke-Ernzerhof functionals. Phys. Rev. B 59, 7413–7421 (1999).

[37] G. Kresse, D. Joubert, From ultrasoft pseudopotentials to the projector augmented-wave method. Phys. Rev. B 59, 1758–1775 (1999).

[38] G. Henkelman, H. Jonsson, Improved tangent estimate in the nudged elastic band method for finding minimum energy paths and saddle points. J. Chem. Phys. 113, 9978–9985 (2000).

[39] D. Sheppard, R. Terrell, G. Henkelman, Optimization methods for finding minimum energy paths. J. Chem. Phys. 128, 134106 (2008). 18397052 10.1063/1.2841941

[40] S. Grimme, J. Antony, S. Ehrlich, H. Krieg, A consistent and accurate ab initio parametrization of density functional dispersion correction (DFT-D) for the 94 elements H-Pu. J. Chem. Phys. 132, 154104 (2010). 20423165 10.1063/1.3382344

[41] Y. Li, L. Loh, S. Li, L. Chen, B. Li, M. Bosman, K.-W. Ang, Anomalous resistive switching in memristors based on two-dimensional palladium diselenide using heterophase grain boundaries. Nat. Electron. 4, 348–356 (2021).

[42] S. Li, M.-E. Pam, Y. Li, L. Chen, Y.-C. Chien, X. Fong, D. Chi, K.-W. Ang, Wafer-scale 2D hafnium diselenide based memristor crossbar array for energy-efficient neural network hardware. Adv. Mater. 34, e2103376 (2021). 34510567 10.1002/adma.202103376

[43] M. E. Pam, S. Li, T. Su, Y.-C. Chien, Y. Li, Y. S. Ang, K.-W. Ang, Interface-modulated resistive switching in Mo-irradiated ReS_2_ for neuromorphic computing. Adv. Mater. 34, e2202722 (2022). 35610176 10.1002/adma.202202722

[44] X. F. Lu, Y. Zhang, N. Wang, S. Luo, K. Peng, L. Wang, H. Chen, W. Gao, X. H. Chen, Y. Bao, G. Liang, K. P. Loh, Exploring low power and ultrafast memristor on p-type van der Waals SnS. Nano Lett. 21, 8800–8807 (2021). 34644096 10.1021/acs.nanolett.1c03169

[45] X. Yan, Q. Zhao, A. P. Chen, J. Zhao, Z. Zhou, J. Wang, H. Wang, L. Zhang, X. Li, Z. Xiao, K. Wang, C. Qin, G. Wang, Y. Pei, H. Li, D. Ren, J. Chen, Q. Liu, Vacancy-induced synaptic behavior in 2D WS_2_ nanosheet–based memristor for low-power neuromorphic computing. Small 15, e1901423 (2019). 31045332 10.1002/smll.201901423

[46] M. Sivan, Y. Li, H. Veluri, Y. Zhao, B. Tang, X. Wang, E. Zamburg, J. F. Leong, J. X. Niu, U. Chand, A. V.-Y. Thean, All WSe_2_ 1T1R resistive RAM cell for future monolithic 3D embedded memory integration. Nat. Commun. 10, 5201 (2019). 31729375 10.1038/s41467-019-13176-4 PMC6858359

[47] X. Yan, C. Qin, C. Lu, J. Zhao, R. Zhao, D. Ren, Z. Zhou, H. Wang, J. Wang, L. Zhang, X. Li, Y. Pei, G. Wang, Q. Zhao, K. Wang, Z. Xiao, H. Li, Robust Ag/ZrO_2_/WS_2_/Pt memristor for neuromorphic computing. ACS Appl. Mater. Interfaces 11, 48029–48038 (2019). 31789034 10.1021/acsami.9b17160

[48] R. Xu, H. Jang, M.-H. Lee, D. Amanov, Y. Cho, H. Kim, S. Park, H.-J. Shin, D. Ham, Vertical MoS_2_ double-layer memristor with electrochemical metallization as an atomic-scale synapse with switching thresholds approaching 100 mV. Nano Lett. 19, 2411–2417 (2019). 30896171 10.1021/acs.nanolett.8b05140

[49] R. J. Ge, X. H. Wu, M. Kim, J. P. Shi, S. Sonde, L. Tao, Y. F. Zhang, J. C. Lee, D. Akinwande, Atomristor: Nonvolatile resistance switching in atomic sheets of transition metal dichalcogenides. Nano Lett. 18, 434–441 (2018). 29236504 10.1021/acs.nanolett.7b04342

[50] B. Tang, H. Veluri, Y. Li, Z. G. Yu, M. Waqar, J. F. Leong, M. Sivan, E. Zamburg, Y.-W. Zhang, J. Wang, A. V. Y. Thean, Wafer-scale solution-processed 2D material analog resistive memory array for memory-based computing. Nat. Commun. 13, 3037 (2022). 35650181 10.1038/s41467-022-30519-w PMC9160094

[51] H. Jeong, J. Kim, D. Y. Kim, J. Kim, S. Moon, O. F. Ngome Okello, S. Lee, H. Hwang, S.-Y. Choi, J. K. Kim, Resistive switching in few-layer hexagonal boron nitride mediated by defects and interfacial charge transfer. ACS Appl. Mater. Interfaces 12, 46288–46295 (2020). 32959644 10.1021/acsami.0c12012

[52] Y. Shi, X. Liang, B. Yuan, V. Chen, H. Li, F. Hui, Z. Yu, F. Yuan, E. Pop, H. S. P. Wong, M. Lanza, Electronic synapses made of layered two-dimensional materials. Nat. Electron. 1, 458–465 (2018).

[53] W. Zhang, H. Gao, C. Deng, T. Lv, S. Hu, H. Wu, S. Xue, Y. Tao, L. Deng, W. Xiong, An ultrathin memristor based on a two-dimensional WS_2_/MoS_2_ heterojunction. Nanoscale 13, 11497–11504 (2021). 34165120 10.1039/d1nr01683k

[54] T. Ahmed, S. Kuriakose, S. A. Tawfik, E. L. H. Mayes, A. Mazumder, S. Balendhran, M. J. S. Spencer, D. Akinwande, M. Bhaskaran, S. Sriram, S. Walia, Mixed ionic-electronic charge transport in layered black-phosphorus for low-power memory. Adv. Funct. Mater. 32, 2107068 (2022).

[55] L. Liu, Y. Li, X. D. Huang, J. Chen, Z. Yang, K.-H. Xue, M. Xu, H. W. Chen, P. Zhou, X. S. Miao, Low-power memristive logic device enabled by controllable oxidation of 2D HfSe_2_ for in-memory computing. Adv. Sci. 8, e2005038 (2021). 10.1002/advs.202005038 PMC8336485 34050639

[56] Y. Xia, J. Wang, R. Chen, H. Wang, H. Xu, C. Jiang, W. Li, X. Xiao, 2D heterostructure of Bi_2_O_2_Se/Bi_2_SeO*_x_* nanosheet for resistive random access memory. Adv. Electron. Mater. 8, 2200126 (2022).

[57] V. K. Sahu, A. K. Das, R. S. Ajimsha, P. Misra, Low power high speed 3-bit multilevel resistive switching in TiO_2_ thin film using oxidisable electrode. J. Phys. D Appl. Phys. 53, 225303 (2020).

[58] Q. Liu, S. Gao, Y. Li, W. Yue, C. Zhang, H. Kan, G. Shen, HfO_2_/WO_3_ heterojunction structured memristor for high-density storage and neuromorphic computing. Adv. Mater. Technol. 8, 2201143 (2022).

[59] Q. Xue, Y. Peng, L. Cao, Y. Xia, J. Liang, C.-C. Chen, M. Li, T. Hang, Ultralow set voltage and enhanced switching reliability for resistive random-access memory enabled by an electrodeposited nanocone array. ACS Appl. Mater. Interfaces 14, 25710–25721 (2022). 35604125 10.1021/acsami.2c03978

[60] S. G. Ren, R. Ni, X. D. Huang, Y. Li, K. H. Xue, X. S. Miao, Pt/Al_2_O_3_/TaO*_X_*/ Ta self-rectifying memristor with record-low operation current (< 2 pA), low power (fJ), and high scalability. IEEE Trans. Electron Devices 69, 838–842 (2022).

[61] B. K. You, J. M. Kim, D. J. Joe, K. Yang, Y. Shin, Y. S. Jung, K. J. Lee, Reliable memristive switching memory devices enabled by densely packed silver nanocone arrays as electric-field concentrators. ACS Nano 10, 9478–9488 (2016). 27718554 10.1021/acsnano.6b04578

[62] B. K. You, W. I. Park, J. M. Kim, K.-I. Park, H. K. Seo, J. Y. Lee, Y. S. Jung, K. J. Lee, Reliable control of filament formation in resistive memories by self-assembled nanoinsulators derived from a block copolymer. ACS Nano 8, 9492–9502 (2014). 25192434 10.1021/nn503713f

[63] S. Petzold, A. Zintler, R. Eilhardt, E. Piros, N. Kaiser, S. U. Sharath, T. Vogel, M. Major, K. P. McKenna, L. Molina-Luna, L. Alff, Forming-free grain boundary engineered hafnium oxide resistive random access memory devices. Adv. Electron. Mater. 5, 1900484 (2019).

[64] E. Shahrabi, C. Giovinazzo, M. Hadad, T. LaGrange, M. Ramos, C. Ricciardi, Y. Leblebici, Switching kinetics control of W-based reram cells in transient operation by interface engineering. Adv. Electron. Mater. 5, 1800835 (2019).

[65] J. Wang, L. Li, H. Huyan, X. Pan, S. S. Nonnenmann, Highly uniform resistive switching in HfO_2_ films embedded with ordered metal nanoisland arrays. Adv. Funct. Mater. 29, 1808430 (2019).

[66] D. S. Kuzmichev, Y. Y. Lebedinskii, C. S. Hwang, A. M. Markeev, Atomic layer deposited oxygen-deficient TaO*_x_* layers for electroforming-free and reliable resistance switching memory. Phys. Status Solidi Rapid Res. Lett. 12, 1800429 (2018).

[67] X. Guo, Q. Wang, X. Lv, H. Yang, K. Sun, D. Yang, H. Zhang, T. Hasegawa, D. He, SiO_2_/Ta_2_O_5_ heterojunction ECM memristors: Physical nature of their low voltage operation with high stability and uniformity. Nanoscale 12, 4320–4327 (2020). 32043511 10.1039/c9nr09845c

[68] C.-Y. Zhu, J.-K. Qin, P.-Y. Huang, H.-L. Sun, N.-F. Sun, Y.-L. Shi, L. Zhen, C.-Y. Xu, 2D indium phosphorus sulfide (In_2_P_3_S_9_): An emerging van der Waals high-k dielectrics. Small 18, e2104401 (2022). 34825486 10.1002/smll.202104401

[69] Z. Wang, S. Joshi, S. E. Savel'ev, H. Jiang, R. Midya, P. Lin, M. Hu, N. Ge, J. P. Strachan, Z. Li, Q. Wu, M. Barne, G. L. Li, H. L. Xin, R. S. Williams, Q. Xia, J. J. Yang, Memristors with diffusive dynamics as synaptic emulators for neuromorphic computing. Nat. Mater. 16, 101–108 (2017). 27669052 10.1038/nmat4756

[70] Z. Wang, M. Rao, R. Midya, S. Joshi, H. Jiang, P. Lin, W. Song, S. Asapu, Y. Zhuo, C. Li, H. Wu, Q. Xia, J. J. Yang, Threshold switching of Ag or Cu in dielectrics: Materials, mechanism, and applications. Adv. Funct. Mater. 28, 1704862 (2018).

[71] J.-H. Cha, S. Y. Yang, J. Oh, S. Choi, S. Park, B. C. Jang, W. Ahn, S.-Y. Choi, Conductive-bridging random-access memories for emerging neuromorphic computing. Nanoscale 12, 14339–14368 (2020). 32373884 10.1039/d0nr01671c

