# Supplementary material for: Ag-doped non–imperfection-enabled uniform memristive neuromorphic device based on van der Waals indium phosphorus sulfide
Source: Sci Adv. 2024 Mar 13;10(11):eadk9474. doi: 10.1126/sciadv.adk9474 (PMC10936950; doi:10.1126/sciadv.adk9474)
Supplement: Supplementary file 1 — Notes S1 to S4 Figs. S1 to S28 Tables S1 and S2 References [file sciadv.adk9474_sm.pdf]

Science Advances
AAAS

Supplementary Materials for

Ag-doped non–imperfection-enabled uniform memristive neuromorphic device based on van der Waals indium phosphorus sulfide

Yesheng Li *et al.*

Corresponding author: Yesheng Li, lys2021@whu.edu.cn; Jun He, he-jun@whu.edu.cn

Sci. Adv. **10**, eadk9474 (2024)
DOI: 10.1126/sciadv.adk9474

This PDF file includes:

Notes S1 to S4
Figs. S1 to S28
Tables S1 and S2
References

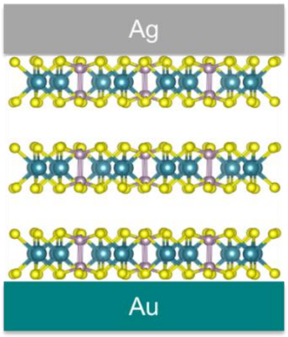

Fig. S1. Schematic showing the structure of the IPS or Ag-IPS memristor. The vertical sandwich-like memristors were fabricated by using IPS or Ag-IPS as the resistive layer, and Ag and Au as the top active and bottom inert electrodes, respectively.

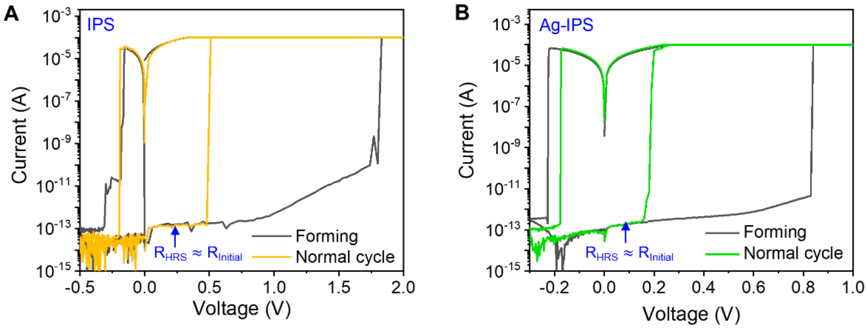

Fig. S2. Forming process in the (A) IPS and (B) Ag-IPS memristors. A forming process is needed before the normal operation in the IPS and Ag-IPS memristors. The forming voltages are about 1.8 V and 0.8 V for IPS and Ag-IPS memristors, respectively. Both the IPS and Ag-IPS memristors show similar HRS resistance with each initial resistance. It indicates that there are no or very few residual Ag atoms after the RESET operation, thus the memristors preserve the high HRS resistance.

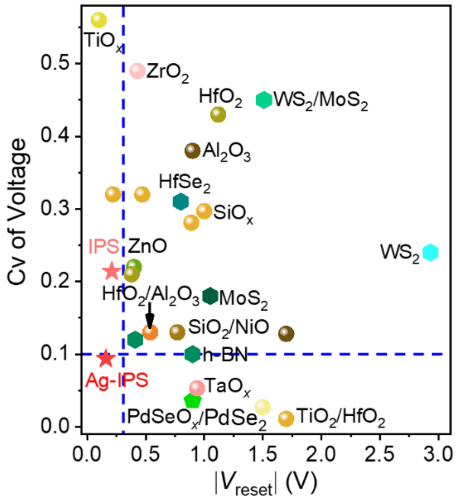

Fig. S3. Variation comparison of reset voltage among IPS, Ag-IPS, and reported 2D-material- and oxide-based memristors. C_V: coefficient of variation, $C_V = \sigma/\mu$, where σ and μ are the standard deviation and mean value of each parameter, respectively. For detailed references please see Tables S1 and S2 (marked in orange).

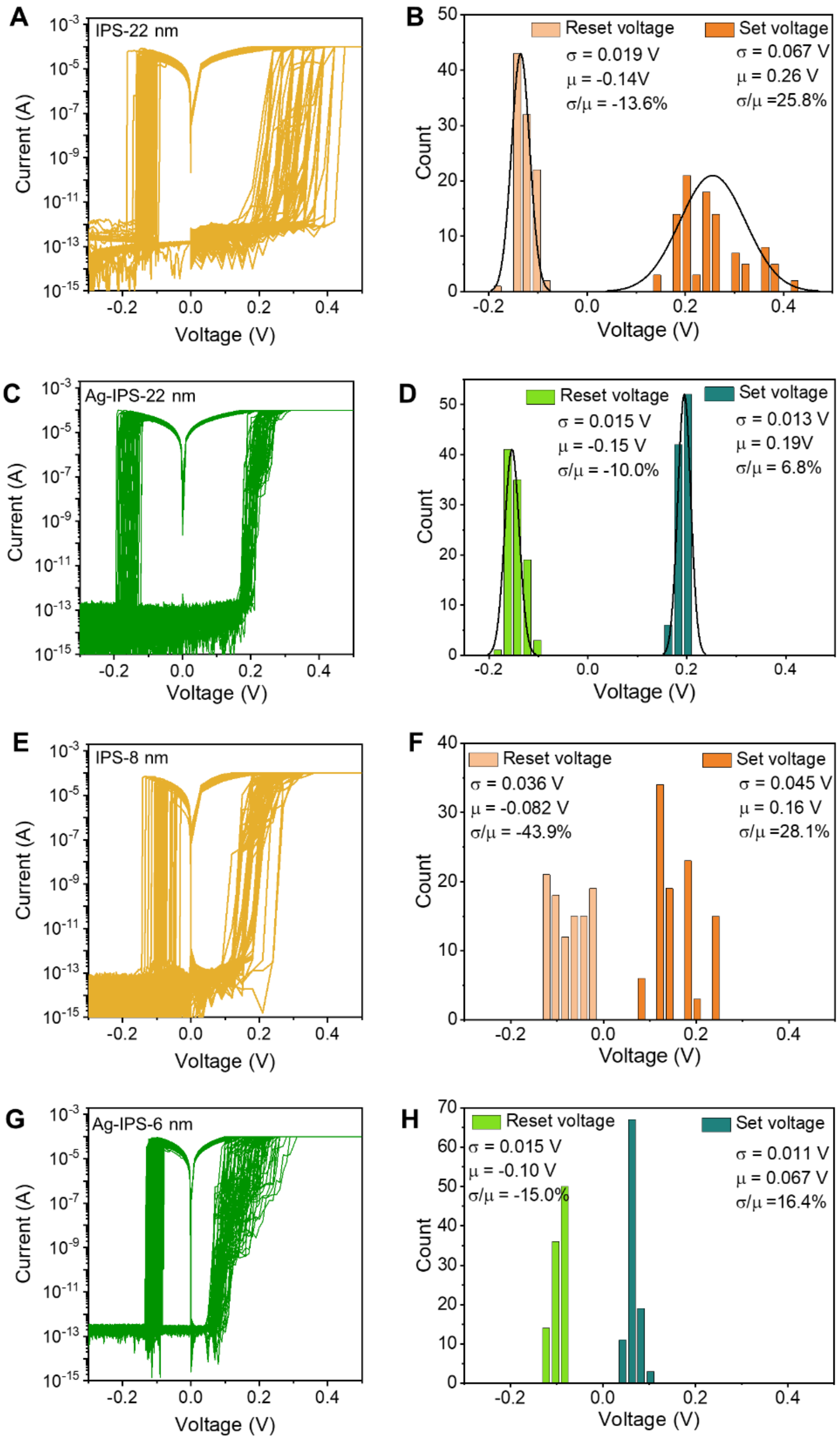

Fig. S4. Improved uniformity in the thin Ag-IPS memristors. *I*-*V* curves during 100 cycles and the corresponding histogram distribution of the set and reset voltages: (**A, B**) IPS (~22 nm), (**C, D**) Ag-IPS (~22 nm), (**E, F**) IPS (~8 nm), and (**G, H**) Ag-IPS (~6 nm).

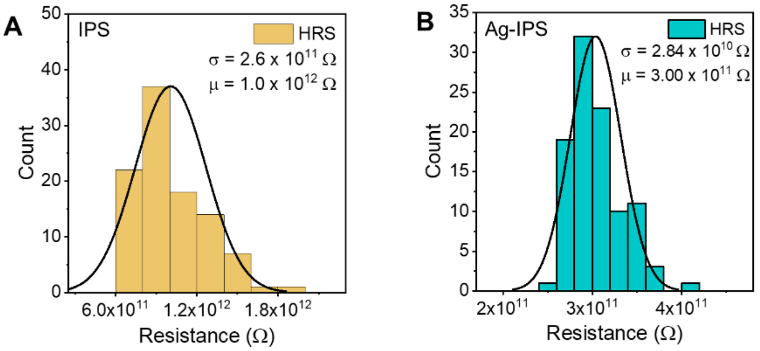

Fig. S5. Histograms of the HRS resistance distribution. (**A**) IPS (~40 nm), (**B**) Ag-IPS (~40 nm). The data are extracted from the *I-V* curves of Figs. 1B and G in the main manuscript.

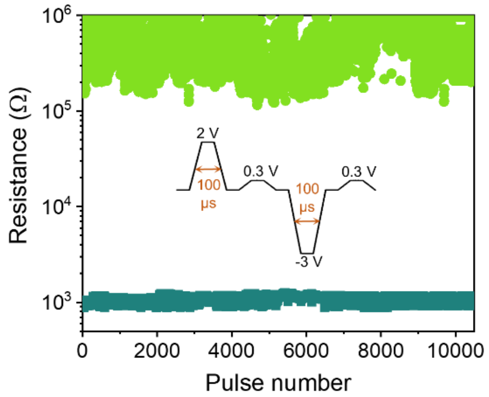

Fig. S6. Endurance-cycling data of the Ag-IPS memristor using voltage pulses. The inset is the applied pulse waveform, which consists of a 2 V/100 µs set pulse and a -3 V/100 µs reset pulse. Each set and reset pulse follows a read pulse. High endurance switching is achieved for 10^4 cycles. It is noted that the noise would be obvious when measuring the low current (due to the equipment factor), leading to the variation of HRS resistance. However, the LRS resistance shows good uniformity with a C_V of 7.3%.

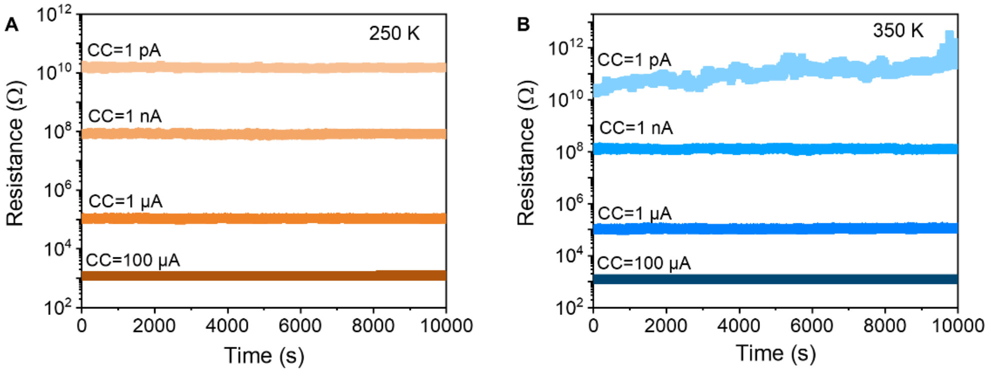

Fig. S7. Retention properties of the Ag-IPS memristor at different temperatures (read at 0.05 V). (**A**) Retention test at 250 K, (**B**) retention test at 350 K. At a low temperature of 250 K, all the resistance states under different operation currents (1 pA to 100 µA) display good retention without obvious degradation (Fig. S7A). When the temperature rises to 350 K, the resistance states corresponding to 1 nA to 100 µA, still shows good retention. The resistance gradually degrades with time at 350 K for 1 pA (Fig. S7B).

Table S1 Comparison among the IPS memristor, Ag-IPS memistor and 2D materials-based memrisors.

No.	Device	Material	V_{set} (V)	V_{reset} (V)	C_V of V_{set}	C_V of V_{reset}	HRS (ohm)	LRS (ohm)	C_V of HRS	C_V of LRS	On/off ratio	Operation current	Reference
1.	**Ag/Ag-IPS (40 nm)/Au**	**2DM**	**0.21**	**-0.16**	**3.80%**	**9.40%**	**3.00E+11**	**2.19E+03**	**5.80%**	**2.30%**	**1.30E+08**	**10 pA**	**This work**
2.	**Ag/IPS (40 nm)/Au**	**2DM**	**0.43**	**-0.21**	**20.70%**	**21.40%**	**1.00E+12**	**3.05E+03**	**26.0%**	**8.20%**	**3.00E+08**	**1 pA**	**This work**
**3.**	Ti/irradiated $PdSe_2$/Au	2DM	0.6	N	7%	N	4.40E+05	7.70E+02	N	N	5.50E+02	10 µA	(*41*)
4.	**Ti/$HfSe_2$/Au**	**2DM**	**0.7**	**-0.8**	**18.10%**	**31.20%**	**1.00E+05**	**1.00E+03**	**N**	**N**	**5.00E+01**	**1 mA**	**(*42*)**
**5.**	Ti/ReS_2/Au 9 min	2DM	1.09	3	22%	N	1.00E+07	4.00E+03	N	N	2.50E+03	1 mA	(*43*)
**6.**	Ti/ReS_2/Au 12 min	2DM	1.55	3	15.90%	N	6.00E+07	4.00E+03	N	N	1.50E+04	1 mA	(*43*)
**7.**	Ti/ReS_2/Au 15 min	2DM	1.24	3	33%	N	3.00E+06	8.00E+03	N	N	3.75E+02	1 mA	(*43*)
**8.**	Ag/SnS/Au	2DM	0.2	-0.2	5.50%	N	3.00E+10	1.00E+04	N	N	1.00E+08	1 µA	(*44*)
**9.**	Ag/BiOI/Pt	2DM	0.05	-0.05	N	N	1.00E+09	1.00E+04	N	N	1.00E+06	10 µA	(*9*)
**10.**	Pd/WS_2/Pt	2DM	0.6	-0.2	N	N	1.00E+08	1.00E+05	N	N	1.00E+03	1 µA	(*45*)
**11.**	Ag/WSe_2/Ag	2DM	0.5	0.3	N	N	2.00E+07	2.00E+04	N	N	1.00E+03	2 µA	(*46*)
12.	**Ag/WS_2/Pt**	**2DM**	**3.12**	**–2.93**	**22.10%**	**23.90%**	**8.00E+03**	**2.00E+06**	**N**	**N**	**4.00E+03**	**0.5 mA**	**(*47*)**
**13.**	Cu/MoS_2/Au	2DM	0.25	-0.15	N	N	5.00E+02	1.25E+02	N	N	4.00E+00	2 mA	(*48*)
**14.**	Au/MoS_2/Au	2DM	1	-1.25	N	N	2.50E+06	2.50E+02	N	N	1.00E+04	10 mA	(*49*)
**15.**	Au/$MoSe_2$/Au	2DM	3	-1	N	N	1.25E+09	2.50E+02	N	N	5.00E+06	1 mA	(*49*)
**16.**	Au/WSe_2/Au	2DM	2	-0.7	N	N	2.50E+08	2.50E+02	N	N	1.00E+06	1 mA	(*49*)
17.	**Ti/MoS_2/Au**	**2DM**	**1.08**	**-1.05**	**19.40%**	**18.10%**	**2.00E+04**	**8.00E+02**	**N**	**N**	**2.50E+01**	**1 mA**	**(*50*)**
18.	**Ti/h-BN/Au**	**2DM**	**0.66**	**–0.41**	**22.70%**	**12.20%**	**6.00E+04**	**4.00E+02**	**N**	**N**	**1.50E+02**	**2 mA**	**(*51*)**
**19.**	Au/h-BN/Au	2DM	2.7	–0.9	2.02%	9.61%	1.00E+11	1.00E+02	N	N	1.00E+09	1 µA	(*10*)
**20.**	Ti/h-BN/Au	2DM	1.8	–0.8	N	N	4.20E+04	1.00E+02	N	N	4.20E+02	1 mA	(*52*)
**21.**	Al/WS_2/MoS_2/ITO	2DM	1.5	-1.8	21.00%	45.00%	6.00E+06	6.00E+02	N	N	1.00E+04	10 mA	(*53*)
**22.**	ITO/BP/PO_x/Ag	oxide/2DM	0.39	-0.37	N	N	1.00E+10	2.00E+02	N	N	1.00E+07	100 µA	(*54*)
**23.**	Ti/$PdSeO_x$/$PdSe_2$/Au	oxide/2DM	0.7	–0.9	4.80%	3.60%	3.00E+05	1.30E+03	N	N	3.00E+02	20 µA	(*16*)
**24.**	Ti/$HfSe_xO_y$/$HfSe_2$/Au	oxide/2DM	2.3	-0.7	N	N	1.00E+11	1.00E+07	N	N	1.00E+04	100 pA	(*55*)
**25.**	Au/Bi_2O_2Se/Bi_2SeO_x/Au	oxide/2DM	2	-1	N	1	1.00E+04	1.25E+02	N	N	1.00E+02	1 mA	(*56*)
**26.**	Ag/BNO_x/MLG	2DMO	0.63	–0.6	14.00%	N	1.00E+13	2.00E+10	N	N	5.00E+02	0.9 pA	(*11*)

Table S2 Comparison among the IPS memristor, Ag-IPS memistor and oxides-based memristors.

No.	Device	Material	V_{set} (V)	V_{reset} (V)	C_V of V_{set}	C_V of V_{reset}	HRS (ohm)	LRS (ohm)	C_V of HRS	C_V of LRS	On/off ratio	Operation current	Reference
1.	**Ag/Ag-IPS (40 nm)/Au**	**2DM**	**0.21**	**-0.16**	**3.80%**	**9.40%**	**3.00E+11**	**2.19E+03**	**5.80%**	**2.30%**	**1.30E+08**	**10 pA**	**This work**
2.	**Ag/IPS (40 nm)/Au**	**2DM**	**0.43**	**-0.21**	**20.70%**	**21.40%**	**1.0E+12**	**3.05E+03**	**26.0%**	**8.20%**	**3.00E+08**	**1 pA**	**This work**
**3.**	Cu/TiO_2/Pt	oxide	0.2	-0.1	18%	56%	1.00E+08	5.00E+03	N	32%	2.00E+04	25 μA	(*57*)
**4.**	Ag/SiO_x/P-Si	oxide	3	-1	24.60%	29.70%	1.00E+08	1.00E+04	N	N	1.00E+02	1 mA	(*29*)
**5.**	Au-Ag/SiO_x/P-Si	oxide	3.3	-1.7	3.90%	1.10%	1.00E+07	1.00E+04	N	N	1.00E+04	10 mA	(*29*)
**6.**	Ti/TiO_x:N/Pt	oxide	1	-1	6.21%	N	1.00E+03	9.00E+01	5.95%	2.76%	1.10E+01	10 mA	(*15*)
7.	**Ti/TiO_x/Pt**	**oxide**	**0.7**	**-0.6**	**13.38%**	**N**	**2.00E+03**	**6.00E+01**	**29.13%**	**6.34%**	**3.30E+01**	**10 mA**	**(*15*)**
**8.**	Ag/ZnO/Pt	oxide	0.4	-0.4	20%	22%	3.30E+02	9.00E+01	N	N	4.00E+00	50 mA	(*14*)
**9.**	Si/HfO_2/WO_3/Ag	oxide	3	-2.5	N	N	3.00E+08	1.00E+04	N	N	1.00E+04	10 μA	(*58*)
**10.**	Ag/Al_2O_3/Pt	oxide	-1.3	0.9	65.90%	38.20%	4.30E+06	6.52E+01	71.30%	28.60%	6.60E+04	10 mA	(*59*)
11.	**Ni/Ag/Al_2O_3/Pt**	**oxide**	**-0.4**	**1.7**	**16.80%**	**12.80%**	**3.10E+05**	**8.25E+01**	**51.20%**	**14.20%**	**3.70E+03**	**10 mA**	**(*59*)**
**12.**	Pt/Al_2O_3/TaO_x/Ta	oxide	8	-4	N	N	1.00E+14	1.00E+11	N	N	1.00E+03	2 pA	(*60*)
**13.**	Ag/SiO_2/Ag	oxide	0.27	–0.22	28.00%	32.20%	1.00E+05	1.00E+02	N	N	1.00E+03	1 mA	(*61*)
**14.**	Ag/SiO_2/Si	oxide	1.11	–0.47	51.50%	31.80%	1.00E+05	1.00E+02	N	N	1.00E+03	1 mA	(*61*)
**15.**	Ti/SiO_2/NiO/Ni	oxide	1.6	-0.77	11.30%	13.00%	1.10E+05	2.00E+02	50%	N	5.50E+02	1 mA	(*62*)
16.	**Ti/SiO_2/Ni**	**oxide**	**2.3**	**-0.89**	**34.30%**	**28.10%**	**3.20E+07**	**2.00E+02**	**197%**	**N**	**1.60E+05**	**1 mA**	**(*62*)**
**17.**	TiN/HfO_2/Pt	oxide	–1.31	1.54	11.50%	17.50%	1.00E+04	1.00E+02	N	N	1.00E+02	10 mA	(*63*)
**18.**	Pt/HfO_2/Ti/W	oxide	0.88	–0.38	12.50%	21.00%	2.10E+05	6.30E+02	N	N	3.36E+02	1 mA	(*64*)
19.	**Ti/HfO_2/Pt**	**oxide**	**1.3**	**–1.12**	**27.70%**	**42.80%**	**2.30E+05**	**2.60E+04**	**99.10%**	**85.10%**	**8.70E+00**	**100 μA**	**(*65*)**
**20.**	Ti/HfO_2/PtD30/HfO_2/Pt	oxide	0.95	–0.81	23.10%	3.70%	1.00E+05	1.35E+03	11%	5.30%	6.80E+02	100 μA	(*65*)
**21.**	Ti/HfO_2/TiD30/HfO_2/Pt	oxide	0.94	–0.81	14.90%	21.00%	1.60E+05	1.20E+04	93.90%	83.00%	1.40E+01	100 μA	(*65*)
**22.**	Ti/HfO_2/AgD30/HfO_2/Pt	oxide	0.4	–0.41	30.00%	21.90%	2.00E+04	7.50E+02	25.00%	38.00%	1.20E+02	100 μA	(*65*)
**23.**	Ti/HfO_2/PtD90/HfO_2/Pt	oxide	0.74	–0.53	32.40%	17.00%	1.20E+04	9.40E+02	20.90%	26.90%	5.90E+02	100 μA	(*65*)
**24.**	Ti/HfO_2/TiD90/HfO_2/Pt	oxide	0.85	–0.74	17.60%	28.40%	3.90E+04	1.39E+04	44.00%	50.40%	6.30E+01	100 μA	(*65*)
**25.**	Ti/HfO_2/AgD90/HfO_2/Pt	oxide	0.37	–0.37	35.10%	32.40%	5.20E+04	8.10E+03	32.20%	43.80%	2.00E+02	100 μA	(*65*)
**26.**	Ag/ZrO_2/Pt	oxide	0.64	–0.43	34.40%	48.80%	1.00E+09	2.00E+03	N	N	5.00E+05	0.5 mA	(*47*)

**27.**	Pt/0% doped Al HfO_2/TiN	oxide	–1	0.78	9.30%	9.30%	5.62E+04	3.66E+03	56.95%	123.84%	1.54E+01	1 mA	(*13*)
**28.**	Pt/9.8% doped Al HfO_2/TiN	oxide	–1.58	0.69	18.80%	9.80%	3.01E+05	5.57E+02	28.42%	68.05%	5.41E+02	1 mA	(*13*)
**29.**	Pt/16.5% doped Al HfO_2/TiN	oxide	–1.64	0.67	16.30%	6.70%	5.47E+05	4.94E+02	32.33%	86.47%	1.11E+03	1 mA	(*13*)
**30.**	Pt/31.8% doped Al HfO_2/TiN	oxide	–1.34	0.85	16.30%	26.00%	1.22E+05	8.25E+02	54.72%	70.28%	1.48E+02	1 mA	(*13*)
**31.**	Pt/46.2% doped Al HfO_2/TiN	oxide	–1.15	1.08	10.90%	15.80%	1.12E+05	1.15E+03	98.09%	139.47%	9.73E+01	1 mA	(*13*)
**32.**	Ta/TaO_x/Pt	oxide	–0.55	0.94	10.90%	5.30%	2.59E+05	1.24E+02	N	N	2.00E+03	1 mA	(*66*)
**33.**	Pt/HfO_2/Al_2O_3/W	oxide/oxide	1.43	–0.54	18.90%	12.90%	9.20E+04	3.00E+02	N	N	3.00E+02	1 mA	(*64*)
34.	**ITO/TiO_2/HfO_2/Pt**	**oxide/oxide**	**1.8**	**–1.5**	**2.80%**	**2.70%**	**3.00E+03**	**3.00E+02**	**3.20%**	**3.00%**	**1.00E+01**	**3 mA**	**(*18*)**
**35.**	Ag/SiO_2/Ta_2O_5/Pt	oxide/oxide	0.2	-0.1	N	N	1.00E+06	1.00E+03	N	N	1.00E+03	100 µA	(*67*)
**36.**	Ag/SiO_2/Pt	oxide	0.8	-0.1	N	N	5.00E+06	1.00E+03	N	N	5.00E+03	100 µA	(*67*)
**37.**	Ag/Ta_2O_5/Pt	oxide	0.3	-0.1	N	N	1.00E+07	1.00E+03	N	N	1.00E+04	100 µA	(*67*)

Note for Table S1 and Table S2: “2DM” means “two-dimension material”, “2DMO” means “oxidized two-dimension material”. The table cells marked in blue are the data sources of Fig. 1D for variation comparison of set voltage; the table cells marked in orange are the data sources of Fig. S3 for variation comparison of reset voltage; the table cells marked in green are the data sources of Fig. 1J for variation comparison of HRS resistance; the table cells marked in yellow are the data sources of Fig. 2K for comparison of on/off and operation current; the rows marked in purple in Table S1 and Table S2 are data sources of Figs. 1K and L, respectively.

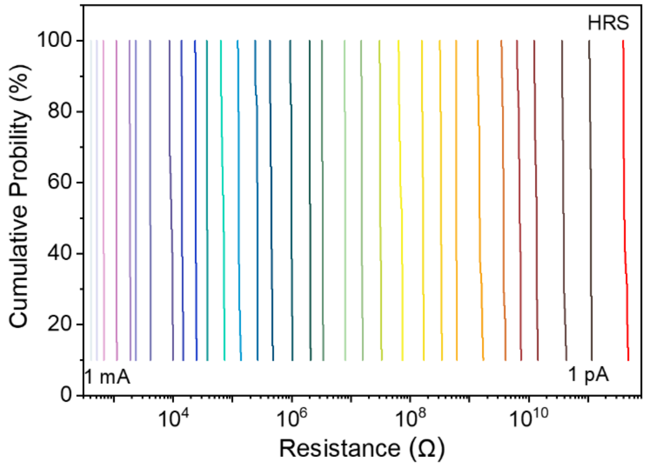

Fig. S8. 32 states realized by varied operation currents in the Ag-IPS memristor. The resistances are read at 0.05 V.

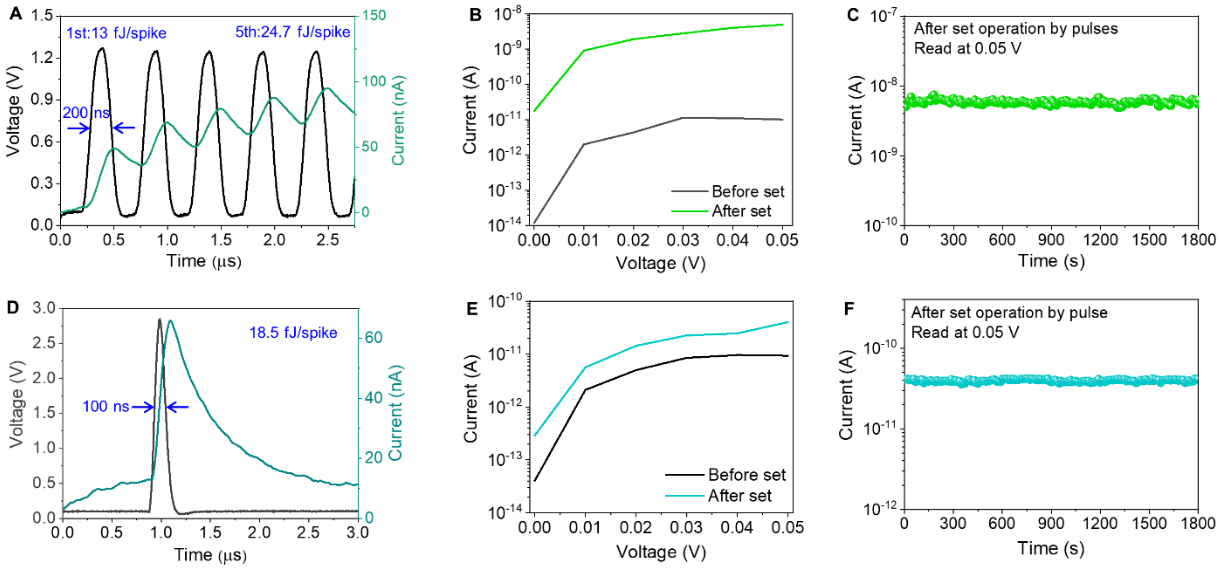

Fig. S9. Switching on the Ag-IPS memristor by pulses. (**A-C**) using a pulse train with an amplitude of 1.3 V and a width of 200 ns, (B) the DC currents of the memristor before and after switching on, (C) current retention after switching on; (**D-F**) using single pulse with an amplitude of 2.8 V and a width of 100 ns, (F) the DC currents of the memristor before and after switching on, (E) current retention after switching on. It is noted that the pulse width is determined by the full width at half-maximum of the voltage pulse. DC currents and retention tests after the pulse operation are used to further check whether the device has been switched on. The current becomes higher than that in the initial state (Figs. S9B and E) and can be retained at least 1800 s (Figs. S9C and F), i.e., from HRS transforms to LRS. It indicates that the device can be switched on either by a low amplitude of 1.3 V or a short switching time of 100 ns, in which both fJ-level energy consumptions are achieved. Especially, a low energy consumption of ~18.5 fJ/spike is obtained with a short switching time of 100 ns. The power consumption calculation (E = pulse amplitude × current × pulse width): 1.3 V × 50 nA × 200 ns = 13 fJ/spike, 1.3 V × 95 nA × 200 ns = 24.7 fJ/spike, 2.8 V × 66 nA × 100 ns = 18.5 fJ/spike.

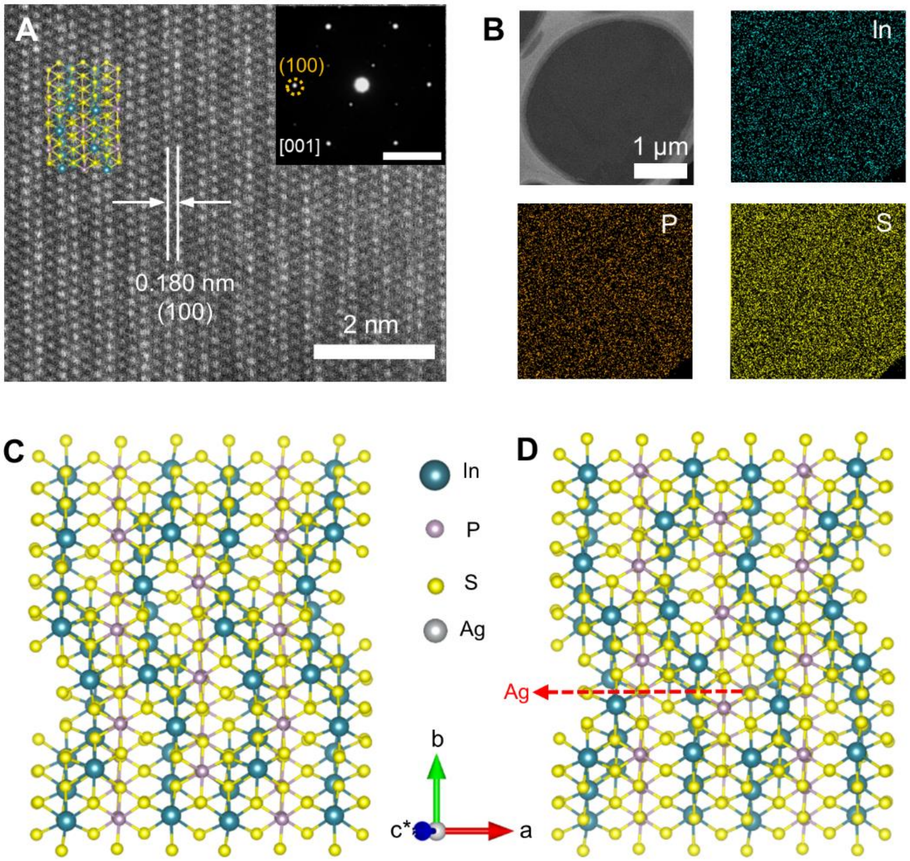

Fig. S10. Structure characterization of IPS. (**A**) Top-view scanning transmission electron microscopy-annular dark field (STEM-ADF) image of the IPS and the corresponding selected area electron diffraction (SAED) image (the inset). The scale bar in the SAED image is 5 1/nm. (**B**) Energy-dispersive X-ray spectroscopy (EDS) mapping of the IPS. The ratio of In:P:S is 2:2.6:7.6, indicating some P and S vacancies. (**C**) The atomic structure of IPS corresponds to the STEM image of Fig. S10A. (**D)** The atomic structure of Ag-IPS corresponds to the STEM image of Fig. 3A. It is noted that the atomic structure model in Figs. S10C and D are viewed from the crystallographic c* axis, which has a deflection angle relative to the normal direction of the (001) plane or ab plane (the atomic model in Fig. 1A is viewed from the normal direction of the ab plane). The IPS belongs to the monoclinic structure (cell parameters: a × b × c < α × β × γ> = 6.842 × 10.528 × 18.266 <90.0 × 107.67 × 90.0>), in which the crystallographic c* axis is not perpendicular to the plane (001). The IPS and Ag-IPS show similar lattice fingers and SAED spots, indicating that the doping hasn't changed the crystal structure. Due to the low doping concentration and the blocking of other atoms, the Ag atoms are difficult to observe in the STEM images. However, the Ag element is verified by the EDS mapping and XPS spectra. As the doping concentration is low, it speculates that the position of Ag is random. Because of the low concentration of Ag, there are limited available ion migration paths, which benefit the confinement of the filament formation.

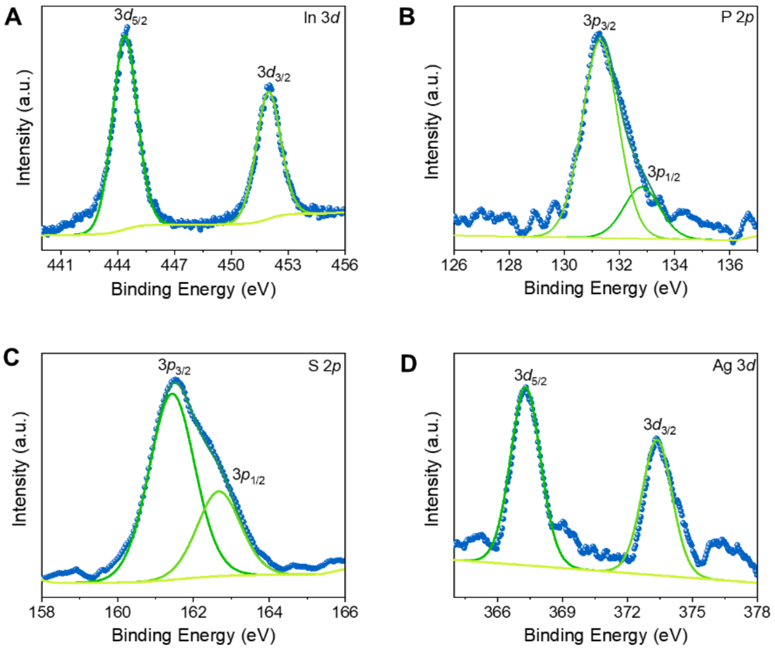

Fig. S11. X-ray photoelectron spectroscopy (XPS) of Ag-IPS. (**A**) In 3*d*, (**B**) P 2*p*, (**C**) S 2*p*, and (**D**) Ag 3*d*. The XPS results indicate the presence of Ag, In, P, and S elements. The peaks at 444.38 and 451.96 eV belong to In $3d_{5/2}$ and In $3d_{3/2}$, respectively (Fig. S11A) (*68*). The peaks at 131.30 (P $2p_{3/2}$) and 132.81 eV (P $2p_{1/2}$) correspond to P^{4+}(Fig. S11B), while the two peaks at 161.43 eV (S $2p_{3/2}$) and 162.67 (S $2p_{1/2}$) are related to S^{2-} in the group of $[P_2S_6]^{4-}$ (Fig. S11C). The obvious Ag element is detected, where the peaks located at 367.30 and 373.37 eV are attributed to Ag $3d_{5/2}$ and Ag $3d_{3/2}$, respectively (Fig. S11D). The atomic ratio of Ag:In:P:S calculated from XPS data is 0.1:2:2.2:4.6, indicating slight doping of Ag.

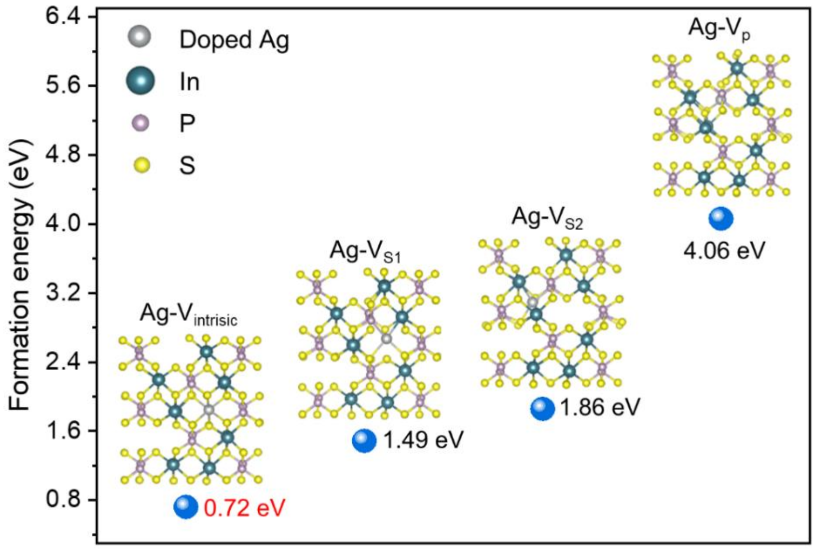

Fig. S12. The formation energy of Ag occupies the different vacancies in IPS. The lowest formation energy is found when Ag occupies the intrinsic vacancy, i.e. Ag-$V_{intrinsic}$.

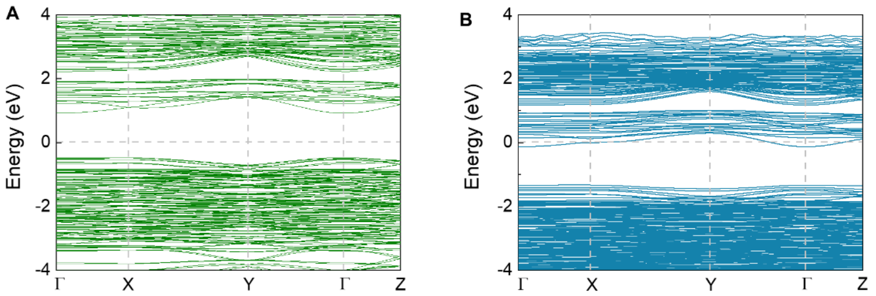

Fig. S13. Energy band structure calculation. (**A**) Pristine IPS, (**B**) Ag-doped IPS. The Fermi level is denoted by a grey dashed line at 0 eV. After Ag doping, the Fermi level shifts to the edge of the conduction band, and the bandgap is narrowed.

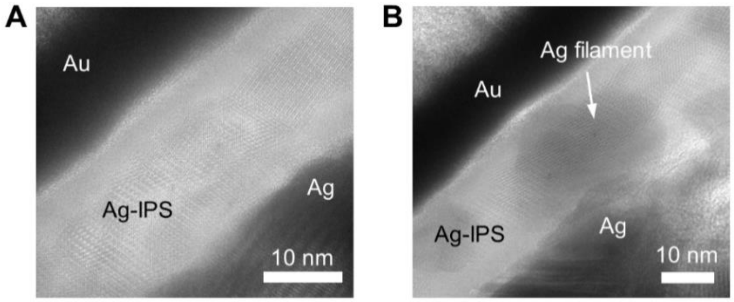

Fig. S14. Resistive mechanism explored by cross-sectional HR-TEM image. (**A**) Un-operated device, (**B**) operated device with Ag filaments.

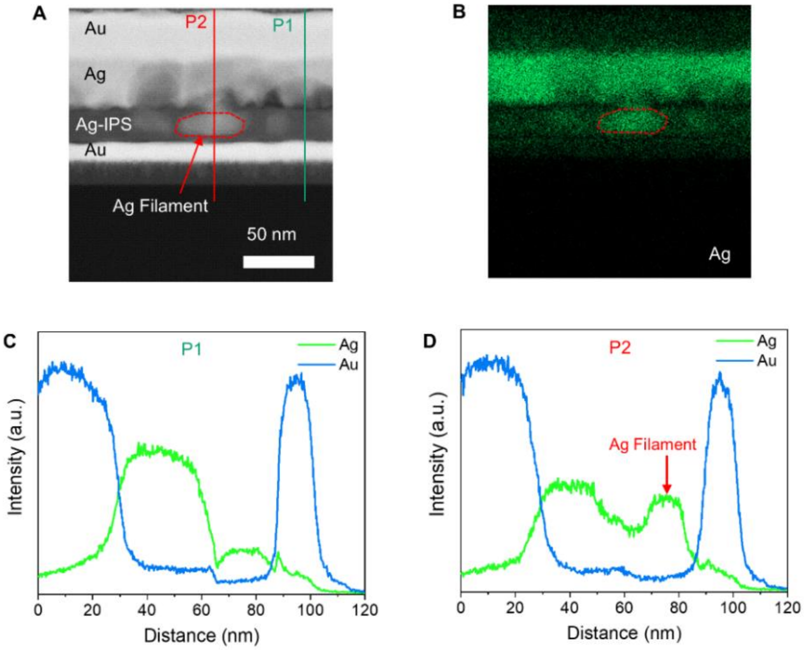

Fig. S15. Cross-sectional STEM and EDS elemental mapping images showing the filament formation. (**A**) Cross-sectional STEM image of the switching-on Ag-IPS memristor and (**B**) the corresponding cross-sectional EDS mapping, as well as the line scan analysis in (**C**) non-conductive filament (CF) position (P1) and (**D**) CF position (P2). These all confirm the formation of Ag filament, verifying the electrochemical metallization mechanism in our device.

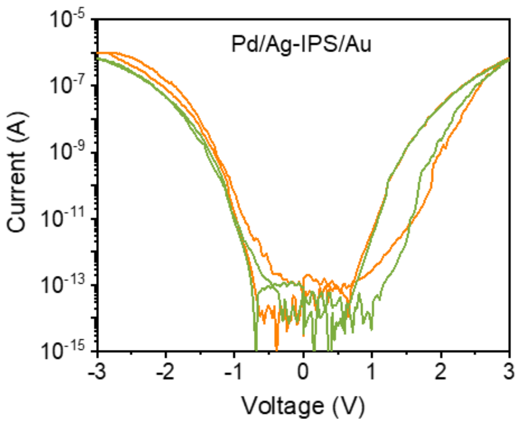

Fig. S16. Resistive switching mechanism explored using inert electrode. *I*-*V* curves of Ag-IPS memristor using Pd as the top inert electrode. When Ag is replaced with Pd as the top electrode (*i.e.*, Pd/Ag-IPS/Au), no RS behavior was observed, indicating that the pre-doped Ag in the Ag-IPS is unable to form the filaments.

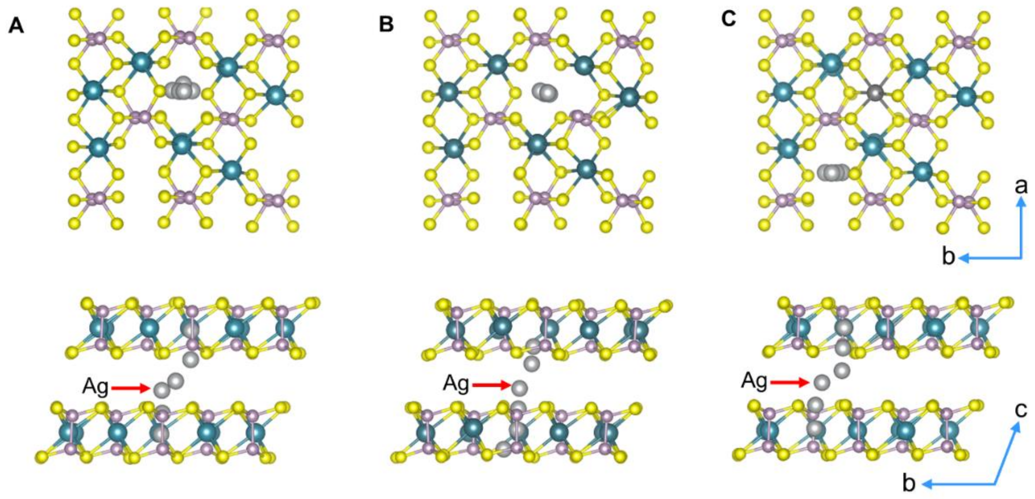

Fig. S17. Schematic showing the Ag ions diffusion pathway. Ag ions diffuse through the adjacent layers via (**A**) the pristine structural vacancy, (**B**) the S vacancy beside the structural vacancy, and (**C**) the structural vacancy near the doped Ag.

Supplementary Note 1: discussion of the pre-doped Ag and post-formed Ag

There are two types of Ag in our memristors: pre-doped Ag formed during the chemical-vapor-transport (CVT) growth (marked as pre-doped Ag) and post-formed Ag from the Ag electrode oxidation under an electric field (marked as post-formed Ag). For the IPS memristor, the post-formed Ag is the only source of Ag. The Ag-IPS memristor contains these two types. The post-formed Ag from the Ag electrode should be unlike the pre-doped Ag. If the post-formed Ag can form a similar doping structure after the memristive operations, uniform RS would also appear in the IPS memristor. We compare the RS curves of the first 100 cycles and the last 100 cycles of the IPS memristor. In Fig. S18, the IPS memristor displays a similar distribution of *I*-*V* curves between the first 100 cycles and the last 100 cycles. Moreover, the last 100 cycles have larger variations of the operation voltages than the first 100 cycles. It indicates that there is no Ag doping formation during the operation, or the doping/occupation formed by the post-formed Ag from the Ag

electrode is different from the pre-doped Ag, which cannot introduce uniform RS behavior. From the forming curves in Fig. S2, the HRS resistance is similar to its initial resistance in both IPS and Ag-IPS memristors, which verifies that there are no or very few residual Ag atoms in HRS. In other words, the post-formed Ag atoms are difficult to maintain in the switching medium after the RESET operation and unable to form an effective doping structure. This is attributed to the high activity of Ag and ultralow diffusion barrier, allowing the filament to be totally dissolved, and the Ag ions can then quickly diffuse back to the electrode.

The results of the DFT calculation show that Ag prefers to occupy the intrinsic structural vacancy; however, whether a stable occupation/doping structure can be formed depends on the actual energy conditions. Moreover, it is noted that the occupation energy is positive. In other words, enough energy should be provided to form a stable doping structure. The electric field may not be able to offer enough energy to form the stable doping structure with the post-formed Ag. Hence, the post-formed Ag is more likely to form weak connections with the surrounding atoms in the switching medium and can leave the occupying position to migrate toward the counter electrode under the electric field. For CVT growth, the high temperature and long growth time provide enough energy, which leads to stable Ag doping with stronger connections with the surrounding atoms (Ag-IPS). Thus, once the stable Ag doping has been formed, considerable energy is also required to make the doping Ag leave the position. Thus, it speculates that the pre-doped Ag is unlikely to move and be substituted by the post-formed Ag. The position of the pre-doped Ag is certain after synthesis. Such pre-doped Ag cannot give rise to RS, as shown in Fig. S16. However, stable Ag pre-doping with specific positions can pre-introduce low-power migrating paths for post-formed Ag. Moreover, due to the low Ag doping, the number of migration paths is limited. As a result, filament formation is confined, leading to a uniform RS behavior. Therefore, based on experimental results, it speculates that the pre-doped Ag is unlike the post-formed Ag.

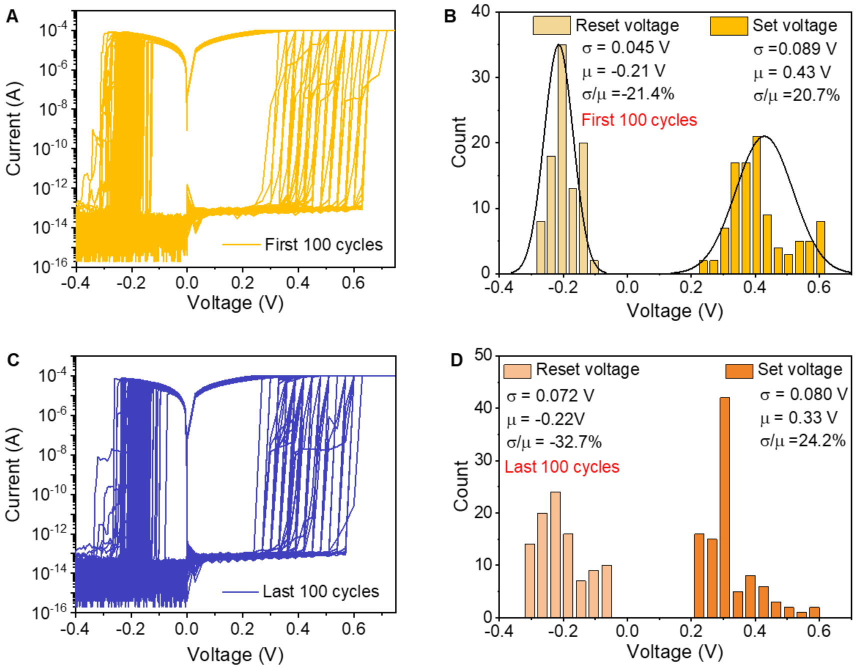

Fig. S18. Comparison of the *I-V* curves and corresponding operation voltage distributions of the first 100 cycles and the last 100 cycles in the IPS memristor. (**A, B**) the first 100 cycles, (**C, D**) the last 100 cycles.

Supplementary Note 2: discussion of the Ag doping enhanced nonvolatile RS

In our ECM memristor, the active metal Ag is used as the top electrode (TE) and source of migration ions, whereas the inert metal Au serves as the bottom electrode (BE). The creation of the diffusion channel (or the conduction channel) is related to the electrochemical and diffusive dynamics of the metal ions. Typically, when a positive bias is applied to the active Ag electrode, the metal Ag is oxidized into Ag ions near the active TE. Subsequently, the Ag ions drift to the inert BE driven by the electric field, where conductive channels that result from the nucleation and growth of Ag clusters/filaments are developed between the electrodes through the redox reaction. The creation of the conductive channel relates to the compliance current (I_{CC}). Compared with the thin IPS memristor, the thick IPS memristor has a longer migration distance making more Ag ions contribute to the formation of conductive filament (CF), thus a more stable filament. Therefore, the thick IPS can show a lower non-volatile programming current. However, in the thin IPS memristor with the short migration path, the Ag ions would quickly move to the bottom electrode and then are reduced there forming the CF. Due to the fast speed, a limited number of Ag ions participate in the formation of CFs, thus a thin and less stable CF. Therefore, the compliance current has a more obvious influence on the thin IPS memristor.

Specifically, with a low I_{CC}, small metal atom clusters or thin metal filaments will form in the thin IPS memristor. Due to the small size and low activation energy, the Ag would be reduced and diffuse back spontaneously when the electric field decreases or stops, which leads to the volatile RS behavior with diffusive dynamic behavior, as shown in Fig. S19A. This type of memristor is also called diffusive memristor or threshold memristor, which is characterized by an unstable metallic conductive path with spontaneous rupture (*69-71*). With a relatively high I_{CC}, stable thick metal filaments likely appear, which results in non-volatile RS with the switching dynamics dominated by the drift kinetics, as shown in Fig. S19B. This type of memristor portraying non-volatile memory characteristics is also called a drift memristor. Furthermore, in the IPS memristor, metal cations from the Ag electrode can migrate through various alternative pathways, limiting the number of Ag ions available to form the final conductive filament, as schematically illustrated by the yellow arrows in Figs. S19A and B. This further enhances the difficulty of forming stable filaments under a low I_{CC} in the thin IPS memristor. For the Ag-IPS device, the Ag doping creates a low-diffusion-barrier and concentrated formation path; thus, more Ag ions from the electrode will choose to migrate around the doping path, as shown in Supplementary Fig. 19C. Thus, stable and thick metal filaments likely appear, which results in a non-volatile RS at a low I_{CC}.

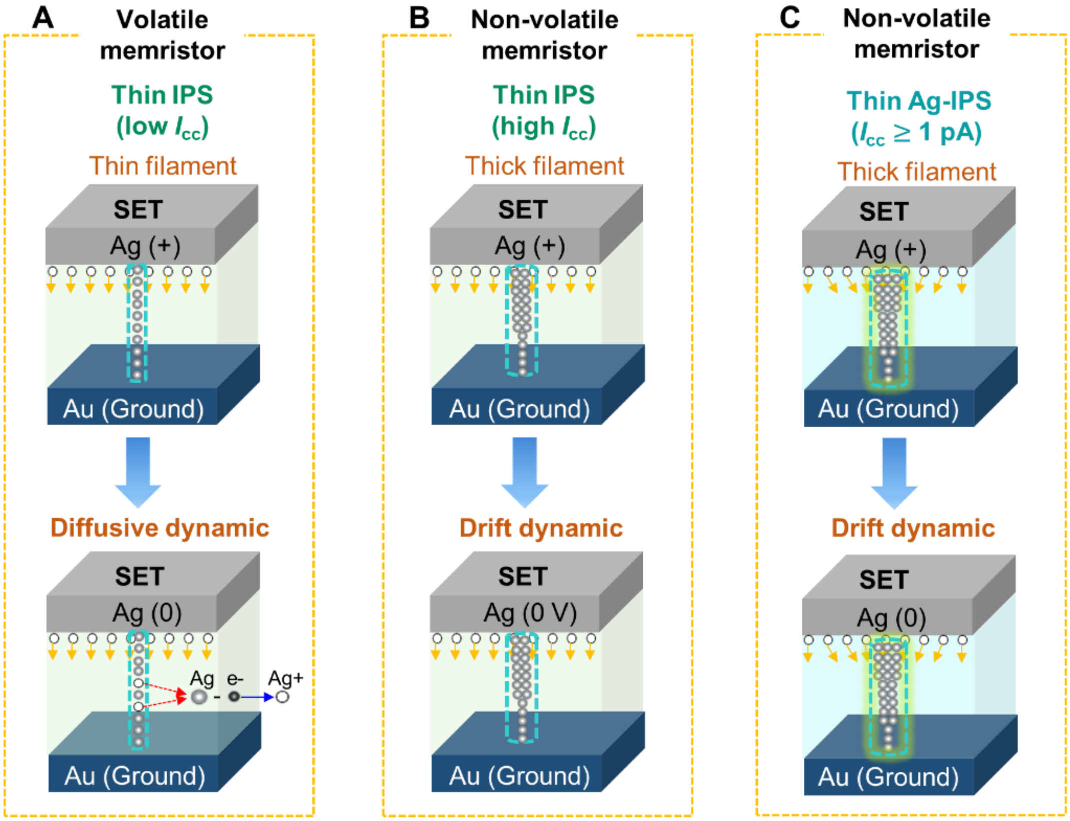

Fig. S19. Schematically showing the filament formation in the IPS and Ag-IPS memristors. (**A**) Volatile diffusive switching at low I_{cc}, (b) non-volatile drift switching at high I_{cc} in the IPS memristor, (**C**) non-volatile drift switching in the Ag-IPS memristor. The yellow arrows indicate the migrating direction of Ag ions. In the IPS memristor, there are many similar diffusion pathways around the Ag ions, thus the Ag ions can migrate from one at random. While in the Ag-IPS memristor, the Ag doping provides the preferable pathway that the more Ag ions would migrate around the doping path for a stable filament.

In addition, because of the low Ag doping, the HRS resistance of Ag-IPS memristor only shows a limited decrease compared to its intrinsic sample. As shown in Fig. S2, the $R_{initial}$ values of IPS and Ag-IPS memristors (~ 40 nm) are 7.5 × 10^{11} ohm and 4.3 × 10^{11} ohm, respectively. The doped sample exhibits a lower resistance than the un-doped device due to the bandgap narrowing induced by doping. However, because the doping concentration is very low, the resistance change is not very obvious. Both IPS and Ag-IPS memristors show similar values of HRS resistance with each initial resistance. Similar to the variation in the initial resistance, the Ag-IPS memristor has only slightly lower HRS resistance than the IPS device, as displayed in Supplementary Fig. 5. The high value of HRS resistance enables the Ag-IPS memristors to have large on/off ratios.

The lower HRS resistance in the Ag-IPS memristor is not always observed, especially in the thin samples. The most important reason is that the light doping with a low Ag content leads to limited resistance change between IPS and Ag-IPS samples. Second, the HRS resistance may be influenced by the forming process (which may introduce more defects), contact between the switching medium and the electrode, residual Ag filament/atoms during operation, etc. These factors further weaken the influence of doping on the HRS resistance. Third, both HRS resistances of IPS and Ag-IPS samples are very large, which is approximately the measurement limit of the

equipment, so they introduce some deviations. In particular, the factors of the forming process and interface contact may have a more obvious influence on the HRS resistance of the thin device, which leads to higher resistance. The increased HRS resistance, which is closer to the measurement limit compared to the thick sample, causes more test noise, as shown in Fig. 2. Therefore, the resistance changes between IPS and Ag-IPS memristors are not very obvious, especially in the thin samples. Only in the doped thick samples, which have HRS current higher than the measure limit, the reduced HRS resistance can be observed.

Supplementary Note 3: logic-in-memory computing

The memory wall problem in the von Neumann architecture will become increasingly serious in the era of data-centric computing. Logic-in-memory (LIM) computing, which simultaneously performs the memory and logic operations arises as an attractive computing paradigm for in-memory computing and will overcome some issues concerning the von Neumann bottleneck. Memristors have been considered the most suitable candidate for logic-in-memory computing. Our memristor with an ultrahigh on-off ratio and good retention is extremely promising for LIM. Here, 14 logic functions were implemented using two strategies. The top Ag electrode and bottom Au electrode were assigned to *In*1 and *In*2, respectively. For the first strategy, both top Ag and bottom Au electrodes were used to apply the voltage bias (the two-terminal strategy), as shown in Fig. S20A. When operating the logic operations of *In*1*In*2, e.g., 01, the low potential pulse of 0 V that corresponds to '0' is applied to the top electrode (TE), and the high potential pulse of 2 V corresponding to '1' is simultaneously applied to the bottom electrode. Fig. S21 shows the corresponding results. The second strategy is a simplified method, where the TE is employed to apply the voltage bias, and the BE is grounded (the one-terminal strategy), as shown in Fig. S20B. In other words, when operating the logic functions *In*1*In*2 of 00, 01, 10, and 11, the voltage bias of 0 V, −2 V, 2 V, and 0 V are applied to the top electrode, respectively. Fig. S23 shows the operation results.

The experiments with the same number are identical; for example, Experiment IV in Fig. S21 for logic functions of *p* IMP *q*, *p* RIMP *q*, *p*, and *q* are the same. The initialization operation with the same voltage waveform is conducted before every logical operation sequence. The initialization operation serves the following purposes:

(1) To initialize the device to HRS. Thus, all logic operations have identical initial states.
(2) To check whether the device can be correctly SET and RESET by the pulse voltages.
(3) To define the states '0' and '1'. The initial SET and RESET operations can visually show the LRS and HRS states or '0' and '1'. Then, we can quickly determine the output logic value.
(4) To obtain the current of '0' or '1' and plot the histogram of the current distribution. Take the logic experiment III in Fig. S21 as an example, the current of state '1' is achieved from the output current, while the current of state '0' is obtained from the initialization step in experiment III. From the histogram, we can further confirm the well-distinguished output logic '0', which indicates the reliable logic operation.

The initialization step is not necessary. The currents between HRS and LRS in our device show a big gap, based on which the output logic value can be determined from the current value, as shown in Fig. S22. However, the initialization step is beneficial for quickly checking the output logic value and confirming the logic reliability by comparing the output logic current with its initial opposite-logic current.

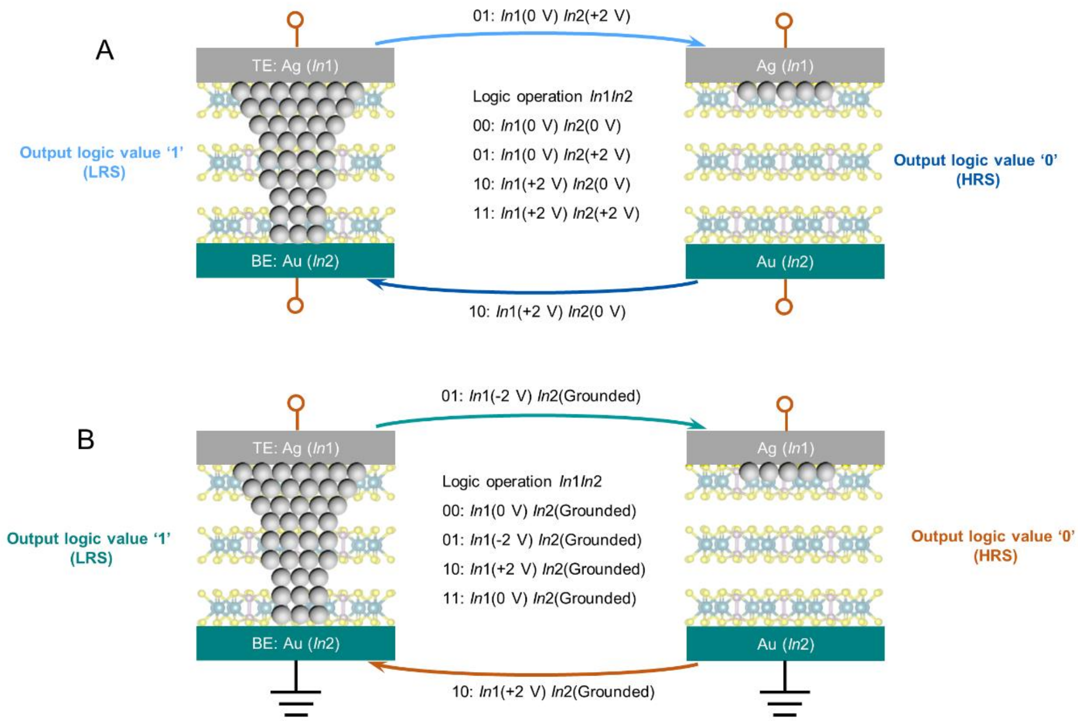

Fig. S20. Operating the logic functions by a single device using two strategies. (**A**) The two-terminal strategy. Both the top electrode (TE, *i.e.*, terminal *In*1) and bottom electrode (BE, *i.e.*, terminal *In*2) as input terminals for applying voltage bias, where '0' represents applying a low potential (0 V) and '1' means applying a high potential (2 V). For example, when operating logic (10), the high potential of 2 V will be applied to the terminal *In*1 and the low potential of 0 V will be applied to the terminal *In*2 at the same time. (**B**) The one-terminal strategy. Only the TE is used as the input terminal for applying voltage bias, while the BE is always grounded. When operating logic functions of 00, 01, 10, and 11, the voltage bias of 0 V, −2 V, 2 V, and 0 V are applied to the TE, respectively.

Logic function	Operation1		Operation1			
	In1	In2	*p*	*q*	Read	Exp.
1.True (1)	1	0	0	0	1	I
	1	0	1	0	1	I
	1	0	0	1	1	I
	1	0	1	1	1	I

Logic function	Operation1		Operation1			
	In1	In2	*p*	*q*	Read	Exp.
2. False (0)	0	1	0	0	0	II
	0	1	1	0	0	II
	0	1	0	1	0	II
	0	1	1	1	0	II

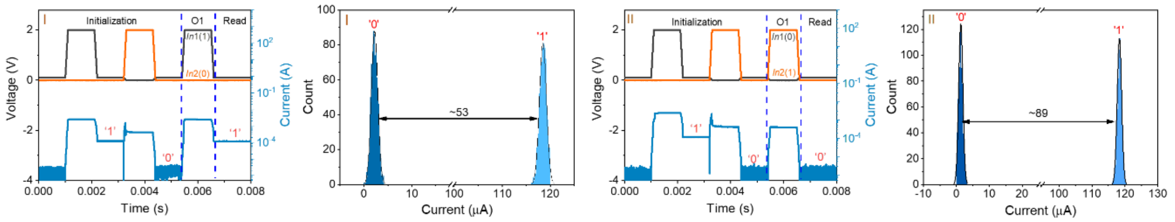

Logic function	Operation1		Operation2		Operation 1,2			
	In1	In2	In1 = *q*	In2 = *p*	*p*	*q*	Read	Exp.
3. *p* IMP *q* ($\overline{p}$ + *q*)	1	0	0	0	0	0	1	III
	1	0	0	1	1	0	0	IV
	1	0	1	0	0	1	1	V
	1	0	1	1	1	1	1	III-2

Logic function	Operation1		Operation2		Operation 1,2			
	In1	In2	In1 = *p*	In2 = *q*	*p*	*q*	Read	Exp.
4. *p* RIMP *q* (*p*+ $\overline{q}$)	1	0	0	0	0	0	1	III
	1	0	1	0	1	0	1	V
	1	0	0	1	0	1	0	IV
	1	0	1	1	1	1	1	III-2

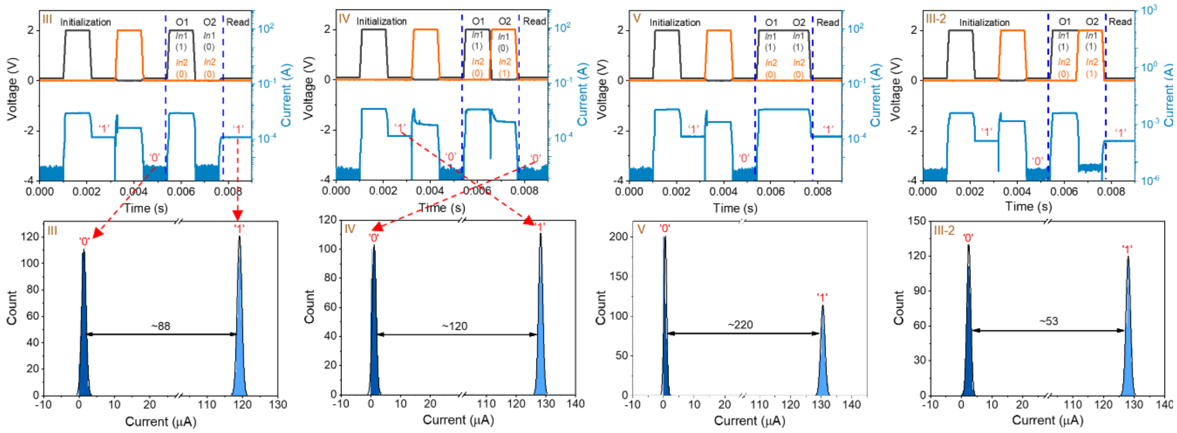

Logic function	Operation1		Operation2		Operation 1,2			
	In1	In2	In1 = *p*	In2 = *q*	*p*	*q*	Read	Exp.
5. *p* NIMP *q* (*p* $\overline{q}$)	0	1	0	0	0	0	0	VI
	0	1	1	0	1	0	1	VII
	0	1	0	1	0	1	0	VIII
	0	1	1	1	1	1	0	VI-2

Logic function	Operation1		Operation2		Operation 1,2			
	In1	In2	In1 = *q*	In2 = *p*	*p*	*q*	Read	Exp.
6. *p* RNIMP *q* ($\overline{p}$ *q*)	0	1	0	0	0	0	0	VI
	0	1	0	1	1	0	0	VIII
	0	1	1	0	0	1	1	VII
	0	1	1	1	1	1	0	VI-2

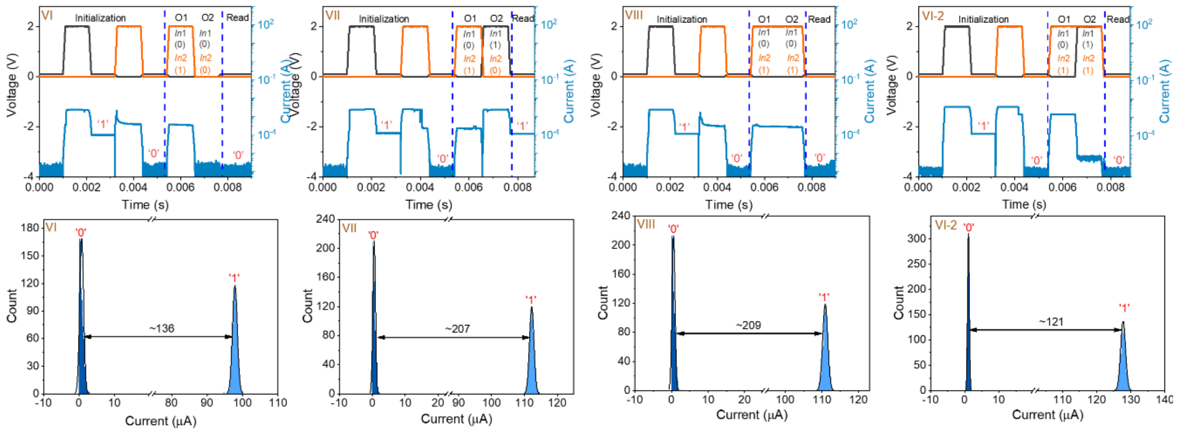

Logic function	Operation1		Operation2		Operation 1,2			
	In1	In2	In1 = *p*	In2 = *1*	*p*	*q*	Read	Exp.
**7.** $p\,(p)$	1	0	0	1	0	0	0	IV
	1	0	1	1	1	0	1	III-2
	1	0	0	1	0	1	0	IV
	1	0	1	1	1	1	1	III-2

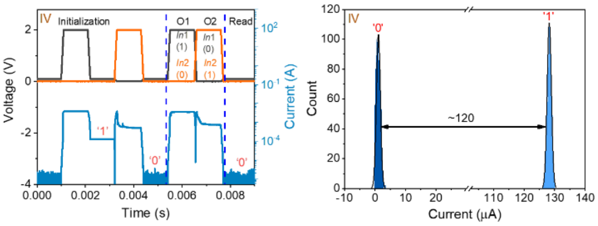

Logic function	Operation1		Operation2		Operation 1,2			
	In1	In2	In1 = *q*	In2 = *1*	*p*	*q*	Read	Exp.
**8.** $q\,(q)$	1	0	0	1	0	0	0	IV
	1	0	0	1	1	0	0	IV
	1	0	1	1	0	1	1	III-2
	1	0	1	1	1	1	1	III-2

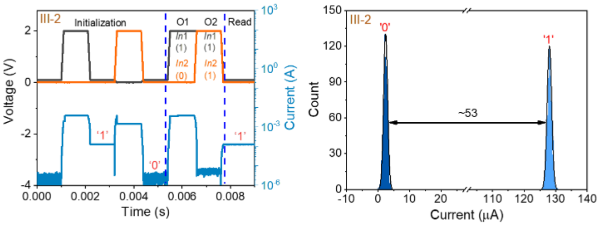

Logic function	Operation1		Operation2		Operation 1,2			
	In1	In2	In1 = 1	In2 = *q*	*p*	*q*	Read	Exp.
**9. NOT *q*** $(\bar{q})$	0	1	1	0	0	0	1	VII
	0	1	1	0	1	0	1	VII
	0	1	1	1	0	1	0	VI-2
	0	1	1	1	1	1	0	VI-2

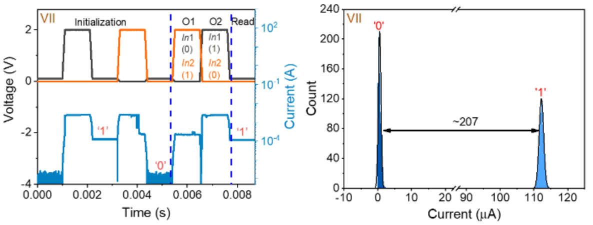

Logic function	Operation1		Operation2		Operation 1,2			
	In1	In2	In1 = 1	In2 = *p*	*p*	*q*	Read	Exp.
**10. NOT *p*** $(\bar{p})$	0	1	1	0	0	0	1	VII
	0	1	1	1	1	0	0	VI-2
	0	1	1	0	0	1	1	VII
	0	1	1	1	1	1	0	VI-2

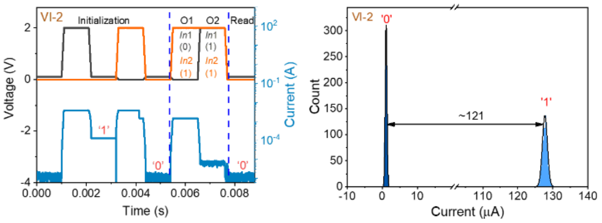

Logic function	Operation1		Operation2		Operation3		Operation 1,2,3			
	In1	In2	In1	In2	In1 = *q*	In2 = 0	*p*	*q*	Read	Exp.
**11.** p **OR** q $(p+q)$	1	0	0	1	0	0	0	0	0	IX
	1	0	1	1	0	0	1	0	1	X-2
	1	0	0	1	1	0	0	1	1	XI
	1	0	1	1	1	0	1	1	1	XII-2

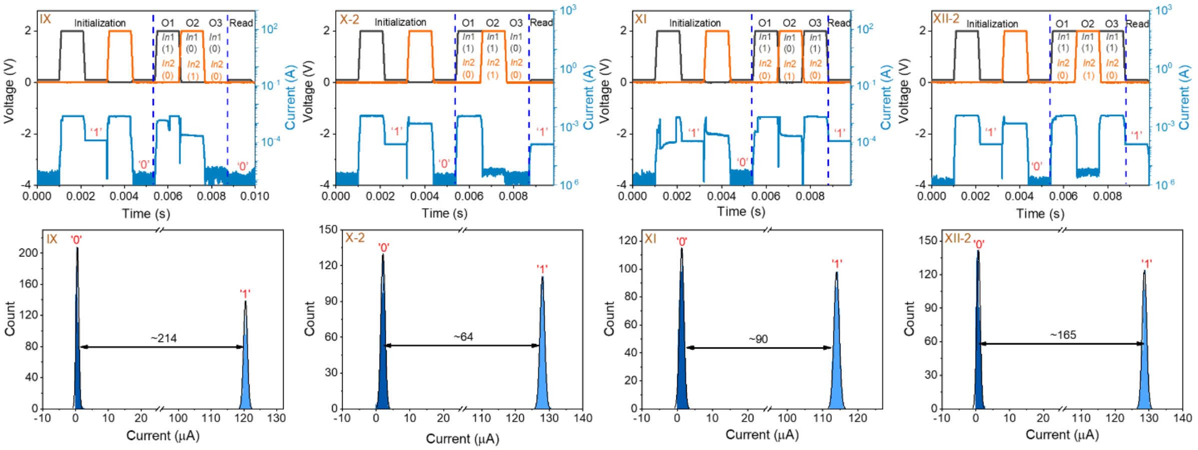

Logic function	Operation1		Operation2		Operation3		Operation 1,2,3			
	In1	In2	In1=0	In2= q	In1 = 0	In2 = p	p	q	Read	Exp.
12. p NORq ($\overline{pq}$)	1	0	0	0	0	0	0	0	1	X
	1	0	0	0	0	1	1	0	0	XIII
	1	0	0	1	0	0	0	1	0	IX
	1	0	0	1	0	1	1	1	0	XIV

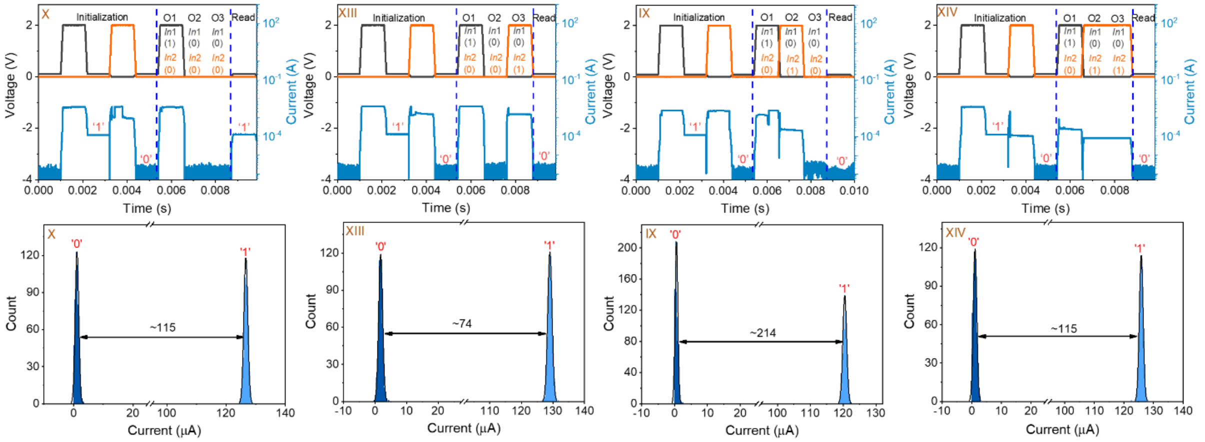

Logic function	Operation1		Operation2		Operation3		Operation 1,2,3			
	In1	In2	In1= p	In2= 1	In1 = q	In2 = 1	p	q	Read	Exp.
13. p ANDq (pq)	1	0	0	1	0	1	0	0	0	XIV
	1	0	1	1	0	1	1	0	0	XIII-2
	1	0	0	1	1	1	0	1	0	IX-2
	1	0	1	1	1	1	1	1	1	X-3

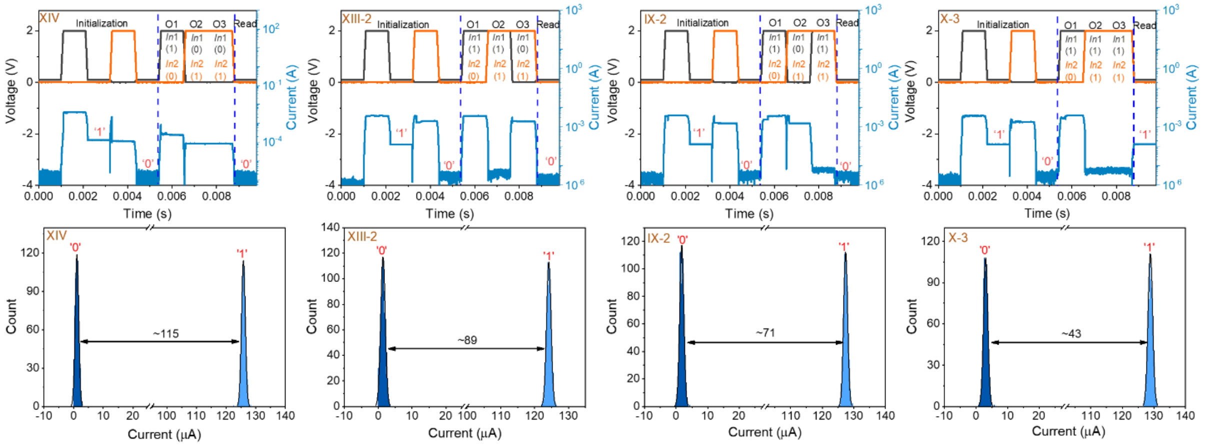

Logic function	Operation1		Operation2		Operation3		Operation 1,2,3			
	In1	In2	In1	In2= q	In1 = 1	In2 = p	p	q	Read	Exp.
14. p NANDq ($\bar{p}+\bar{q}$)	1	0	0	0	1	0	0	0	1	XII
	1	0	0	0	1	1	1	0	1	X-4
	1	0	0	1	1	0	0	1	1	XI
	1	0	0	1	1	1	1	1	0	IX-2

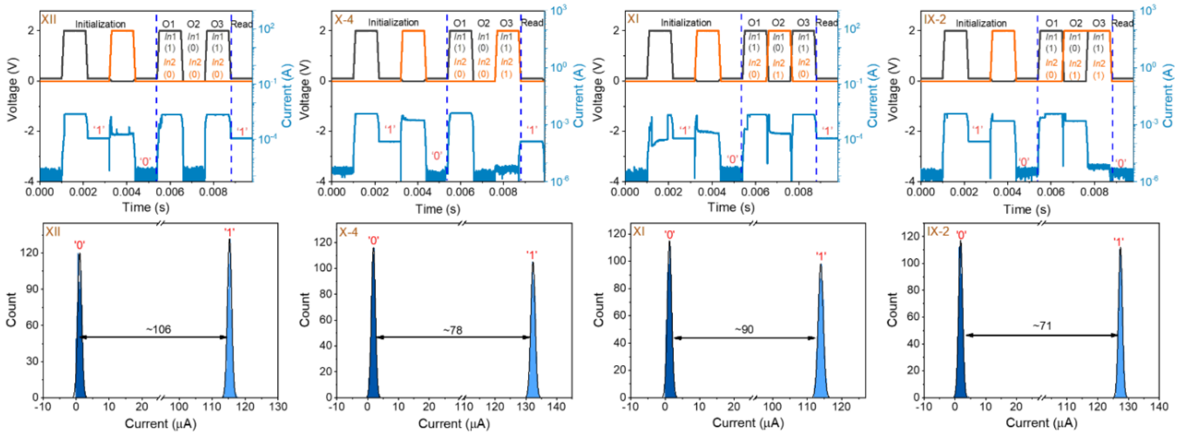

Fig. S21. Experimental demonstration of the fourteen basic Boolean logic operations using two input terminals (the two-terminal strategy). In each logic function, the above panel is the truth table showing the sequence of operations and the below panel is the corresponding experimental demonstration of logic operation. An initial operation is performed prior to the logic operations to initialize the device to HRS as well as define the states ‘0’ and ‘1’, as displayed in the ‘initialization’ regime. Then, the pulse operation(s) corresponding to the truth table is followed to execute the logic operation. Finally, a readout pulse (0.1 V) is followed to read out the current, i.e., the logic value. Here, the HRS refers to state ‘0’ and the LRS represents state ‘1’. The state ‘1’ and state ‘0’ can be clearly distinguished for all operations, indicating reliable logic computations. Experiments III and IV in logic function p IMP q illustrate the data source of the histogram of the current distribution. The experiment results of all 14 logic functions are consistent with the truth table of Boolean functions. The realization of logic operations in our nonvolatile memristor presents a promising candidate for developing in-memory computing architectures.

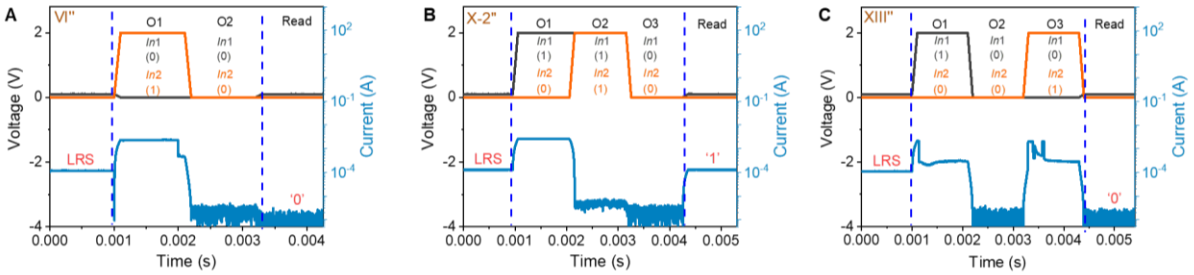

Fig. S22. Experimental demonstration of logic operation without initialization step. The device is in HRS before the logic operation in Fig. S21. Fig. S22 shows that the logic operation also can be performed when the initial state of the device is LRS. That is to say, whatever the initial state of the device is, the logic operation can be conducted successfully. The initialization step is not the necessary step.

Logic function	Operation1		Operation1			
	In1	In2	*p*	*q*	Read	Exp.
	1	0	0	0	1	I
1.True (1)	1	0	1	0	1	I
	1	0	0	1	1	I
	1	0	1	1	1	I

Logic function	Operation1		Operation1			
	In1	In2	*p*	*q*	Read	Exp.
	0	1	0	0	0	II
2. False (0)	0	1	1	0	0	II
	0	1	0	1	0	II
	0	1	1	1	0	II

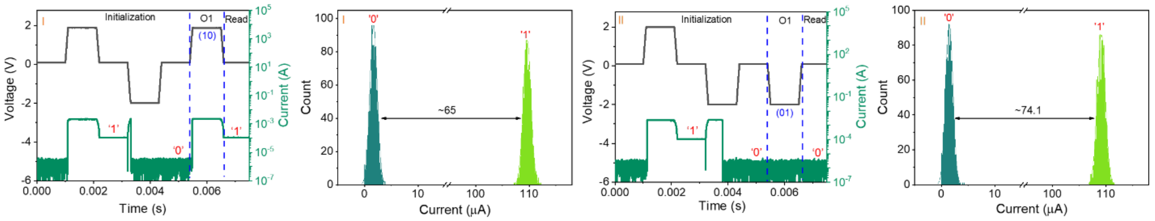

Logic function	Operation1		Operation2		Operation 1,2			
	In1	In2	In1 = *q*	In2 = *p*	*p*	*q*	Read	Exp.
3. *p* IMP*q* ($\overline{p}$ + *q*)	1	0	0	0	0	0	1	III
	1	0	0	1	1	0	0	IV
	1	0	1	0	0	1	1	V
	1	0	1	1	1	1	1	III

Logic function	Operation1		Operation2		Operation 1,2			
	In1	In2	In1 = *p*	In2 = *q*	*p*	*q*	Read	Exp.
4. *p* RIMP*q* (*p*+ $\overline{q}$)	1	0	0	0	0	0	1	III
	1	0	1	0	1	0	1	V
	1	0	0	1	0	1	0	IV
	1	0	1	1	1	1	1	III

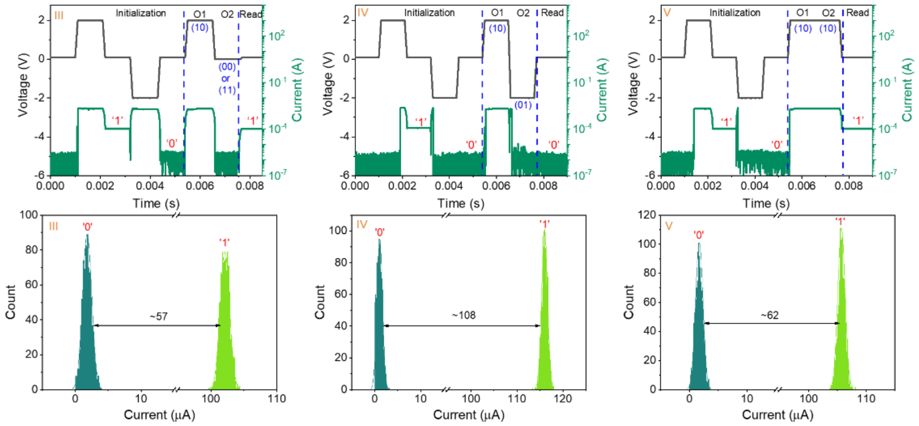

Logic function	Operation1		Operation2		Operation 1,2			
	In1	In2	In1 = *p*	In2 = *q*	*p*	*q*	Read	Exp.
5. *p* NIMP*q* (*p* $\overline{q}$)	0	1	0	0	0	0	0	VI
	0	1	1	0	1	0	1	VII
	0	1	0	1	0	1	0	VIII
	0	1	1	1	1	1	0	VI

Logic function	Operation1		Operation2		Operation 1,2			
	In1	In2	In1 = *q*	In2 = *p*	*p*	*q*	Read	Exp.
6. *p* RNIMP*q* ($\overline{p}$*q*)	0	1	0	0	0	0	0	VI
	0	1	0	1	1	0	0	VIII
	0	1	1	0	0	1	1	VII
	0	1	1	1	1	1	0	VI

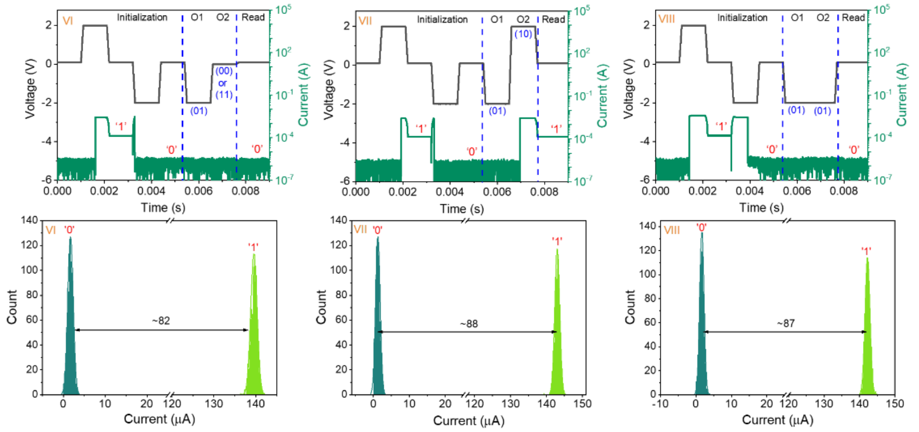

Logic function	Operation1		Operation2		Operation 1,2			
	In1	In2	In1 = *p*	In2 = *1*	*p*	*q*	Read	Exp.
7. p (p)	1	0	0	1	0	0	0	IV
	1	0	1	1	1	0	1	III
	1	0	0	1	0	1	0	IV
	1	0	1	1	1	1	1	III

Logic function	Operation1		Operation2		Operation 1,2			
	In1	In2	In1 = *q*	In2 = *1*	*p*	*q*	Read	Exp.
8. q (q)	1	0	0	1	0	0	0	IV
	1	0	0	1	1	0	0	IV
	1	0	1	1	0	1	1	III
	1	0	1	1	1	1	1	III

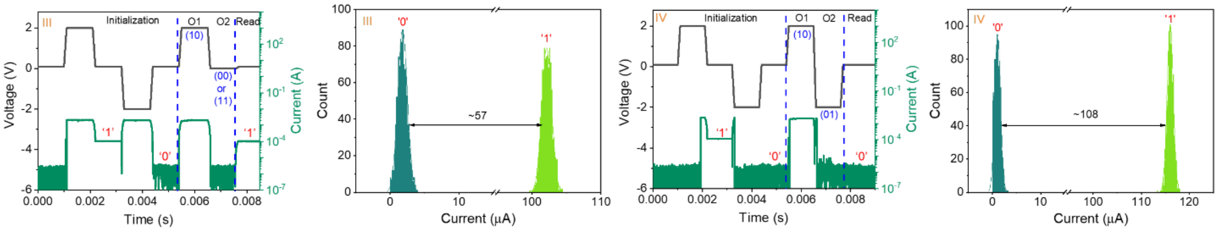

Logic function	Operation1		Operation2		Operation 1,2			
	In1	In2	In1 = 1	In2 = *q*	*p*	*q*	Read	Exp.
9. NOT *q* ($\bar{q}$)	0	1	1	0	0	0	1	VII
	0	1	1	0	1	0	1	VII
	0	1	1	1	0	1	0	VI
	0	1	1	1	1	1	0	VI

Logic function	Operation1		Operation2		Operation 1,2			
	In1	In2	In1 = 1	In2 = *p*	*p*	*q*	Read	Exp.
10. NOT *p* ($\bar{p}$)	0	1	1	0	0	0	1	VII
	0	1	1	1	1	0	0	VI
	0	1	1	0	0	1	1	VII
	0	1	1	1	1	1	0	VI

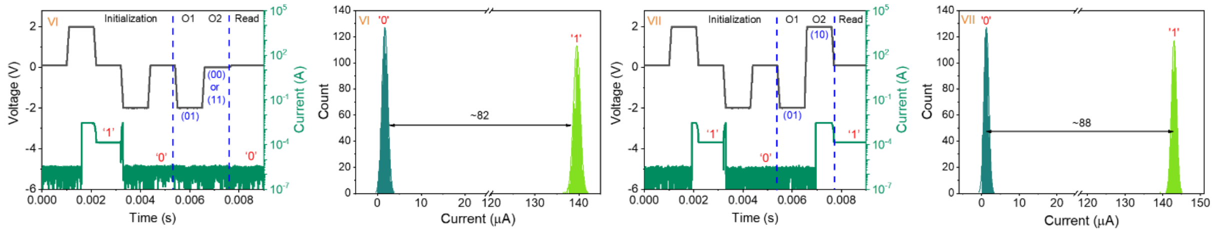

Logic function	Operation1		Operation2		Operation3		Operation 1,2,3			
	In1	In2	In1	In2	In1 = *q*	In2 = 0	*p*	*q*	Read	Exp.
11. p OR q (p+q)	1	0	0	1	0	0	0	0	0	IX
	1	0	1	1	0	0	1	0	1	X
	1	0	0	1	1	0	0	1	1	XI
	1	0	1	1	1	0	1	1	1	XII

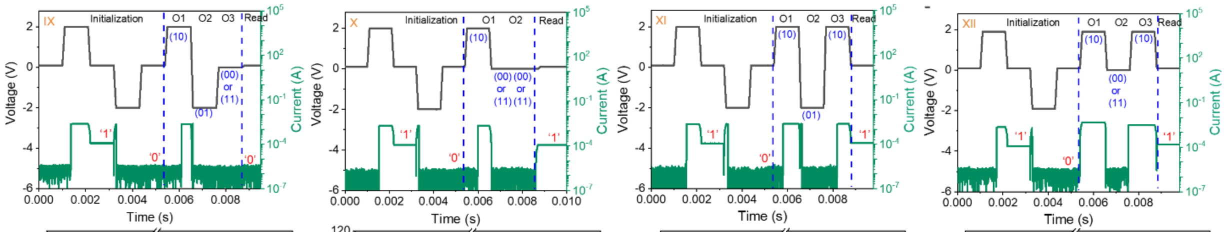

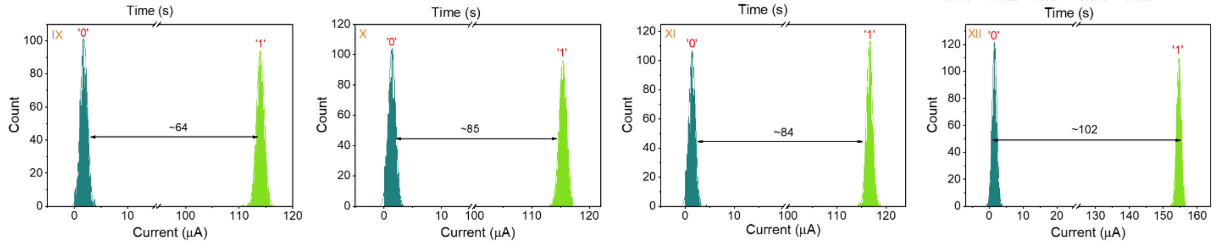

Logic function	Operation1		Operation2		Operation3		Operation 1,2,3			
	In1	In2	In1=0	In2= q	In1 = 0	In2 = p	p	q	Read	Exp.
12. p NORq ($\overline{p}\overline{q}$)	1	0	0	0	0	0	0	0	1	X
	1	0	0	0	0	1	1	0	0	XIII
	1	0	0	1	0	0	0	1	0	IX
	1	0	0	1	0	1	1	1	0	XIV

Logic function	Operation1		Operation2		Operation3		Operation 1,2,3			
	In1	In2	In1= p	In2= 1	In1 = q	In2 = 1	p	q	Read	Exp.
13. p ANDq (pq)	1	0	0	1	0	1	0	0	0	XIV
	1	0	1	1	0	1	1	0	0	XIII
	1	0	0	1	1	1	0	1	0	IX
	1	0	1	1	1	1	1	1	1	X

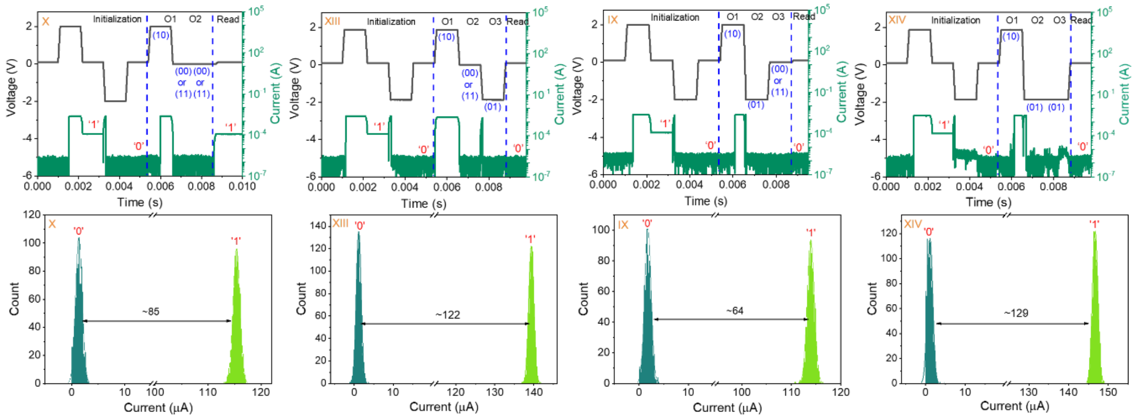

Logic function	Operation1		Operation2		Operation3		Operation 1,2,3			
	In1	In2	In1	In2= q	In1 = 1	In2 = p	p	q	Read	Exp.
14. p NANDq ($\overline{p}+\overline{q}$)	1	0	0	0	1	0	0	0	1	XII
	1	0	0	0	1	1	1	0	1	X
	1	0	0	1	1	0	0	1	1	XI
	1	0	0	1	1	1	1	1	0	IX

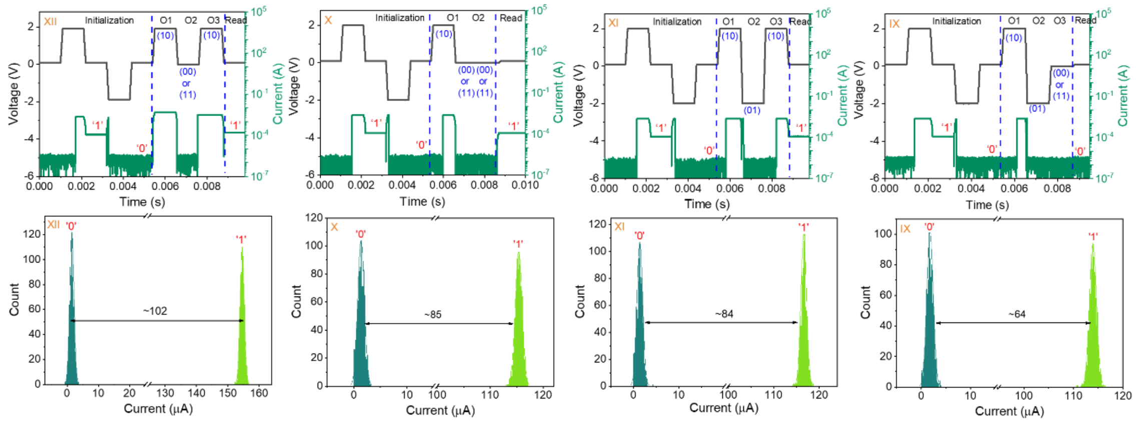

Fig. S23. Experimental demonstration of the fourteen basic Boolean logic operations using the top electrode as input terminal (the one-terminal strategy). The operating scheme is similar to the first strategy, i.e., initialization, operation, and readout, except that the combination approach is employed, in which the voltage bias of 0 V, −2 V, 2 V, and 0 V corresponding to 00, 01, 10, and 11, respectively, are applied to the top electrode. The state ‘1’ and state ‘0’ can be clearly distinguished for all operations and the experiment results of all 14 logic functions are consistent with the truth tables, indicating reliable logic computations.

Convolutional image processing with Ag-IPS memristor crossbar array

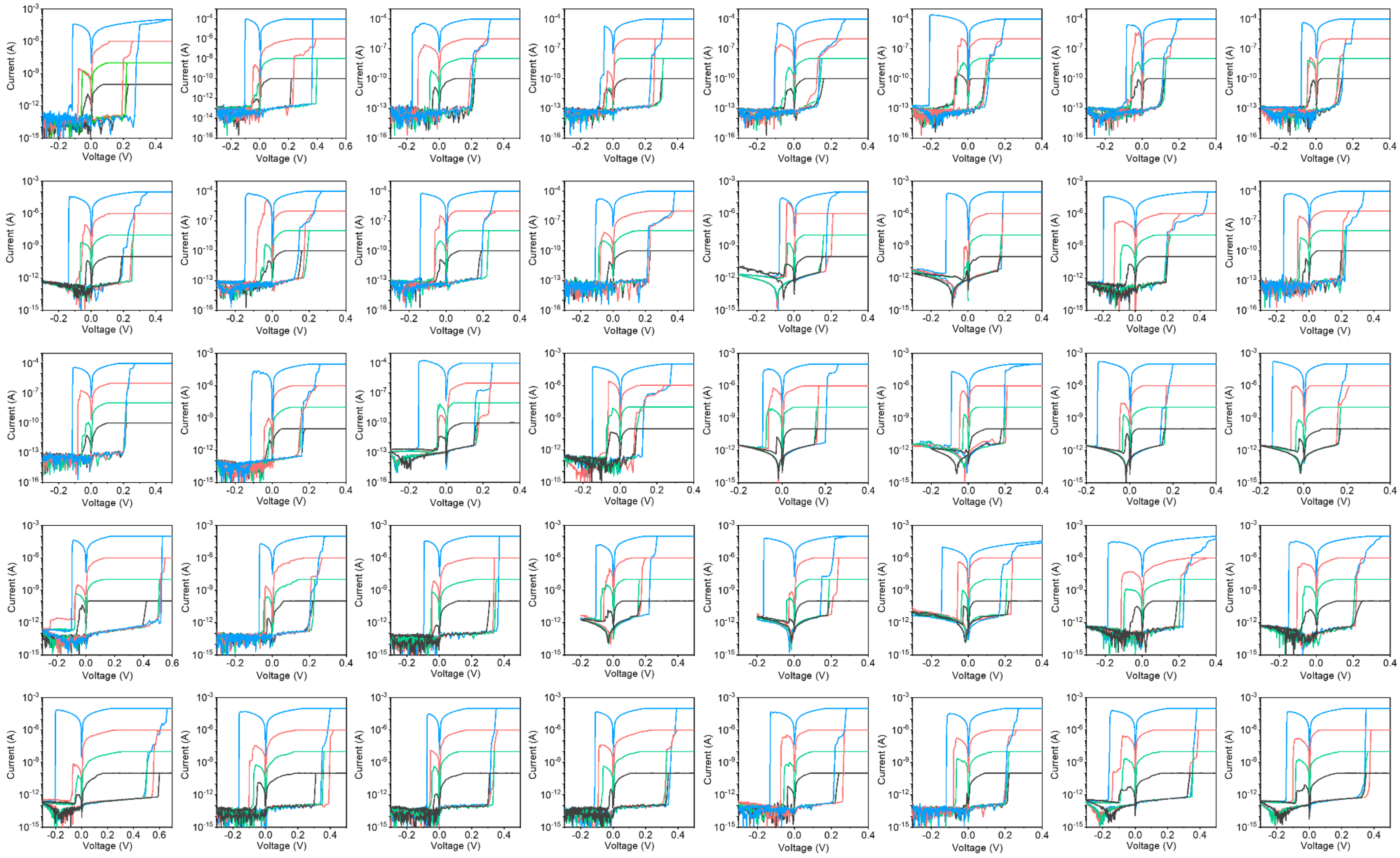

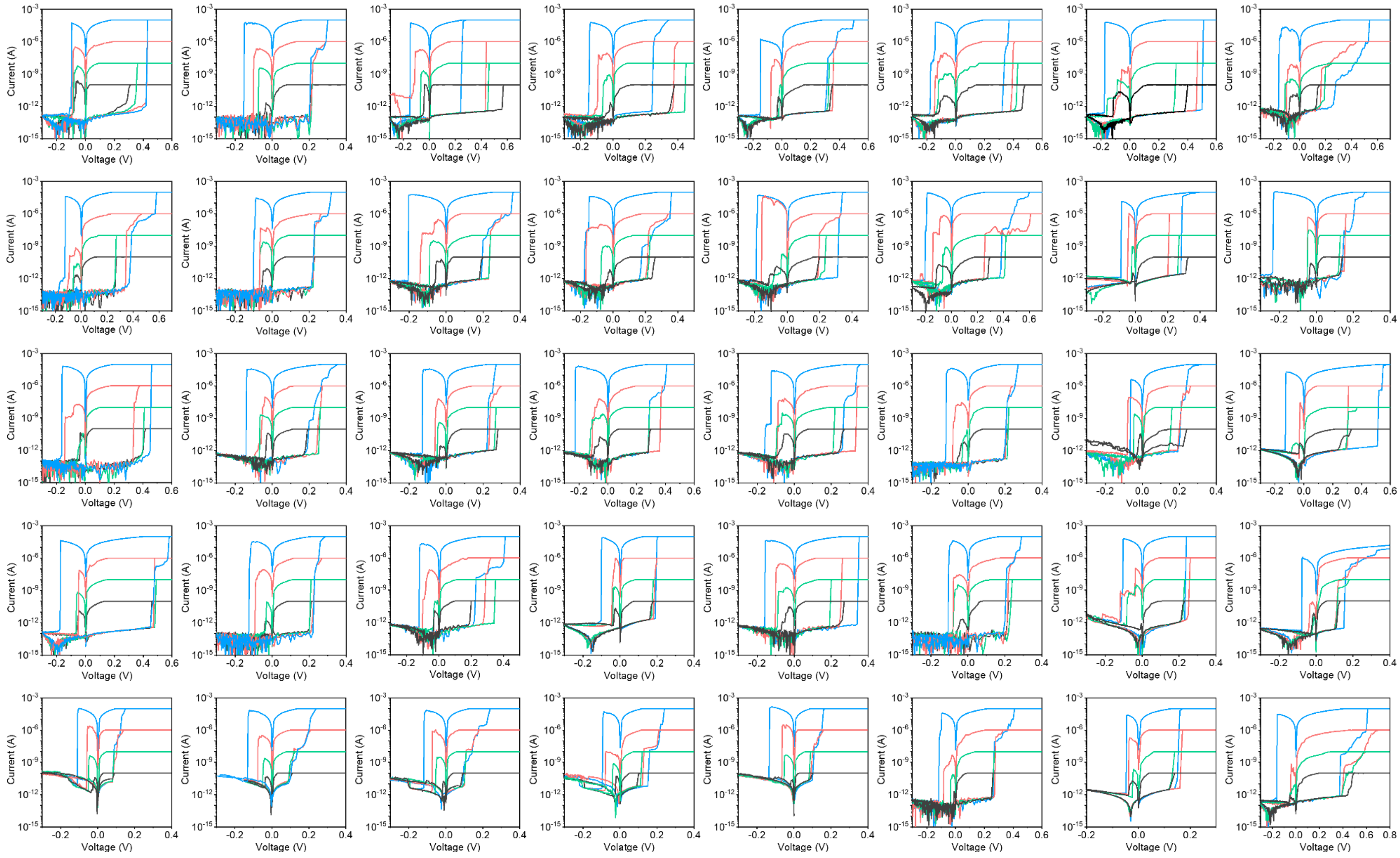

Fig. S24. *I-V* characteristics of devices in the Ag-IPS memristor crossbar array. The *I-V* curves of the Ag-IPS memristor in the 10 × 8 Ag-IPS crossbar array were measured under different operation currents (100 pA, 10 nA, 1 μA, and 100 μA). All the devices show nonvolatile RS switching under different operation currents.

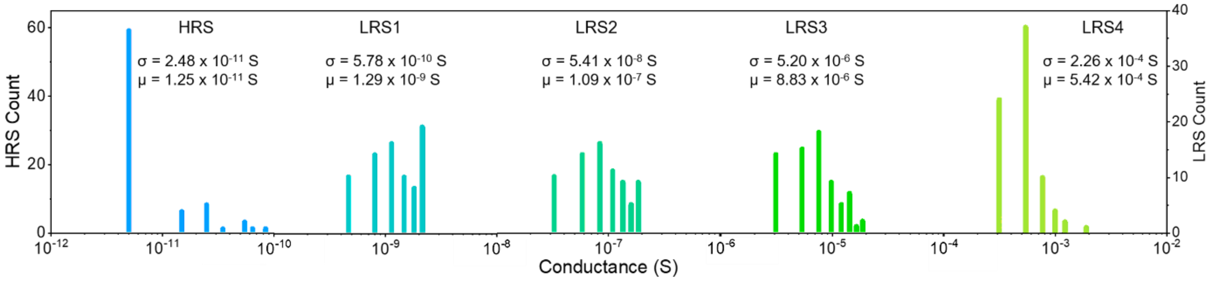

Fig. S25 Histogram of the resistance distribution of the five states (HRS and LRS1, LRS2, LRS3, and LRS4). The five states are well-distinguished without overlap. Except for the HRS resistance, the ranges of LRS1, LRS2, LRS3, and LRS4 resistances are within one order of magnitude, i.e., the $G_{\text{Maxmum-LRS}}/G_{\text{Minimum-LRS}} < 10$. The device-to-device variation is higher than the cycle-to-cycle variation, which may be due to the thickness fluctuation and local wrinkles in the switching medium. The further improvement of both the material synthesis and fabrication process would be effective in reducing the variation.

Supplementary Note 4: convolutional image processing using the Ag-IPS memristor crossbar array.

Based on the crossbar array structure and corresponding measured conductance (G) data of the five states (HRS and LRS1, LRS2, LRS3, and LRS4), the convolutional image processing was simulated. Image processing with varied kernel sizes was first conducted. Two memristors were used as a differential pair to represent both positive and negative weight values; thus, the weights of −1/+1 could be mapped by the HRS and LRS, i.e., $G^{\text{HRS}}-G^{\text{LRS1}}$, $G^{\text{HRS}}-G^{\text{LRS2}}$, $G^{\text{HRS}}-G^{\text{LRS3}}$, or $G^{\text{HRS}}-G^{\text{LRS4}} \sim -1$, and $G^{\text{LRS1}}-G^{\text{HRS}}$, $G^{\text{LRS2}}-G^{\text{HRS}}$, $G^{\text{LRS3}}-G^{\text{HRS}}$, or $G^{\text{LRS4}}-G^{\text{HRS}} \sim +1$. Consequently, two 3×3 (or 5×5 or 7×7) crossbar arrays can be used to construct the target 3×3 (or 5×5 or 7×7) G convolution kernel (*33*), as illustrated in Fig.S26. The conductance values are from the conductance of the memristors at the corresponding position of the crossbar array. Fig. 5B shows the spatial conductance mapping that corresponds to the memristors in the crossbar array. When constructing the G convolution kernel, we obtained the conductance from the conductance map according to the position and resistive state (HRS, LRS1, LRS2, LRS3, or LRS4) of the memristors. In other words, the G convolution kernels were mapped from the conductance of the actual devices in the crossbar array. The conversion of the image intensity into voltages, convolutional operation of voltage matrix and G convolution kernel, and conversion of the convolution results into pixels were performed using the software code. Any one of the four LRS states (LRS1-LRS4) is sufficient to construct the convolution kernel with the HRS state to implement the mean softening and embossment processing. With different conductance kernels in different sizes, the image mean softening and enhancement were realized, as shown in Fig. 5D and Fig. S27, which indicates that the G convolutional kernels with different sizes constructed by our memristor crossbar array can implement the image processing. A transistor may be required to connect the memristor to suppress the half-selection and sneak current issues when experimentally operating the memristor crossbar array.

In Fig. S28, the parallel image processing process is demonstrated using a similar strategy. A two-column array of 9×2 instead of $3 \times 3 \times 2$ was used to implement the 3×3 convolutional kernel, where one column represents the sum of the products of the positive values in the filter (I^+), and the other column represents the sum of the products of the negative values in the filter (I^-)(*33*).

The G convolutional kernels were constructed using the measured conductance data of the memristors in the crossbar array (Fig. 5B). Using the entire 9 × 8 size of the array, 4 different convolution kernels for vertical and horizontal edges extraction, edge embossment, and mean soften can be simultaneously built. Fig. S28A shows the corresponding conductance map. Similarly, we used the software code to perform the product and summation of V_i and G_{ij} emulating Ohm's law and Kirchhoff's current law, *i.e.*, I_j^+ or I_j^- = $\Sigma V_i \times G_{ij}$, the sum of differential currents representing the convolutional output, i.e., $I_{\text{out1}} = I_1^+ - I_1^-, I_{\text{out2}} = I_2^+ - I_2^-, I_{\text{out3}} = I_3^+ - I_3^-$, and $I_{\text{out4}} = I_4^+ - I_4^-$, and the final operation of converting I_{out} into image pixel. After performing the convolution operation of the entire image, we achieved the complete extraction results. Fig. 5E and Figs. S28B–E show the mean soften, vertical edge extraction, horizontal edge extraction, and edge embossment and the corresponding pixel distribution. All four image processing operations were successfully implemented, which indicates the potential for parallel image processing by our memristor crossbar array.

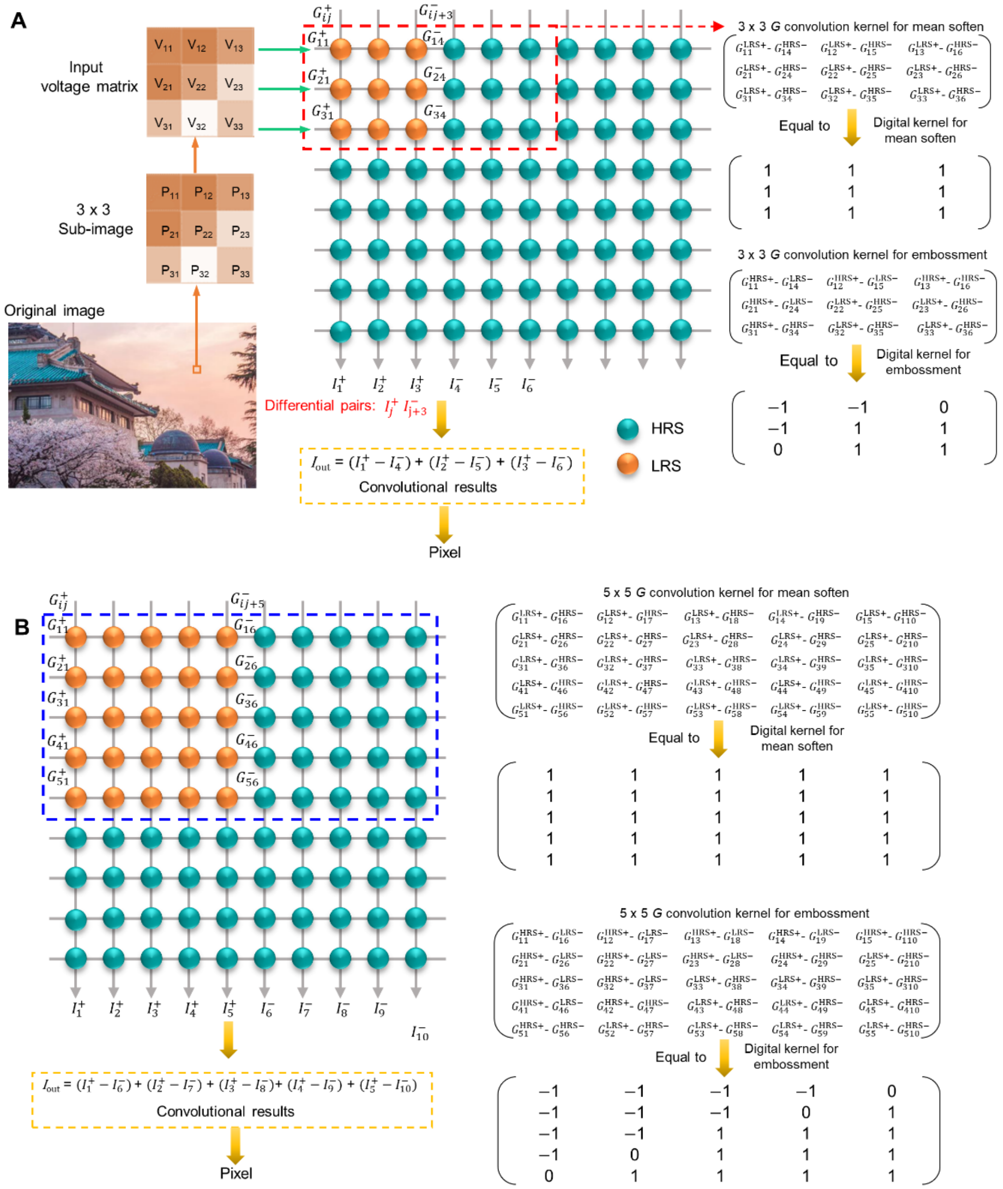

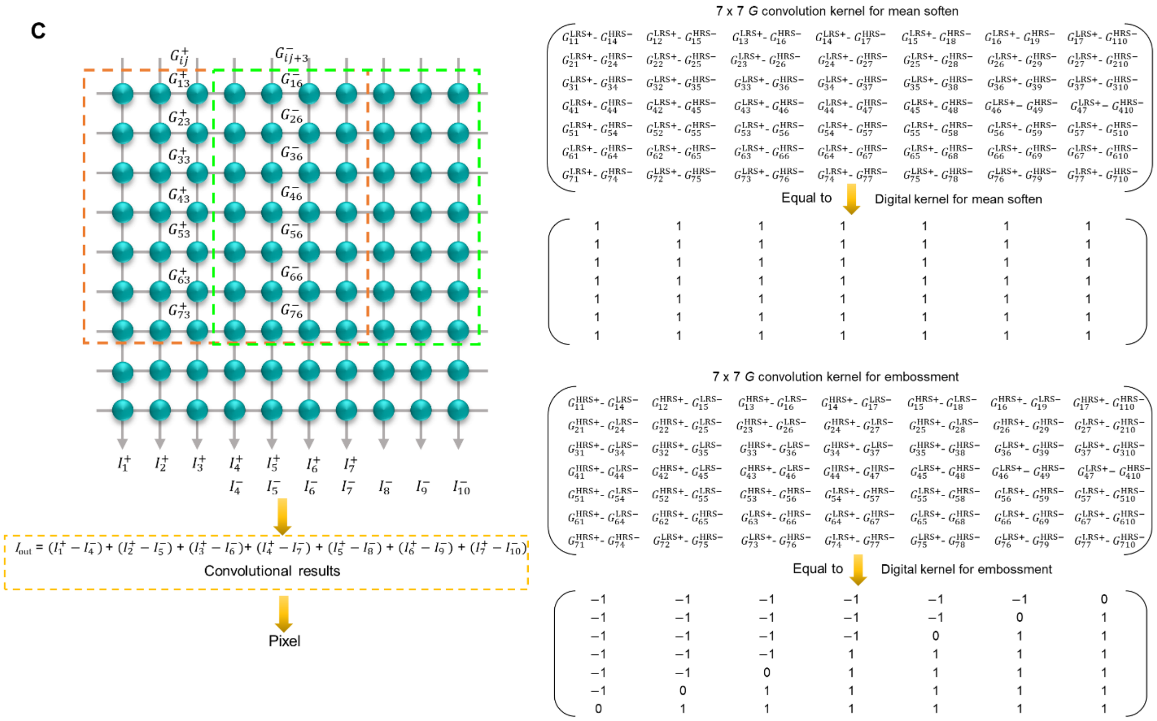

Fig. S26. Illustration of the process of convolutional image processing and the kernels with different sizes constructed by the Ag-IPS memristor crossbar array. (**A**) The process of the convolutional image processing with 3 ×3 kernel mapping by two 3 ×3 memristor crossbar arrays. (**B**) 5 × 5 kernel mapping by two 5 × 5 memristor crossbar arrays. (**C**) 7 × 7 kernel mapping by two 7 ×7 memristor crossbar arrays.

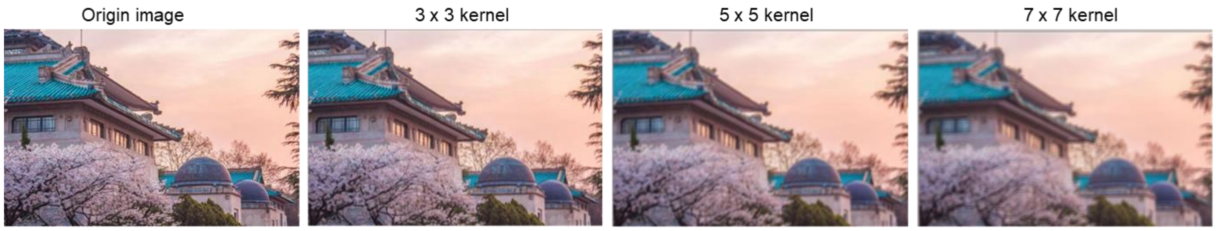

Fig. S27. Comparison of convolutional image processing of mean soften with kernel in different sizes.

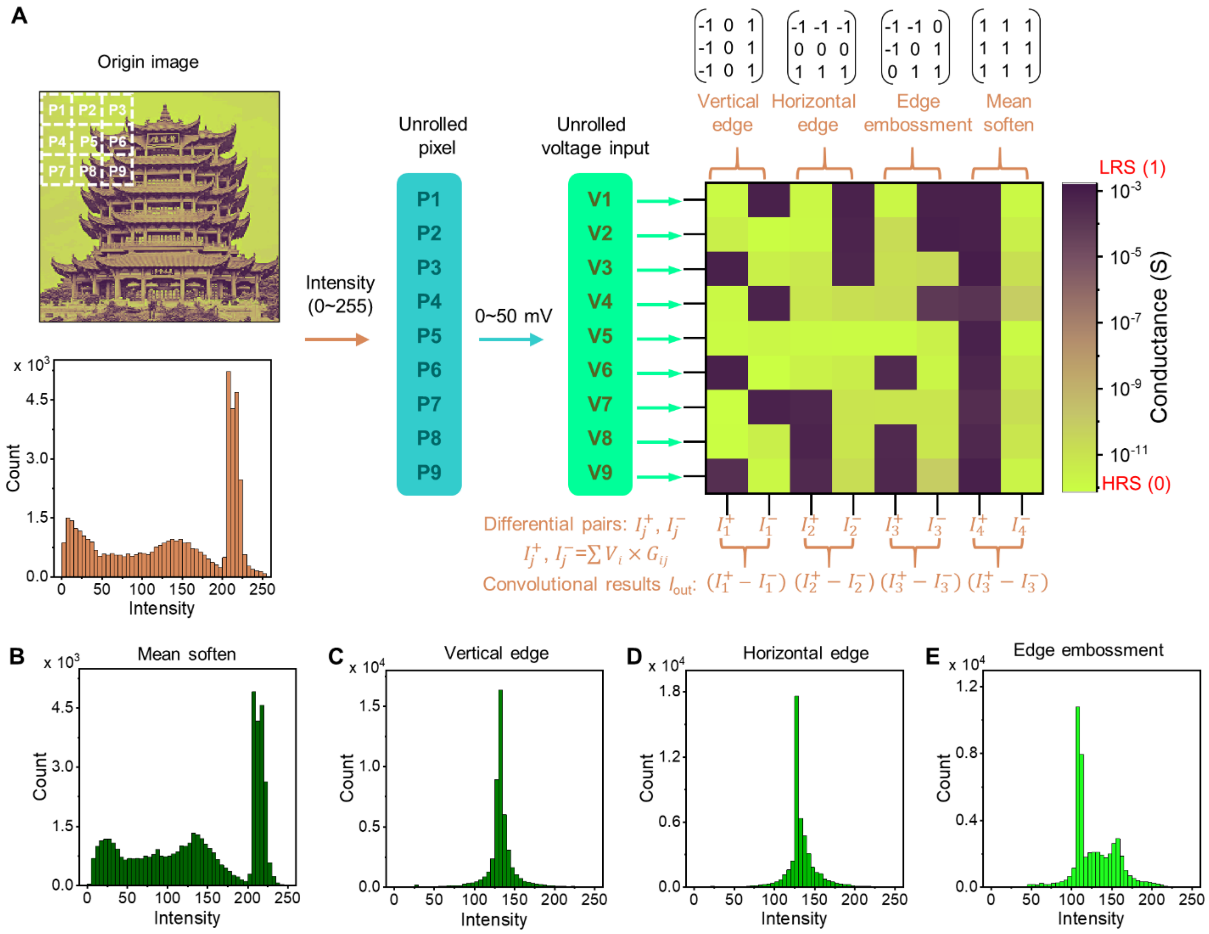

Fig. S28. Parallel convolutional image processing implemented by the Ag-IPS memristor crossbar array. (**A**) The overall process of the parallel convolutional image processing with the memristor crossbar array. The measured conductance data of HRS and LRS4 in Fig. 5B are used to construct the different convolutional kernels. (**B-E**) The histogram of pixel distribution of the parallel image processing results.

REFERENCES

1. N. J. Tye, S. Hofmann, P. Stanley-Marbell, Materials and devices as solutions to computational problems in machine learning. *Nat. Electron.* **6**, 479–490 (2023).

2. AI hardware has an energy problem. *Nat. Electron.* **6**, 463 (2023).

3. M. Zhao, B. Gao, J. Tang, H. Qian, H. Wu, Reliability of analog resistive switching memory for neuromorphic computing. *Appl. Phys. Rev.* **7**, 011301 (2020).

4. M. Lanza, A. Sebastian, W. D. Lu, M. Le Gallo, M.-F. Chang, D. Akinwande, F. M. Puglisi, H. N. Alshareef, M. Liu, J. B. Roldan, Memristive technologies for data storage, computation, encryption, and radio-frequency communication. *Science* **376**, eabj9979 (2022).

5. M. Li, H. Liu, R. Zhao, F.-S. Yang, M. Chen, Y. Zhuo, C. Zhou, H. Wang, Y.-F. Lin, J. J. Yang, Imperfection-enabled memristive switching in van der Waals materials. *Nat. Electron.* **6**, 491–505 (2023).

6. M. Lanza, R. Waser, D. Ielmini, J. J. Yang, L. Goux, J. Suñe, A. J. Kenyon, A. Mehonic, S. Spiga, V. Rana, S. Wiefels, S. Menzel, I. Valov, M. A. Villena, E. Miranda, X. Jing, F. Campabadal, M. B. Gonzalez, F. Aguirre, F. Palumbo, K. Zhu, J. B. Roldan, F. M. Puglisi, L. Larcher, T.-H. Hou, T. Prodromakis, Y. Yang, P. Huang, T. Wan, Y. Chai, K. L. Pey, N. Raghavan, S. Dueñas, T. Wang, Q. Xia, S. Pazos, Standards for the characterization of endurance in resistive switching devices. *ACS Nano* **15**, 17214–17231 (2021).

7. Y. Shen, W. Zheng, K. Zhu, Y. Xiao, C. Wen, Y. Liu, X. Jing, M. Lanza, Variability and yield in h-BN-based memristive circuits: The role of each type of defect. *Adv. Mater.* **33**, e2103656 (2021).

8. X. Huang, K. A. Jiang, Y. Niu, R. Wang, D. Zheng, A. Dong, X. Dong, C. Mei, J. Lu, S. Liu, Z. Gan, N. Zhong, H. Wang, Configurable ultra-low operating voltage resistive switching between bipolar and threshold behaviors for Ag/TaO_x/Pt structures. *Appl. Phys. Lett.* **113**, 112103 (2018).

9. P. Lei, H. Duan, L. Qin, X. Wei, R. Tao, Z. Wang, F. Guo, M. Song, W. Jie, J. Hao, High-performance memristor based on 2D layered BiOI nanosheet for low-power artificial optoelectronic synapses. *Adv. Funct. Mater.* **32**, 2201276 (2022).

10. S. Chen, M. R. Mahmoodi, Y. Shi, C. Mahata, B. Yuan, X. Liang, C. Wen, F. Hui, D. Akinwande, D. B. Strukov, M. Lanza, Wafer-scale integration of two-dimensional materials in high-density memristive crossbar arrays for artificial neural networks. *Nat. Electron.* **3**, 638–645 (2020).

11. H. Zhao, Z. P. Dong, H. Tian, D. DiMarzi, M. G. Han, L. H. Zhang, X. D. Yan, F. X. Liu, L. Shen, S.-J. Han, S. Cronin, W. Wu, J. Tice, J. Guo, H. Wang, Atomically thin femtojoule memristive device. *Adv. Mater.* **29**, 1703232 (2017).

12. X. H. Wu, R. J. Ge, P. A. Chen, H. Chou, Z. P. Zhang, Y. F. Zhang, S. Banerjee, M.-H. Chiang, J. C. Lee, D. Akinwande, Thinnest nonvolatile memory based on monolayer h-BN. *Adv. Mater.* **31**, e1806790 (2019).

13. S. Roy, G. Niu, Q. Wang, Y. Wang, Y. Zhang, H. Wu, S. Zhai, P. Shi, S. Song, Z. Song, Z.-G. Ye, C. Wenger, T. Schroeder, Y.-H. Xie, X. Meng, W. Luo, W. Ren, Toward a reliable synaptic simulation using Al-doped HfO_2 RRAM. *ACS Appl. Mater. Interfaces* **12**, 10648–10656 (2020).

14. Y. Wang, M. Cao, J. Bian, Q. Li, J. Su, Flexible ZnO nanosheet-based artificial synapses prepared by low-temperature process for high recognition accuracy neuromorphic computing. *Adv. Funct. Mater.* **32**, 2209907 (2022).

15. J. Park, E. Park, S. Kim, H. Y. Yu, Nitrogen-induced enhancement of synaptic weight reliability in titanium oxide-based resistive artificial synapse and demonstration of the reliability effect on the neuromorphic system. *ACS Appl. Mater. Interfaces* **11**, 32178–32185 (2019).

16. Y. Li, S. Chen, Z. Yu, S. Li, Y. Xiong, M.-E. Pam, Y.-W. Zhang, K.-W. Ang, In-memory computing using memristor arrays with ultrathin 2D $PdSeO_x/PdSe_2$ heterostructure. *Adv. Mater.* **34**, e2201488 (2022).

17. T. Wang, S. Brivio, E. Cianci, C. Wiemer, M. Perego, S. Spiga, M. Lanza, Improving HfO_2-based resistive switching devices by inserting a TaO_x thin film via engineered in situ oxidation. *ACS Appl. Mater. Interfaces* **14**, 24565–24574 (2022).

18. R. Zhang, H. Huang, Q. Xia, C. Ye, X. Wei, J. Wang, L. Zhang, L. Q. Zhu, Role of oxygen vacancies at the TiO_2/HfO_2 interface in flexible oxide-based resistive switching memory. *Adv. Electron. Mater.* **5**, 1800833 (2019).

19. Y.-L. Zhu, K.-H. Xue, X.-M. Cheng, C. Qiao, J.-H. Yuan, L.-H. Li, X.-S. Miao, Uniform and robust TiN/HfO_2/Pt memristor through interfacial Al-doping engineering. *Appl. Surf. Sci.* **550**, 149274 (2021).

20. S. Brivio, J. Frascaroli, S. Spiga, Role of Al doping in the filament disruption in HfO_2 resistance switches. *Nanotechnology* **28**, 395202 (2017).

21. Z. J. Tan, V. Somjit, C. Toparli, B. Yildiz, N. Fang, Electronegative metal dopants improve switching variability in Al_2 O_3 resistive switching devices. *Phys. Rev. Mater.* **6**, 105002 (2022).

22. M. A. Susner, M. Chyasnavichyus, M. A. McGuire, P. Ganesh, P. Maksymovych, Metal thio- and selenophosphates as multifunctional van der Waals layered materials. *Adv. Mater.* **29**, 1602852 (2017).

23. F. M. Wang, T. A. Shifa, P. Yu, P. He, Y. Liu, F. Wang, Z. X. Wang, X. Y. Zhan, X. D. Lou, F. Xia, J. He, New frontiers on van der Waals layered metal phosphorous trichalcogenides. *Adv. Funct. Mater.* **28**, 1802151 (2018).

24. H. Zhou, J. Zhou, S. Wang, P. Li, Q. Li, J. Xue, Z. Zhou, R. Wang, Y. Yu, Y. Weng, F. Zheng, Z. Li, S. Ju, L. Fang, L. You, Size effect on optical and vibrational properties of van der Waals layered $In_{4/3}P_2S_6$. *APL Mater.* **10**, 061111 (2022).

25. A. Krishnaprasad, D. Dev, S. S. Han, Y. Shen, H.-S. Chung, T.-S. Bae, C. Yoo, Y. Jung, M. Lanza, T. Roy, MoS_2 synapses with ultra-low variability and their implementation in boolean logic. *ACS Nano* **16**, 2866–2876 (2022).

26. M. Ismail, C. Mahata, O. Kwon, S. Kim, Neuromorphic synapses with high switching uniformity and multilevel memory storage enabled through a Hf-Al-O alloy for artificial intelligence. *ACS Appl. Electron. Mater.* **4**, 1288–1300 (2022).

27. F. Wan, Q. Wang, T. Harumoto, T. Gao, K. Ando, Y. Nakamura, J. Shi, Truly electroforming-free memristor based on TiO_2-CoO phase-separated oxides with extremely high uniformity and low power consumption. *Adv. Funct. Mater.* **30**, 2007101 (2020).

28. K. Wang, L. Li, R. Zhao, J. Zhao, Z. Zhou, J. Wang, H. Wang, B. Tang, C. Lu, J. Lou, J. Chen, X. Yan, A pure 2H-MoS_2 nanosheet-based memristor with low power consumption and linear multilevel storage for artificial synapse emulator. *Adv. Electron. Mater.* **6**, 1901342 (2020).

29. Z. Ma, J. Ge, W. Chen, X. Cao, S. Diao, Z. Liu, S. Pan, Reliable memristor based on ultrathin native silicon oxide. *ACS Appl. Mater. Interfaces* **14**, 21207–21216 (2022).

30. J.-S. Yao, J. Ge, B.-N. Han, K.-H. Wang, H.-B. Yao, H.-L. Yu, J.-H. Li, B.-S. Zhu, J.-Z. Song, C. Chen, Q. Zhang, H.-B. Zeng, Y. Luo, S.-H. Yu, Ce^{3+}-doping to modulate photoluminescence kinetics for efficient $CsPbBr_3$ nanocrystals based light-emitting diodes. *J. Am. Chem. Soc.* **140**, 3626–3634 (2018).

31. J. Li, S. Hou, Y.-R. Yao, C. Zhang, Q. Wu, H.-C. Wang, H. Zhang, X. Liu, C. Tang, M. Wei, W. Xu, Y. Wang, J. Zheng, Z. Pan, L. Kang, J. Liu, J. Shi, Y. Yang, C. J. Lambert, S.-Y. Xie, W. Hong, Room-temperature logic-in-memory operations in single-metallofullerene devices. *Nat. Mater.* **21**, 917–923 (2022).

32. J. Q. Ai, Y. X. Mao, Q. W. Luo, L. Jia, M. D. Xing, SAR target classification using the multikernel-size feature fusion-based convolutional neural network. *IEEE Trans. Geosci. Remote Sens.* **60**, 5214313 (2022).

33. C. Li, M. Hu, Y. Li, H. Jiang, N. Ge, E. Montgomery, J. Zhang, W. Song, N. Dávila, C. E. Graves, Z. Li, J. P. Strachan, P. Lin, Z. Wang, M. Barnell, Q. Wu, R. S. Williams, J. J. Yang, Q. Xia, Analogue signal and image processing with large memristor crossbars. *Nat. Electron.* **1**, 52–59 (2018).

34. G. Kresse, J. Hafner, Ab initio molecular-dynamics simulation of the liquid-metal-amorphous-semiconductor transition in germanium. *Phys. Rev. B Condens. Matter* **49**, 14251–14269 (1994).

35. P. E. Blöchl, Projector augmented-wave method. *Phys. Rev. B Condens. Matter* **50**, 17953–17979 (1994).

36. B. Hammer, L. B. Hansen, J. K. Norskov, Improved adsorption energetics within density-functional theory using revised Perdew-Burke-Ernzerhof functionals. *Phys. Rev. B* **59**, 7413–7421 (1999).

37. G. Kresse, D. Joubert, From ultrasoft pseudopotentials to the projector augmented-wave method. *Phys. Rev. B* **59**, 1758–1775 (1999).

38. G. Henkelman, H. Jonsson, Improved tangent estimate in the nudged elastic band method for finding minimum energy paths and saddle points. *J. Chem. Phys.* **113**, 9978–9985 (2000).

39. D. Sheppard, R. Terrell, G. Henkelman, Optimization methods for finding minimum energy paths. *J. Chem. Phys.* **128**, 134106 (2008).

40. S. Grimme, J. Antony, S. Ehrlich, H. Krieg, A consistent and accurate ab initio parametrization of density functional dispersion correction (DFT-D) for the 94 elements H-Pu. *J. Chem. Phys.* **132**, 154104 (2010).

41. Y. Li, L. Loh, S. Li, L. Chen, B. Li, M. Bosman, K.-W. Ang, Anomalous resistive switching in memristors based on two-dimensional palladium diselenide using heterophase grain boundaries. *Nat. Electron.* **4**, 348–356 (2021).

42. S. Li, M.-E. Pam, Y. Li, L. Chen, Y.-C. Chien, X. Fong, D. Chi, K.-W. Ang, Wafer-scale 2D hafnium diselenide based memristor crossbar array for energy-efficient neural network hardware. *Adv. Mater.* **34**, e2103376 (2021).

43. M. E. Pam, S. Li, T. Su, Y.-C. Chien, Y. Li, Y. S. Ang, K.-W. Ang, Interface-modulated resistive switching in Mo-irradiated ReS_2 for neuromorphic computing. *Adv. Mater.* **34**, e2202722 (2022).

44. X. F. Lu, Y. Zhang, N. Wang, S. Luo, K. Peng, L. Wang, H. Chen, W. Gao, X. H. Chen, Y. Bao, G. Liang, K. P. Loh, Exploring low power and ultrafast memristor on p-type van der Waals SnS. *Nano Lett.* **21**, 8800–8807 (2021).

45. X. Yan, Q. Zhao, A. P. Chen, J. Zhao, Z. Zhou, J. Wang, H. Wang, L. Zhang, X. Li, Z. Xiao, K. Wang, C. Qin, G. Wang, Y. Pei, H. Li, D. Ren, J. Chen, Q. Liu, Vacancy-induced synaptic behavior in 2D WS_2 nanosheet–based memristor for low-power neuromorphic computing. *Small* **15**, e1901423 (2019).

46. M. Sivan, Y. Li, H. Veluri, Y. Zhao, B. Tang, X. Wang, E. Zamburg, J. F. Leong, J. X. Niu, U. Chand, A. V.-Y. Thean, All WSe_2 1T1R resistive RAM cell for future monolithic 3D embedded memory integration. *Nat. Commun.* **10**, 5201 (2019).

47. X. Yan, C. Qin, C. Lu, J. Zhao, R. Zhao, D. Ren, Z. Zhou, H. Wang, J. Wang, L. Zhang, X. Li, Y. Pei, G. Wang, Q. Zhao, K. Wang, Z. Xiao, H. Li, Robust Ag/ZrO_2/WS_2/Pt memristor for neuromorphic computing. *ACS Appl. Mater. Interfaces* **11**, 48029–48038 (2019).

48. R. Xu, H. Jang, M.-H. Lee, D. Amanov, Y. Cho, H. Kim, S. Park, H.-J. Shin, D. Ham, Vertical MoS_2 double-layer memristor with electrochemical metallization as an atomic-scale synapse with switching thresholds approaching 100 mV. *Nano Lett.* **19**, 2411–2417 (2019).

49. R. J. Ge, X. H. Wu, M. Kim, J. P. Shi, S. Sonde, L. Tao, Y. F. Zhang, J. C. Lee, D. Akinwande, Atomristor: Nonvolatile resistance switching in atomic sheets of transition metal dichalcogenides. *Nano Lett.* **18**, 434–441 (2018).

50. B. Tang, H. Veluri, Y. Li, Z. G. Yu, M. Waqar, J. F. Leong, M. Sivan, E. Zamburg, Y.-W. Zhang, J. Wang, A. V. Y. Thean, Wafer-scale solution-processed 2D material analog resistive memory array for memory-based computing. *Nat. Commun.* **13**, 3037 (2022).

51. H. Jeong, J. Kim, D. Y. Kim, J. Kim, S. Moon, O. F. Ngome Okello, S. Lee, H. Hwang, S.-Y. Choi, J. K. Kim, Resistive switching in few-layer hexagonal boron nitride mediated by defects and interfacial charge transfer. *ACS Appl. Mater. Interfaces* **12**, 46288–46295 (2020).

52. Y. Shi, X. Liang, B. Yuan, V. Chen, H. Li, F. Hui, Z. Yu, F. Yuan, E. Pop, H. S. P. Wong, M. Lanza, Electronic synapses made of layered two-dimensional materials. *Nat. Electron.* **1**, 458–465 (2018).

53. W. Zhang, H. Gao, C. Deng, T. Lv, S. Hu, H. Wu, S. Xue, Y. Tao, L. Deng, W. Xiong, An ultrathin memristor based on a two-dimensional WS_2/MoS_2 heterojunction . *Nanoscale* **13**, 11497–11504 (2021).

54. T. Ahmed, S. Kuriakose, S. A. Tawfik, E. L. H. Mayes, A. Mazumder, S. Balendhran, M. J. S. Spencer, D. Akinwande, M. Bhaskaran, S. Sriram, S. Walia, Mixed ionic-electronic charge transport in layered black-phosphorus for low-power memory. *Adv. Funct. Mater.* **32**, 2107068 (2022).

55. L. Liu, Y. Li, X. D. Huang, J. Chen, Z. Yang, K.-H. Xue, M. Xu, H. W. Chen, P. Zhou, X. S. Miao, Low-power memristive logic device enabled by controllable oxidation of 2D $HfSe_2$ for in-memory computing. *Adv. Sci.* **8**, e2005038 (2021).

56. Y. Xia, J. Wang, R. Chen, H. Wang, H. Xu, C. Jiang, W. Li, X. Xiao, 2D heterostructure of Bi_2O_2Se/Bi_2SeO_x nanosheet for resistive random access memory. *Adv. Electron. Mater.* **8**, 2200126 (2022).

57. V. K. Sahu, A. K. Das, R. S. Ajimsha, P. Misra, Low power high speed 3-bit multilevel resistive switching in TiO_2 thin film using oxidisable electrode. *J. Phys. D Appl. Phys.* **53**, 225303 (2020).

58. Q. Liu, S. Gao, Y. Li, W. Yue, C. Zhang, H. Kan, G. Shen, HfO_2/WO_3 heterojunction structured memristor for high-density storage and neuromorphic computing. *Adv. Mater. Technol.* **8**, 2201143 (2022).

59. Q. Xue, Y. Peng, L. Cao, Y. Xia, J. Liang, C.-C. Chen, M. Li, T. Hang, Ultralow set voltage and enhanced switching reliability for resistive random-access memory enabled by an electrodeposited nanocone array. *ACS Appl. Mater. Interfaces* **14**, 25710–25721 (2022).

60. S. G. Ren, R. Ni, X. D. Huang, Y. Li, K. H. Xue, X. S. Miao, $Pt/Al_2O_3/TaO_X$/ Ta self-rectifying memristor with record-low operation current (< 2 pA), low power (fJ), and high scalability. *IEEE Trans. Electron Devices* **69**, 838–842 (2022).

61. B. K. You, J. M. Kim, D. J. Joe, K. Yang, Y. Shin, Y. S. Jung, K. J. Lee, Reliable memristive switching memory devices enabled by densely packed silver nanocone arrays as electric-field concentrators. *ACS Nano* **10**, 9478–9488 (2016).

62. B. K. You, W. I. Park, J. M. Kim, K.-I. Park, H. K. Seo, J. Y. Lee, Y. S. Jung, K. J. Lee, Reliable control of filament formation in resistive memories by self-assembled nanoinsulators derived from a block copolymer. *ACS Nano* **8**, 9492–9502 (2014).

63. S. Petzold, A. Zintler, R. Eilhardt, E. Piros, N. Kaiser, S. U. Sharath, T. Vogel, M. Major, K. P. McKenna, L. Molina-Luna, L. Alff, Forming-free grain boundary engineered hafnium oxide resistive random access memory devices. *Adv. Electron. Mater.* **5**, 1900484 (2019).

64. E. Shahrabi, C. Giovinazzo, M. Hadad, T. LaGrange, M. Ramos, C. Ricciardi, Y. Leblebici, Switching kinetics control of W-based reram cells in transient operation by interface engineering. *Adv. Electron. Mater.* **5**, 1800835 (2019).

65. J. Wang, L. Li, H. Huyan, X. Pan, S. S. Nonnenmann, Highly uniform resistive switching in HfO_2 films embedded with ordered metal nanoisland arrays. *Adv. Funct. Mater.* **29**, 1808430 (2019).

66. D. S. Kuzmichev, Y. Y. Lebedinskii, C. S. Hwang, A. M. Markeev, Atomic layer deposited oxygen-deficient TaO_x layers for electroforming-free and reliable resistance switching memory. *Phys. Status Solidi Rapid Res. Lett.* **12**, 1800429 (2018).

67. X. Guo, Q. Wang, X. Lv, H. Yang, K. Sun, D. Yang, H. Zhang, T. Hasegawa, D. He, SiO_2/Ta_2O_5 heterojunction ECM memristors: Physical nature of their low voltage operation with high stability and uniformity. *Nanoscale* **12**, 4320–4327 (2020).

68. C.-Y. Zhu, J.-K. Qin, P.-Y. Huang, H.-L. Sun, N.-F. Sun, Y.-L. Shi, L. Zhen, C.-Y. Xu, 2D indium phosphorus sulfide ($In_2P_3S_9$): An emerging van der Waals high-k dielectrics. *Small* **18**, e2104401 (2022).

69. Z. Wang, S. Joshi, S. E. Savel'ev, H. Jiang, R. Midya, P. Lin, M. Hu, N. Ge, J. P. Strachan, Z. Li, Q. Wu, M. Barne, G. L. Li, H. L. Xin, R. S. Williams, Q. Xia, J. J. Yang, Memristors with diffusive dynamics as synaptic emulators for neuromorphic computing. *Nat. Mater.* **16**, 101–108 (2017).

70. Z. Wang, M. Rao, R. Midya, S. Joshi, H. Jiang, P. Lin, W. Song, S. Asapu, Y. Zhuo, C. Li, H. Wu, Q. Xia, J. J. Yang, Threshold switching of Ag or Cu in dielectrics: Materials, mechanism, and applications. *Adv. Funct. Mater.* **28**, 1704862 (2018).

71. J.-H. Cha, S. Y. Yang, J. Oh, S. Choi, S. Park, B. C. Jang, W. Ahn, S.-Y. Choi, Conductive-bridging random-access memories for emerging neuromorphic computing. *Nanoscale* **12**, 14339–14368 (2020).
